# A sustainable avenue for the synthesis of propargylamines and benzofurans using a Cu-functionalized MIL-101(Cr) as a reusable heterogeneous catalyst

**DOI:** 10.1038/s41598-023-40154-0

**Published:** 2023-08-09

**Authors:** Fillip Kumar Sarkar, Lenida Kyndiah, Sushmita Gajurel, Rajib Sarkar, Samaresh Jana, Amarta Kumar Pal

**Affiliations:** 1 Department of Chemistry, Centre for Advanced Studies, North-Eastern Hill University, Shillong, Meghalaya 793022 India; 2 Department of Chemistry, School of Applied Sciences, KIIT- Deemed to be University, Bhubaneswar, Odisha 751024 India

**Keywords:** Catalysis, Organic chemistry

## Abstract

A heterogeneous copper-catalyzed A^3^ coupling reaction of aldehydes, amines, and alkynes for the synthesis of propargylamines and benzofurans has been developed. Here, the modified metal–organic framework MIL-101(Cr)-SB-Cu complex was chosen as the heterogeneous copper catalyst and prepared via post-synthetic modification of amino-functionalized MIL-101(Cr). The structure, morphology, thermal stability, and copper content of the catalyst were determined by FT-IR, PXRD, SEM, TEM, EDX, TGA, XPS, and ICP-OES. The catalyst shows high catalytic activity for the aforementioned reactions under solvent-free reaction conditions. High yields, low catalyst loading, easy catalyst recovery and reusability with not much shrink in catalytic activity, and a good yield of 82% in gram-scale synthesis are some of the benefits of this protocol that drove it towards sustainability.

## Introduction

**Figure 1:**
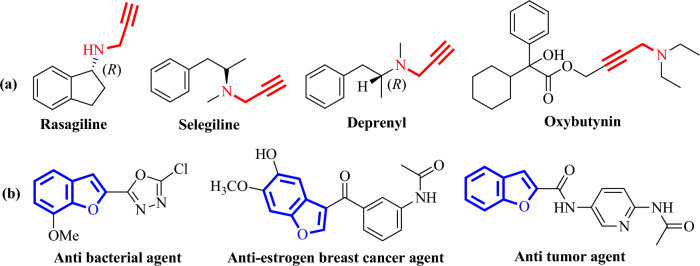
Some biologically important molecules containing propargylamine (**a**) and benzofuran (**b**) moieties.

Propargylamines have wide applications in organic synthesis as they are used as an essential intermediate for the synthesis of various biologically active compounds such as peptides, β-lactams, allylamines, natural products, drug molecules, agrochemical products, etc^1–4^. They are also used as precursors for the synthesis of a variety of heterocyclic compounds such as quinolines^5^, phenanthrolines^6^, pyrroles^6^, etc. In addition, propargylamine scaffolds are found in commercially available drugs such as rasagiline and deprenyl and are also used for the treatment of Parkinson’s and Alzheimer’s disease^7–9^. On the other hand, benzofurans are significant oxygen-containing heterocyclic scaffolds that exhibit immense biological and pharmaceutical activities such as anti-inflammatory^10^, anticancer^11^, antifungal^12^, antitumor^13^, etc. They are not only pivotal structural subunits in naturally occurring bioactive compounds but also act as a useful synthons in the synthesis of many natural products^14–16^. Moreover, benzofurans have several applications in cosmetic formulations and optical brighteners^17^. Therefore, efforts have been made by researchers to develop methodologies to synthesize propargylamines and benzofurans moieties. Some of the reported synthetic procedures for the synthesis of benzofurans are reactions of 2-chlorophenols with terminal alkynes^18^, cyclizations of ketones^19^, the sigmatropic rearrangement of arene^20^, palladium-catalyzed cyclizations of phenols^21^, etc. A recent synthetic approach is the transition-metal catalyzed coupling of aldehydes, amines, and alkynes (known as the A^3^ coupling reaction) which generally produce propargylamines^22–27^. However, benzofurans can also be synthesized by an A^3^ coupling reaction followed by intramolecular cyclizations^28–35^. Therefore, sustainable development of methodologies for the synthesis of these important moieties is of significant interest to organic chemists. Some of the biologically significant molecules containing propargylamines (**a**) and benzofurans (**b**) moieties are shown in Fig. 1.

In the meantime, metal–organic frameworks (MOFs) are notable porous materials that have gained much attention due to their high surface area, ultra-high porosity, thermal stability, tuneable pore size, etc. MOFs have wide applications in drug delivery^36^, sensing^37^, gas storage^38^, waste and water treatment^39–42^, biodiesel production^43^, catalysis^44–47^, etc. The main feature of MOFs is that they can be easily functionalized as per their application by post-synthetic modification (PSM) using suitable organic linkers, keeping the MOF structure intake^48–51^. Among other PSM of MOFs, the condensation between free amine of the MOF and aldehydes to create a Schiff base moiety for incorporation of active metal is the most convenient method^52–57^. Recently, several MOFs have been developed for application in the field of heterogeneous catalysis^58–60^. Therefore, in this study amino-functionalized MIL-101(Cr) has been synthesized and further 2-pyridine-carboxaldehyde was used to create a Schiff base moiety [MIL-101(Cr)-SB] in the framework for anchoring Cu metal with MIL-101(Cr)-SB to make the MIL-101(Cr)-SB-Cu complex, which was then employed for the synthesis of propargylamines and benzofurans via A^3^ coupling and intramolecular cyclization reaction.

## Result and discussion

### Post-synthetic modification of MIL-101(Cr)-NH_2_

**Figure 2:**
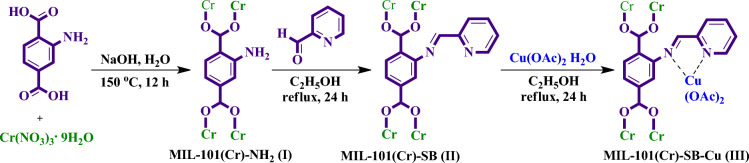
Synthetic route for the preparation of MIL-101(Cr)-SB-Cu (**III**).

The schematic representation for the preparation of MIL-101(Cr)-SB-Cu (**III**) is illustrated in Fig. 2. Initially, MIL-101(Cr)-NH_2_ (**I**) was prepared using Cr(NO_3_)_3_^.^9H_2_O and 2-aminobenzene-1,4-dicarboxylic acid (H_2_N-BDC) by solvothermal method^61^. Thereafter, the functionalization of the free amino group present in the MIL-101(Cr)-NH_2_ (**I**) framework was accomplished by post-synthetic modification (PSM). Accordingly, MIL-101(Cr)-NH_2_ (**I**) was reacted with 2-pyridine-carboxaldehyde for constructing a 2-pyridyl-imine (Schiff base) moiety in the framework for incorporation of Cu species to give MIL-101(Cr)-SB (**II**). Finally, MIL-101(Cr)-SB (**II**) was then treated with Cu(OAc)_2_ to get the expected MIL-101(Cr)-SB-Cu complex (**III**).

### Spectroscopic studies

After the preparation of the MIL-101(Cr)-SB-Cu (**III**), it was well characterized by various spectroscopic techniques such as Fourier transform infrared (FT-IR) spectroscopy, powder X-ray diffraction (PXRD), scanning electron microscopy (SEM), transmission electron microscopy (TEM), energy dispersive X-ray (EDX), X-ray photoelectron spectroscopy (XPS). Thermal stability was determined by thermogravimetric analysis (TGA), and the copper content in the catalyst was examined by inductively coupled plasma optical emission spectroscopy (ICP-OES).

**Figure 3:**
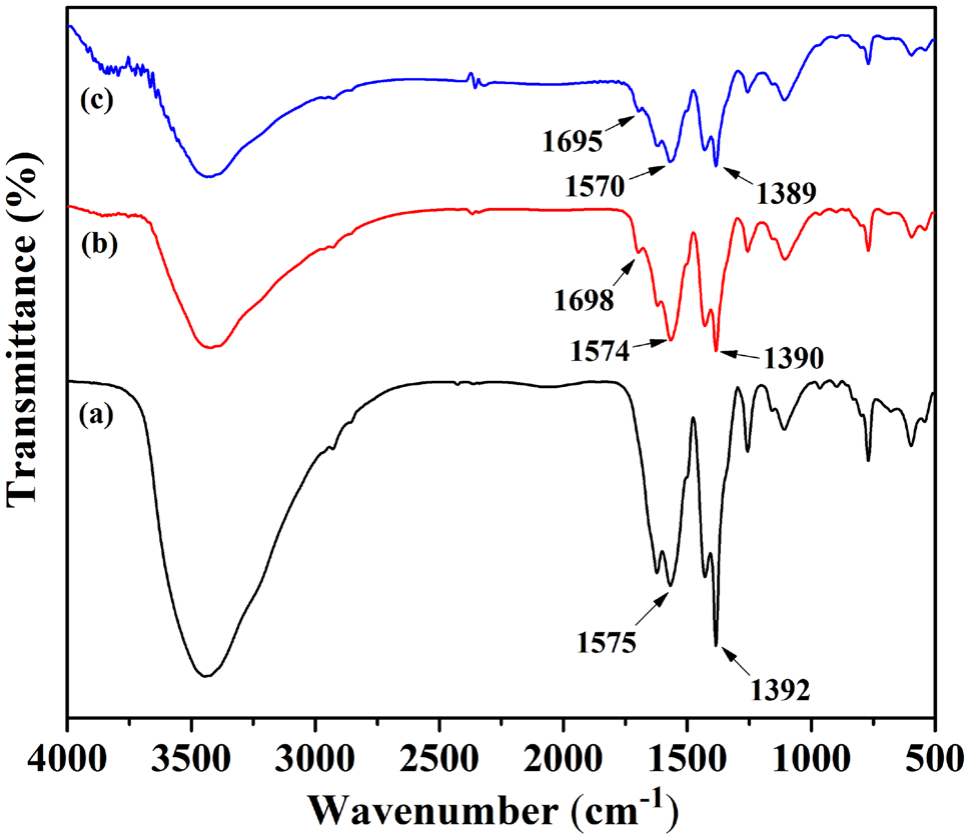
FT-IR spectra of (**a**) MIL-101(Cr)-NH_2_ (**I**), (**b**) MIL-101(Cr)-SB (**II**), and (**c**) MIL-101(Cr)-SB-Cu (**III**).

The FT-IR spectra of the MIL-101(Cr)-NH_2_ (**I**), MIL-101(Cr)-SB (**II**), and MIL-101(Cr)-SB-Cu (**III**) are presented in Fig. 3. In Fig. 3a–c, the characteristic absorption band at around 1390 cm^−1^ is attributed to the O–C–O symmetric vibrations indicating the presence of dicarboxylate linker in all MOF structures. The absorption band at around 1570 cm^−1^ is associated with the C=C stretching vibration of the benzene ring. Further, the band observed at around 1695 cm^−1^ (Fig. 3c) and 1698 cm^−1^ (Fig. 3b) corresponds to the azomethine group (> C=N–) present in the framework. However, a slight shifting of > C=N– band (Fig. 3c) to a lower value endorses the coordination of the Cu^2+^ to the azomethine-N of the Schiff base moiety^62^.

**Figure 4:**
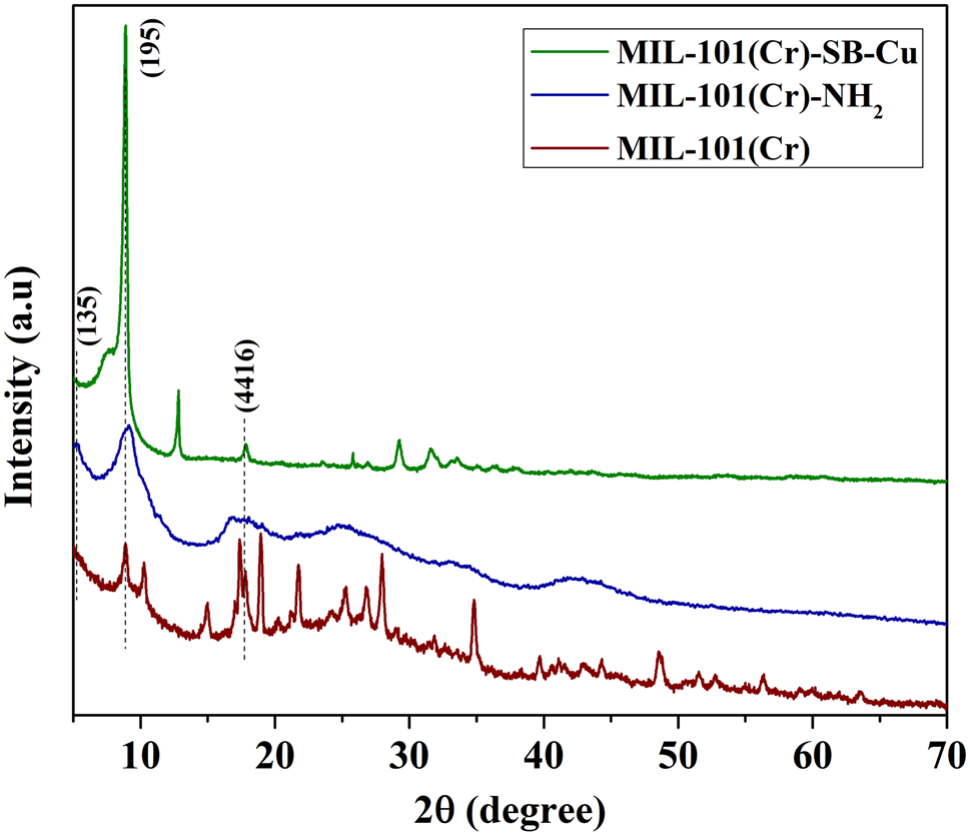
PXRD pattern of MIL-101(Cr), MIL-101(Cr)-NH_2_ (**I**), and MIL-101(Cr)-SB-Cu (**III**).

The PXRD patterns of MIL-101(Cr), MIL-101(Cr)-NH_2_ (**I**), and MIL-101(Cr)-SB-Cu (**III**) are shown in Fig. 4. In the PXRD pattern of MIL-101(Cr), the characteristic diffraction peaks at 2*θ* values 5.4°, 8.9°, 10.3, and 17.3° correspond to (135), (195), (2210), and (4416) planes respectively. Further, the characteristics diffraction peaks of MIL-101(Cr)-NH_2_ at 2*θ* values of 5.5°, 8.8°, and 16.9° correspond to (135), (195), and (4416) planes respectively, which are in accordance with that of the literature and therefore, confirm its successful preparation^55,63–66^. In the PXRD pattern of MIL-101(Cr)-SB-Cu (**III**), the presence of peaks at 2*θ* values of 5.1°, 8.6°, and 17.5° corresponding to MIL-101(Cr)-NH_2_ peaks implies that the structure of the parent MOF is retained even after the post-synthetic modification including functionalization and metal complexation.

**Figure 5:**
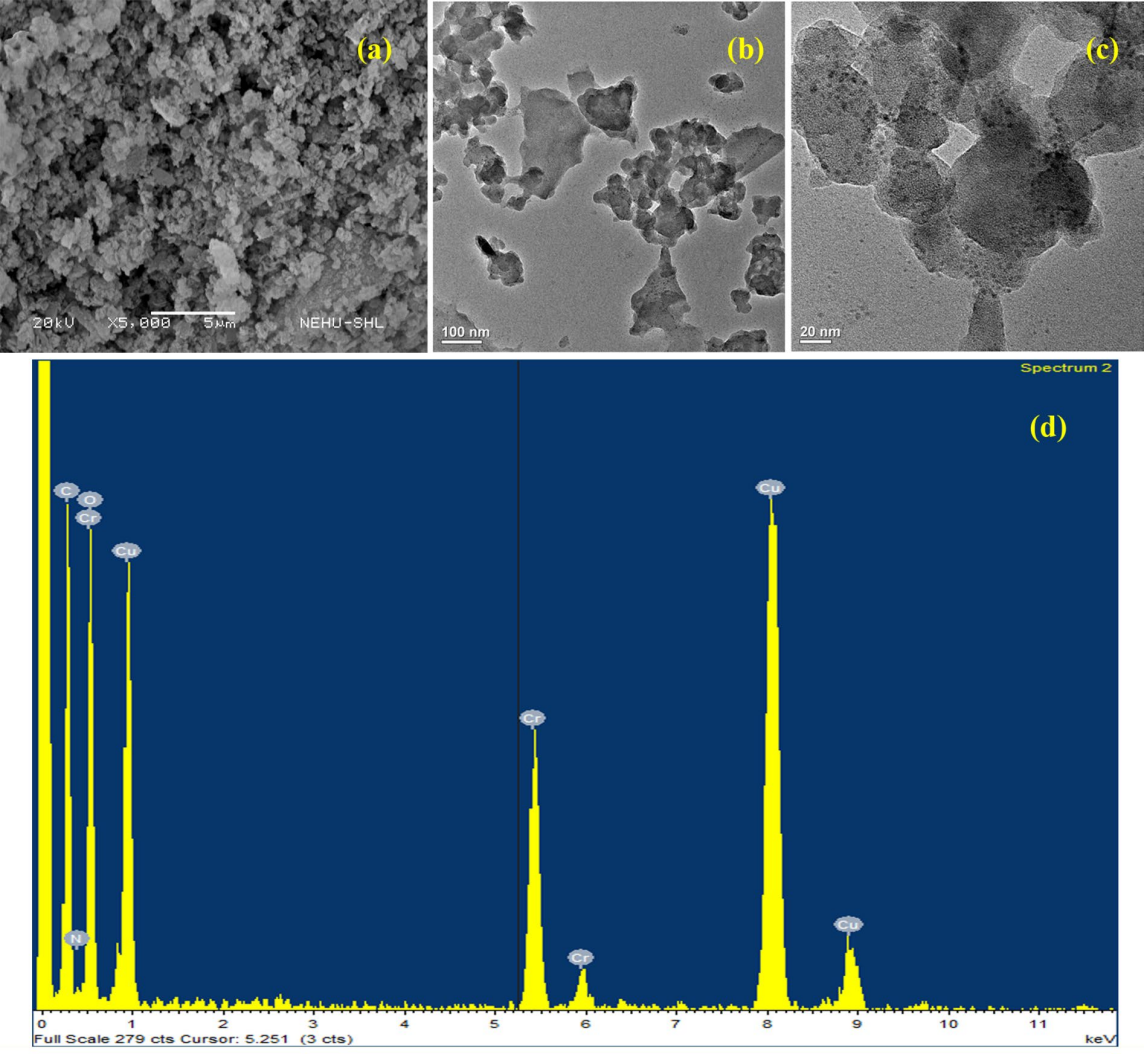
SEM image (**a**), TEM image (**b**,** c**), and EDX spectrum (**d**) of the prepared MIL-101(Cr)-SB-Cu (**III**).

The structural morphology of the prepared MIL-101(Cr)-SB-Cu (**III**) catalyst was investigated using SEM and TEM analysis. The SEM image as depicted in Fig. 5a, shows irregular agglomerated morphology of the prepared MIL-101(Cr)-SB-Cu (**III**) catalyst. The TEM images from low to high magnifications are illustrated in Fig. 5b–c. In the TEM image, the black spherical shape indicates the agglomeration of the Cu in the MOF. The EDX spectrum (Fig. 5d) confirms the presence of all requisite elements such as C, N, O, Cr, and Cu in the prepared MIL-101(Cr)-SB-Cu (**III**) catalyst.

**Figure 6:**
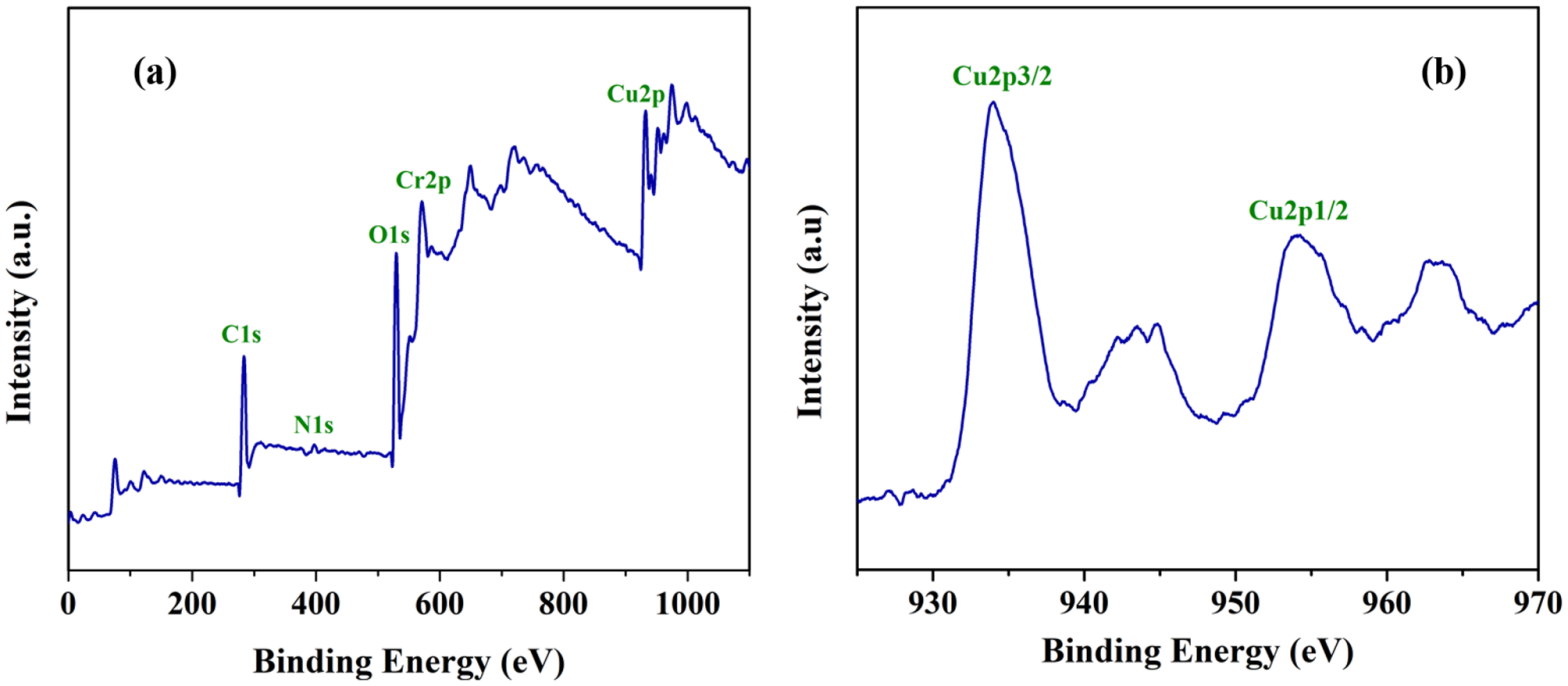
XPS spectra of (**a**) survey, (**b**) Cu 2p scan of the prepared MIL-101(Cr)-SB-Cu (**III**) catalyst.

The oxidation state of Cu species in the MIL-101(Cr)-SB-Cu (**III**) catalyst was determined by XPS analysis. The XPS spectra of the MIL-101(Cr)-SB-Cu (**III**) catalyst are demonstrated in Fig. 6. The survey spectrum as shown in Fig. 6a displays the peaks for C 1s, N 1s, O 1s, Cr 2p, and Cu 2p indicating the successful formation of MIL-101(Cr)-SB-Cu. In the Cu 2p scan (Fig. 6b), the peaks around 933.9 and 953.7 eV corresponds to Cu 2p_3/2_ and Cu 2p_1/2_ respectively, which indicates that the oxidation state of Cu is + 2. Besides these, the satellite peaks at 942.7 and 962.6 eV in Cu 2p also indicate the presence of Cu(II) species^33,67^.

**Figure 7:**
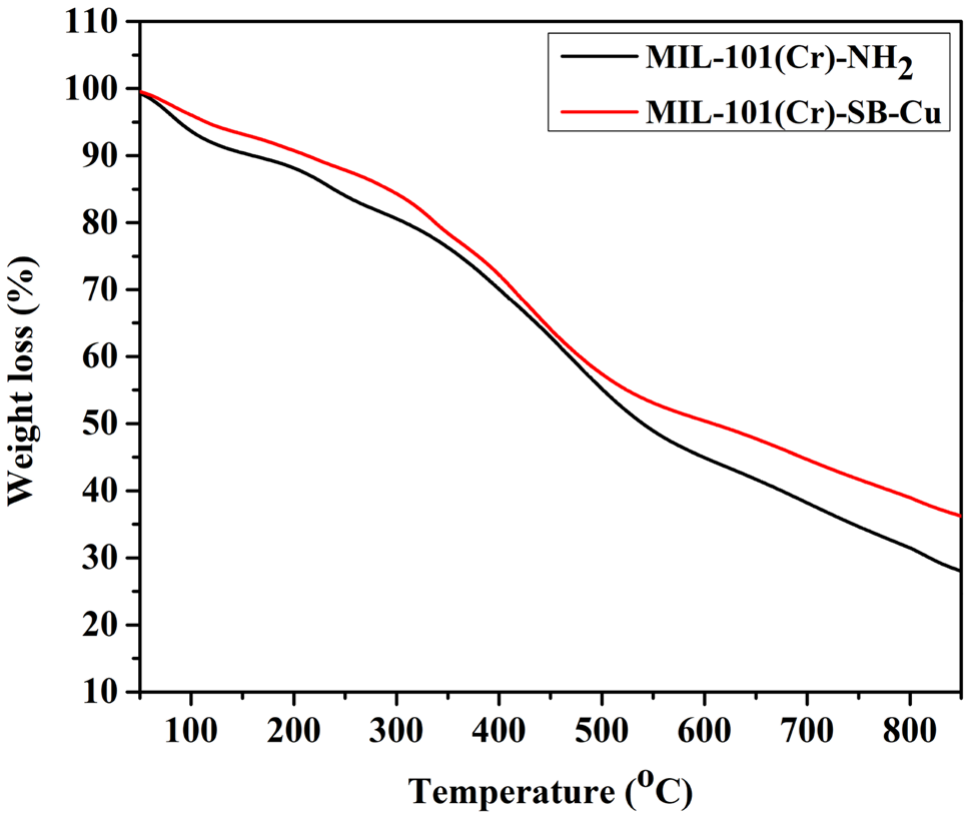
TGA thermogram of MIL-101(Cr)-NH_2_ (**I**) and freshly prepared MIL-101(Cr)-SB-Cu (**III**).

The thermal stability of the prepared MIL-101(Cr)-SB-Cu (**III**) catalyst was studied using thermogravimetric analysis. The TGA thermogram of MIL-101(Cr)-NH_2_ (**I**) and MIL-101(Cr)-SB-Cu (**III**) are demonstrated in Fig. 7. The TGA thermogram of the MIL-101(Cr)-NH_2_ (**I**) exhibits weight loss at around 100 °C which is due to the loss of absorbed water. A major weight loss process is observed within the temperature 250–500 °C which may be due to the decomposition of the MOF structure. The data is similar to that of the reported value which indicates that the MIL-101(Cr)-NH_2_ (**I**) is stable up to 250 °C^68^. Similarly, the TGA thermogram of catalyst (**III**) is also consistent with that of the parent MIL-101(Cr)-NH_2_ (**I**) suggesting that the thermal stability of the catalyst (**III**) is sustained even after the post-synthetic modification and functionalization of the MOF. Furthermore, ICP-OES analysis was carried out to check the Cu content in the prepared MIL-101(Cr)-SB-Cu (**III**) catalyst which was found to be 4.23 wt%.

### Catalytic activity studies

The prepared MIL-101(Cr)-SB-Cu was then utilized for catalytic application. At first, its catalytic activity was studied for the synthesis of propargylamines via A^3^ coupling reaction where 4-methylbenzaldehyde (**1a**), morpholine (**2a**), and phenylacetylene (**3a**) were used as the model substrates for screening different reaction conditions. Initially, the reaction was carried out with MIL-101(Cr)-SB-Cu (15 mg) in toluene under refluxed condition, and the propargylamine product **4a** was obtained in 82% yield within 1 h (Table 1, **entry 1**). When the reaction was performed in solvent-free reaction condition (SFRC) at 100 °C, product **4a** was obtained in 86% yield (Table 1, **entry 2**). Further, screening different solvents such as EtOH, CH_3_CN, DCE, and H_2_O afforded 39%, 47%, 35%, and 19% yields respectively (Table 1, **entries 3–6**). However, CHCl_3_ did not produce the desired product **4a** (Table 1, **entry 7**). Therefore, SFRC was chosen as the optimum condition. Next, the model reaction was performed with different catalyst amounts from 0 to 20 mg under SFRC (Table 1, **entry 8–11**). The best yield of 86% of product **4a** was obtained with 15 mg of the catalyst. Furthermore, increasing the amount of catalyst to 20 mg, there was no increment in the yield of product **4a**. On the other hand, a lower amount of catalyst (< 15 mg) furnished a lower yield of the product (Table 1, **entries 8** and** 9**). As expected, product **4a** was not formed in the absence of a catalyst (Table 1, **entry 11**). Eventually, at lower temperatures such as 60 °C, and 80 °C, the lower yield of product **4a** was obtained (Table 1, **entries 12** and **13**). Thereafter, increasing the temperature to 120 °C also did not enhance the yield of product **4a** (Table 1, **entry 14**). Moreover, MIL-101(Cr)-NH_2_ and MIL-101(Cr) also did not produce the desired product **4a** (Table 1, **entry 15**).

**Table 1:** Optimization of reaction conditions for the synthesis of propargylamines^a^.


Entry	Catalyst	Solvent	Temp. (°C)	Yields 4a (%)^b^
1	(15 mg)	Toluene	Reflux	82
**2**	(15 mg)	–	100	86
3	(15 mg)	EtOH	Reflux	39
4	(15 mg)	CH_3_CN	Reflux	47
5	(15 mg)	DCE	Reflux	35
6	(15 mg)	H_2_O	Reflux	19
7	(15 mg)	CHCl_3_	Reflux	–
8	(5 mg)	–	100	59
9	(10 mg)	–	100	74
10	(20 mg)	–	100	86
11	–	–	100	–
12	(15 mg)	–	60	51
13	(15 mg)	–	80	78
14	(15 mg)	–	120	86
15^c^	(15 mg)	–	100	–
16^d^	(15 mg)	–	100	–

^a^**Reaction Condition: 1a** (1 mmol), **2a** (1.2 mmol), **3a** (1.5 mmol), MIL-101(Cr)-SB-Cu, Solvents (3 mL), 1 h.

^b^Isolated yield.

^c^MIL-101(Cr)-NH_2_.

^d^MIL-101(Cr).

**Figure 8:**
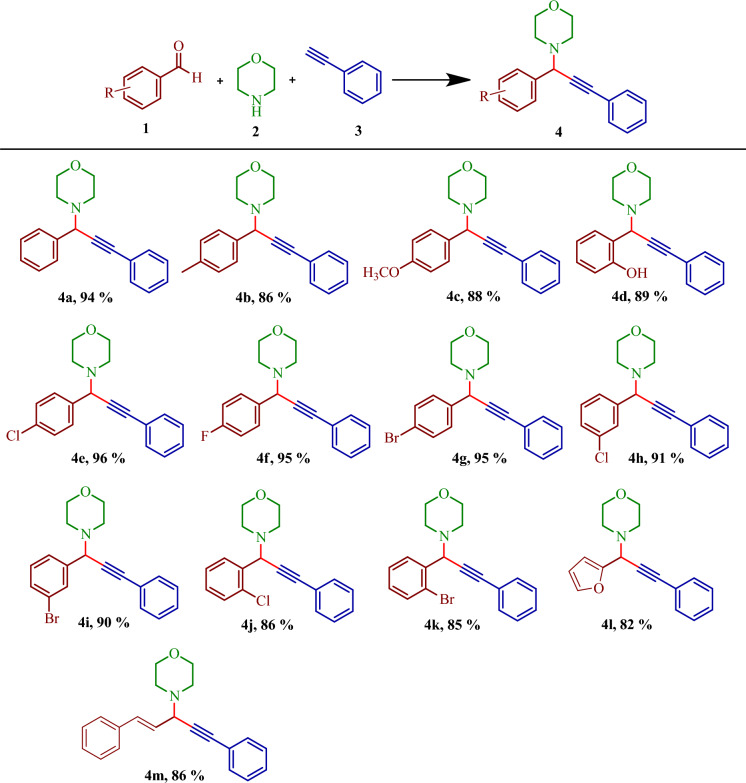
Substrate scope for the synthesis of propargylamines. Reaction Condition: **1** (1 mmol), **2** (1.2 mmol), **3** (1.5 mmol), MIL-101(Cr)-SB-Cu (15 mg), SFRC, 100 °C, 1 h. Isolated yield.

After optimization of the suitable reaction condition, the generality of the methodology was explored using different aryl aldehydes. Aryl aldehydes containing –CH_3_, –OCH_3_, –F, –Cl, –Br afforded the corresponding products in good to excellent yields (85–96%) (Fig. 8, **4a**–**4k**). Further, 2-furaldehyde and trans-cinnamaldehyde also produced the desired products in good yields (Fig. 8, **4l**, and **4m**). Morpholine as the secondary base and phenylacetylene as the alkynes afforded good yields. However, when primary amine such as aniline was used, no desired product was obtained. Further, piperidine and diethyl amine produced a trace amount of products.

The catalytic activity of MIL-101(Cr)-SB-Cu was further studied for the synthesis of benzofurans where 2-hydroxybenzaldehyde (**1d**), morpholine (**2a**), and phenylacetylene (**3a**) were chosen as the model substrates for optimization of the reaction condition. Therefore, to optimize the reaction condition, different solvents, temperature, and catalytic loading were investigated and the results are summarized in Table 2. Initially, the reaction was performed with MIL-101(Cr)-SB-Cu (15 mg) in the presence of DMAP in toluene under refluxed condition, and the benzofuran **5a** was obtained in 84% yield with a trace amount of propargylamine **4d** (Table 2, **entry 1**). However, in the absence of DMAP, the propargylamine **4d** was obtained in 87% yield and failed to produce the benzofuran **5a** (Table 2, **entry 2**). This might be due to the low nucleophilicity of the hydroxyl group of 2-hydroxybenzaldehyde. Hence, DMAP acts as the base which facilitates the intramolecular nucleophilic attack by abstracting the proton from the hydroxyl group to give the benzofuran products. To inspect the effect of solvent on the model reaction, several solvents including solvent-free reaction conditions (SFRC) were screened. Fortunately, an excellent yield of 92% of product **5a** was achieved in solvent-free reaction condition in 6 h (Table 2, **entry 3**). Further, EtOH afforded product **5a** in trace amount and the uncyclized product **4d** in 42% yield (Table 2, **entry 4**). In the case of CHCl_3_, product **5a** was not formed and only a trace amount of **4d** was observed in TLC (Table 2, **entry 5**). However, solvents like CH_3_CN, DCE, and H_2_O provided **4d** in 45%, 37%, and 20% yields respectively with a trace amount of **5a** (Table 2, **entries 6–8**). Henceforth, the reactions were further performed in SFRC. Thereafter, different catalyst concentration was also examined. In the absence of a catalyst, no product formation was detected (Table 2, **entry 9**). When the reactions were carried out with 5, 10, 15, and 20 mg of the catalyst, product **5a** was obtained in 74%, 86%, 92%, and 92% yields respectively (Table 2, **entries 10–12**). The best result was obtained with 15 mg of the catalyst and further increasing the amount of catalyst no increment in the yield of the product was observed. After that, the effect of different amounts of DMAP on the model reaction was checked and the best result was obtained with 0.5 equiv. of DMAP (Table 2, **entries 13** and **14**). Other bases like Na_2_CO_3_, K_2_CO_3_, and Cs_2_CO_3_ were also screened. Na_2_CO_3_ afforded 26% yield of the product **5a** along with 67% yield of **4d** (Table 2, **entries 15**). In case of K_2_CO_3_ and Cs_2_CO_3_, the products **5a** were obtained in 67% and 81% yields respectively along with trace amount of **4d** in both the cases (Table 2, **entries 16** and **17**). Finally, the impact of temperature was investigated and the highest yield of the desired product **5a** was achieved at 100 °C. Lowering the temperature from 100 to 60 °C afforded a lower yield of the product whereas high temperature (120 °C) did not improve the yield of the product **5a** (Table 2, **entries 18–20**). In summary, the optimum reaction condition for this reaction is 15 mg of the catalyst, 0.5 equiv. of DMAP, SFRC, and 100 °C. Moreover, MIL-101(Cr)-NH_2_ and MIL-101(Cr) did not furnish any cyclized (**5a**) or uncyclized (**4d**) products under the optimized reaction condition. (Table 2, **entries 21** and **22**).

**Table 2:** Optimization of reaction conditions for the synthesis of benzofurans^a^.

	
Entry	Catalyst	Solvent	Base (mmol)	Temp. (°C)	Time (h)	Yields (%)^b^	
						5a	4d
1	(15 mg)	Toluene	DMAP (0.5)	Reflux	12	84	Trace
2	(15 mg)	Toluene	–	Reflux	12	–	87
3	(15 mg)	–	DMAP (0.5)	100	6	92	–
4	(15 mg)	EtOH	DMAP (0.5)	Reflux	6	Trace	42
5	(15 mg)	CHCl_3_	DMAP (0.5)	Reflux	6	–	Trace^c^
6	(15 mg)	CH_3_CN	DMAP (0.5)	Reflux	6	Trace	45
7	(15 mg)	DCE	DMAP (0.5)	Reflux	6	Trace	37
8	(15 mg)	H_2_O	DMAP (0.5)	Reflux	6	Trace	20
9	–	–	DMAP (0.5)	100	6	–	–
10	(5 mg)	–	DMAP (0.5)	100	6	74	Trace
11	(10 mg)	–	DMAP (0.5)	100	6	86	Trace
12	(20 mg)	–	DMAP (0.5)	100	6	92	–
13	(15 mg)	–	DMAP (0.25)	100	6	71	24
14	(15 mg)	–	DMAP (1.0)	100	6	92	–
15	(15 mg)	–	Na_2_CO_3_ (0.5)	100	6	26	67
16	(15 mg)	–	K_2_CO_3_ (0.5)	100	6	67	Trace
17	(15 mg)	–	Cs_2_CO_3_ (0.5)	100	6	81	Trace
18	(15 mg)	–	DMAP (0.5)	60	6	65	Trace
19	(15 mg)	–	DMAP (0.5)	80	6	72	Trace
20	(15 mg)	–	DMAP (0.5)	120	6	92	Trace
21^d^	(15 mg)	–	DMAP (0.5)	100	6	–	–
22^e^	(15 mg)	–	DMAP (0.5)	100	6	–	–

^a^**Reaction Condition: 1d** (1 mmol), **2a** (1.2 mmol), **3a** (1.5 mmol), MIL-101(Cr)-SB-Cu. Solvents (3 mL).

^b^Isolated yield.

^c^Detected by TLC.

^d^MIL-101(Cr)-NH_2_.

^e^MIL-101(Cr).

**Figure 9:**
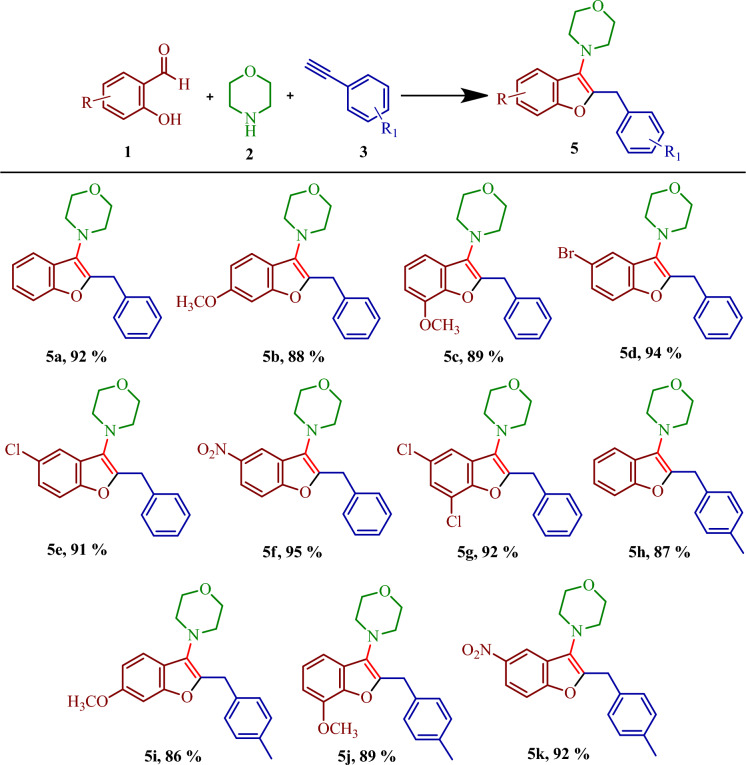
Substrate scope for the synthesis of benzofurans. Reaction Condition: **1** (1 mmol), **2** (1.2 mmol), **3** (1.5 mmol), DMAP (0.5 mmol), MIL-101(Cr)-SB-Cu (15 mg), SFRC, 100 °C, 6 h. Isolated yield.

After optimization of the suitable reaction condition, the versatility of the present protocol was explored with various substituted 2-hydroxybenzaldehyde containing electron-withdrawing groups and electron-donating groups (Fig. 9, **5a**–**5k**). In all the cases, the products were obtained in good to excellent yields (86–95%). Further, electron-withdrawing groups provided slightly better yields of the products (**5f** and **5k**) than electron-donating groups (**5b**, **5c**, **5i**, and **5j**). However, aliphatic alkynes such as 1-octyne, and 1-pentyne did not produce the target products. Morpholine as the secondary amine afforded the desired products in good to excellent yields but aniline, diethyl amine, and piperidine were unable to produce the desired products.

### Plausible mechanism

**Figure 10:**
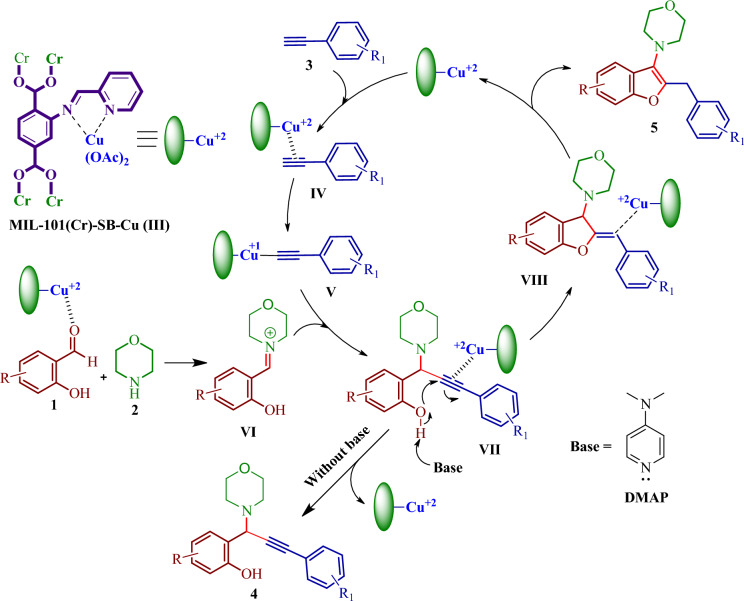
Plausible mechanism for the synthesis of propargylamines and benzofurans.

A schematic representation of a plausible mechanism for the synthesis of propargylamines and benzofurans is presented in Fig. 10^34^. Initially, the alkyne **3** is activated by coordination with the Cu-center of the catalyst to form the copper acetylide intermediate **V**. The catalyst also promotes the condensation reaction of salicylaldehydes **1** and secondary amine **2** by activating the carbonyl carbon of the aldehyde which gives the iminium intermediate **VI**. Then, the iminium intermediate **VI** interacts with the copper acetylide intermediate **V** to produce the propargylamine **4**. In the presence of a base, it abstracts the proton from the phenolic hydroxyl group and facilitates intramolecular cyclization to afford the intermediate **VIII**. Finally, tautomerization furnishes the desired benzofuran **5**.

### Scale-up reaction

**Figure 11:**
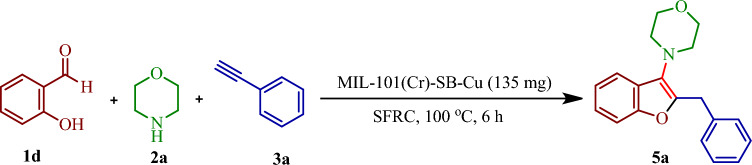
Gram-scale synthesis of benzofurans.

Gram-scale synthesis was carried out to check the possibility of industrial application (Fig. 11). Therefore, the reaction was performed using 2-hydroxybenzaldehyde (**1d**, 9 mmol, 1.080 g), morpholine (**2a**, 10.8 mmol, 0.940 g), phenylacetylene (**3a**, 13.5 mmol, 1.377 g), DMAP (4.5 mmol, 0.549 g) and MIL-101(Cr)-SB-Cu (135 mg) under the optimized reaction condition. After that, the reaction mixture was purified and the desired product was obtained in 82% yield. This good result refers the protocol for industrial application.

### Reusability of the catalyst

**Figure 12:**
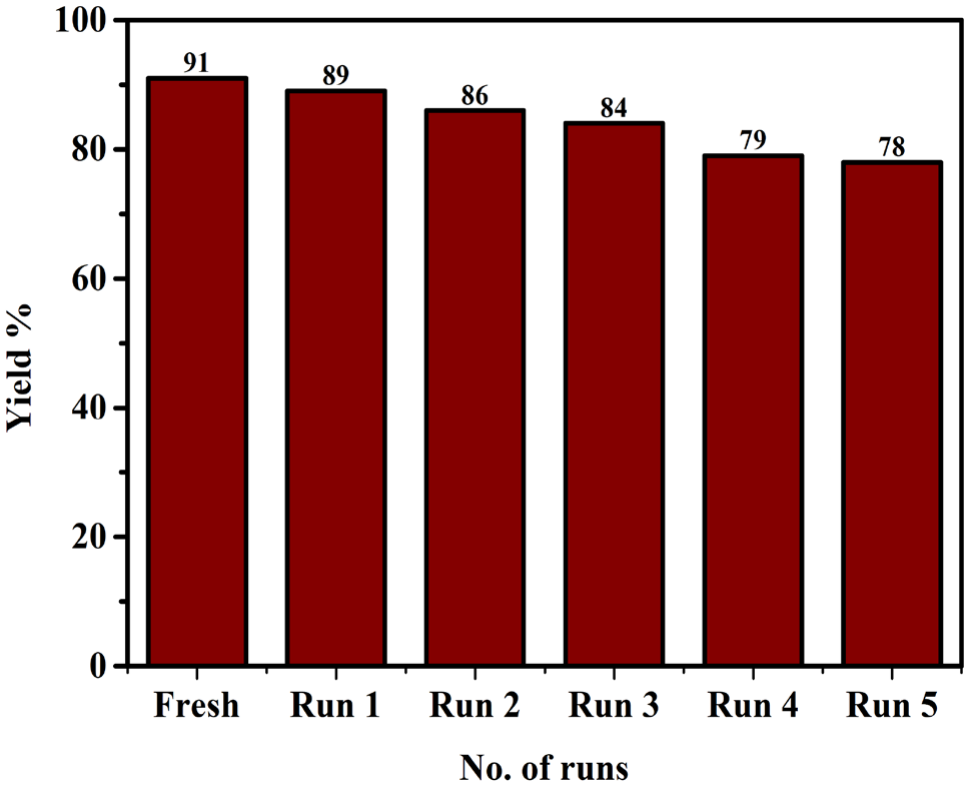
Reusability of MIL-101(Cr)-SB-Cu (**III**) for the synthesis of benzofurans.

Easy separation and reusability of the catalyst are important parameters of a heterogeneous catalyst. Therefore, the reusability of the catalyst was studied for the synthesis of benzofuran (**5a**) using 2-hydroxybenzaldehyde (**1d**), morpholine (**2a**), and phenylacetylene (**3a**) under the optimized reaction condition. After 6 h, ethyl acetate was added to the reaction mixture, and the catalyst was separated by centrifugation followed by filtration. The recovered catalyst was washed with ethyl acetate, ethanol, and diethyl ether and dried properly. Thereafter the recovered catalyst was used in another set of reactions. The catalyst was reused for up to five consecutive runs. It was noticed that the recovered catalyst offered good yield till five runs (Fig. 12).

**Figure 13:**
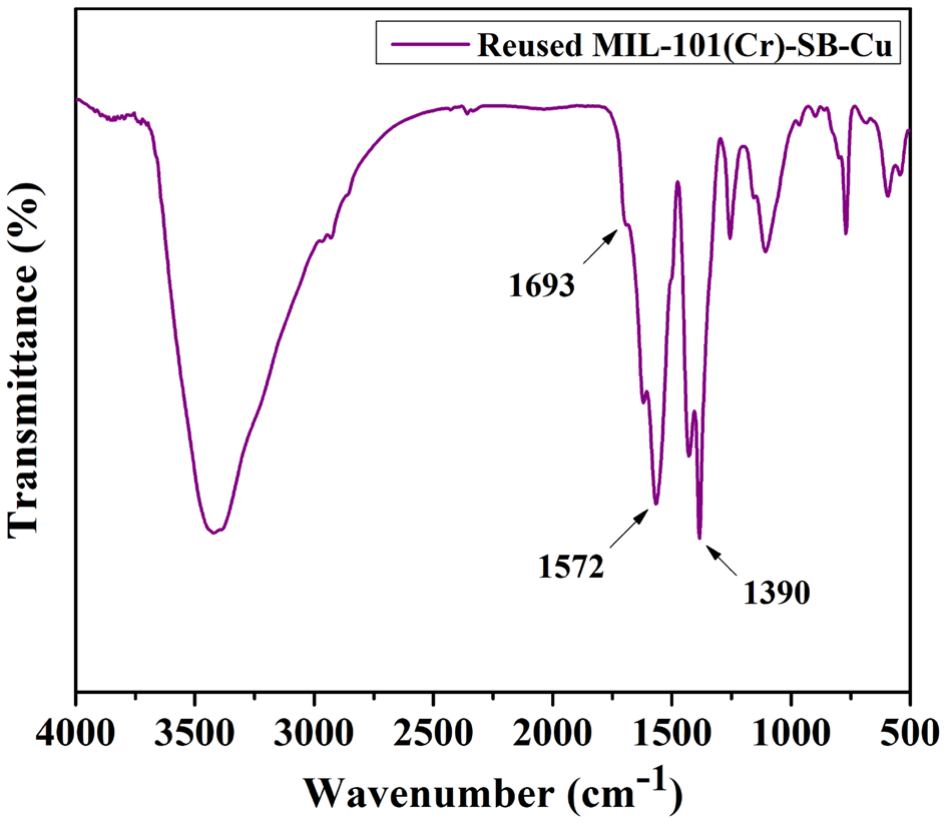
FT-IR spectrum of the reused MIL-101(Cr)-SB-Cu (**III**).

**Figure 14:**
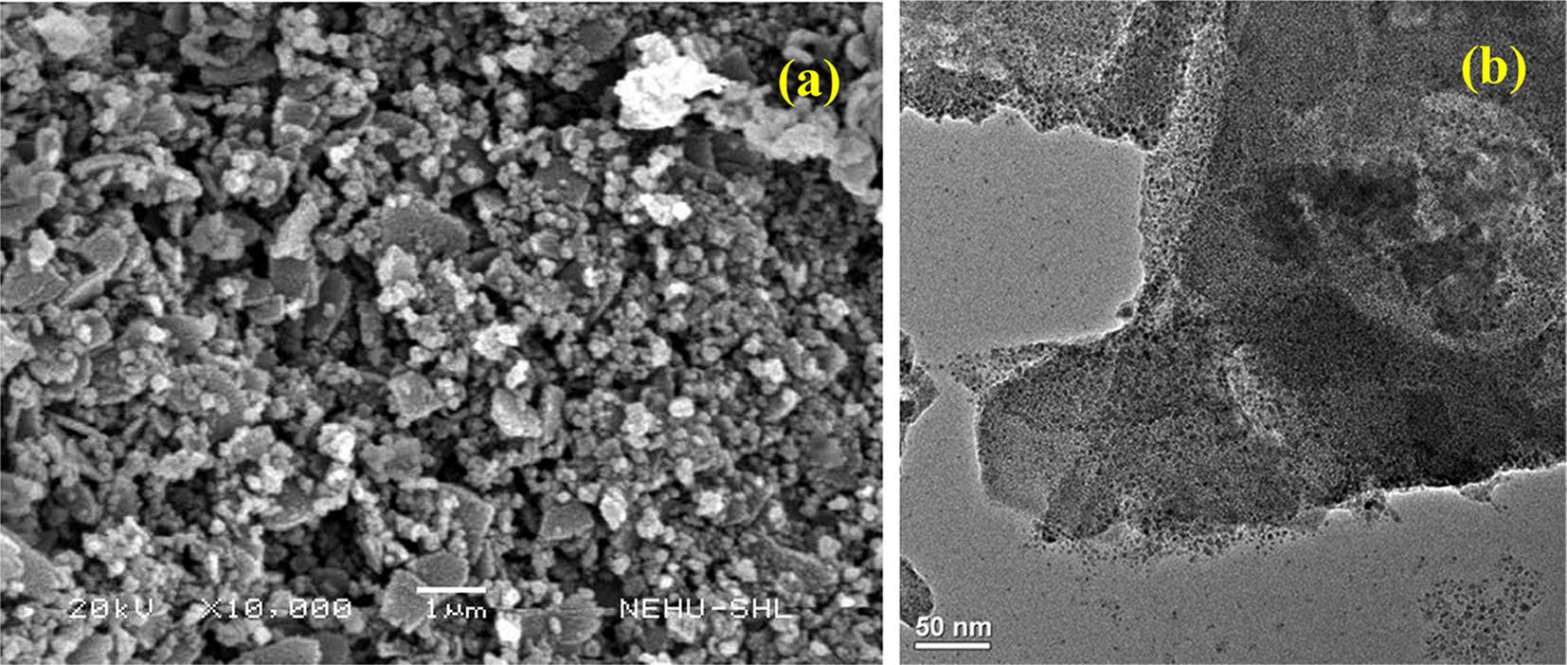
SEM image (**a**) and TEM image (**b**) of the reused MIL-101(Cr)-SB-Cu (**III**).

**Figure 15:**
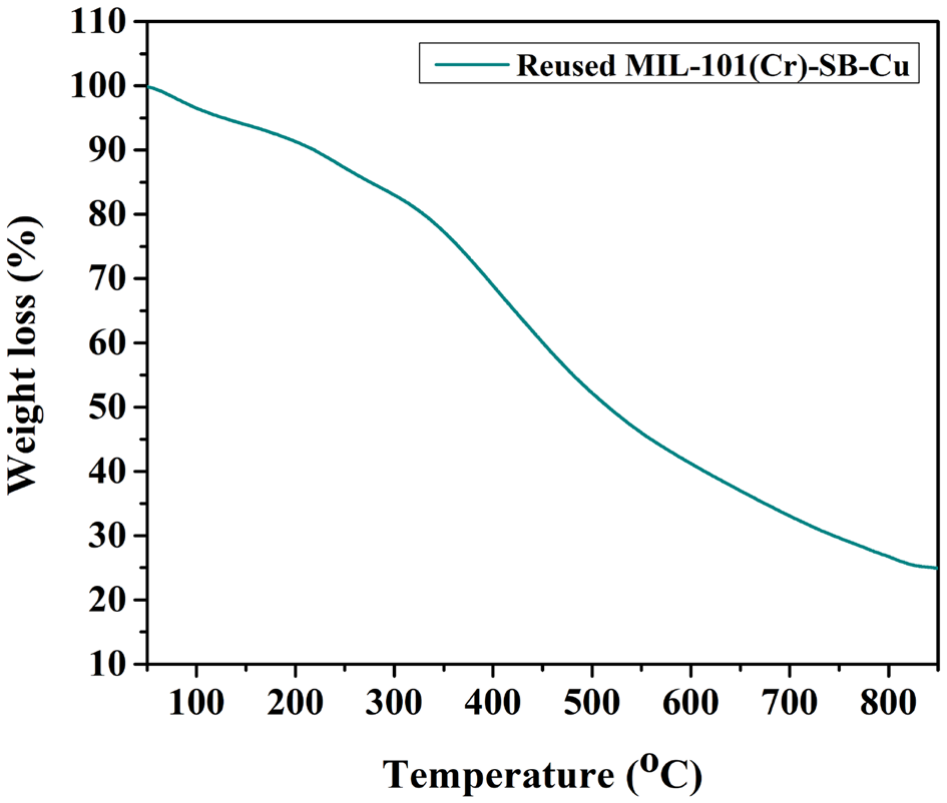
TGA thermogram of the reused MIL-101(Cr)-SB-Cu (**III**).

The structural morphology and stability of the reused MIL-101(Cr)-SB-Cu (**III**) catalyst (after 5th run) were studied using various spectroscopic techniques such as FT-IR (Fig. 13), SEM (Fig. 14a), TEM (Fig. 14b), TGA (Fig. 15), and ICP-OES. From the FT-IR spectrum (Fig. 13) of the reused catalyst no notable changes in the characteristic bands were observed. The SEM (Fig. 14a) and TEM (Fig. 14b) images of the reused catalyst give evidence of some aggregation of the Cu particles. Further, the TGA thermogram (Fig. 15) of the reused catalyst was analogous to that of the fresh catalyst. The leaching of Cu after the 5th cycle was examined by ICP-OES analysis and it was found that only 0.562 ppm Cu leached out.

### Comparative study

The present protocol for the synthesis of benzofurans has been compared with those of previously reported methods in the literature. The comparative summary is illustrated in Table 3. From Table 3, it can be concluded that the present protocol also shows better result as that of the reported methods.

**Table 3:** Comparative study of the present methodology with those reported in the literature for the synthesis of benzofurans.

Entry	Catalyst	Reaction condition	Yield (%)	Refs.
1	Copper(I) oxide NPs	TBAB, K_2_CO_3_, neat, 100 °C, 1.25–6.50 h	79–90	^28^
2	CuI/[bmim]OAc	[bmim]PF_6_, 80 °C, 6–9 h	52–92	^29^
3	CuI	K_2_CO_3_, Bu_4_NBr, toluene, 110 °C, 2–3 h	46–86	^31^
4	CuI	ChCl-EG, 80 °C, 7 h	70–91	^32^
5	CuSO_4_NPs@CMC/PANI	DMAP, toluene, 120 °C, 12–24 h	31–97	^33^
6	h-Fe_2_O_3_@SiO_2_-IL/Ag	Ultrasonic irradiation, 100W, H_2_O, r.t., 20–40 min	85–93	^34^
7	ZnO NPs	Cu(OTf)_2_, K_2_CO_3_, H_2_O/EtOH (1:1), reflux, 1–2 h	80–96	^17^
8	HS-CuO	neat, 110 °C, 1–2 h	60–95	^35^
**9**	MIL-101(Cr)-SB-Cu	DMAP, solvent-free, 100 °C, 6 h	86–95	This work

## Conclusion

In conclusion, an efficient and sustainable protocol for the synthesis of propargylamines and benzofurans via A^3^ coupling and cycloisomerization reaction of aldehydes, amines, and alkynes has been developed utilizing MIL-101(Cr)-SB-Cu as an easily recoverable and reusable heterogeneous catalyst. A series of propargylamine and benzofuran derivatives were synthesized bearing different electron-donating and electron-withdrawing groups. High yields, operational simplicity, and solvent-free reaction condition are some of the advantages of this methodology. The catalyst could be easily separated by centrifugation followed by filtration and shows excellent catalytic activity up to five consecutive runs. The gram-scale synthesis also provided a high yield of 82% implying its possibility for application at the industrial level.

## Experimental section

### General information

All the chemicals required were obtained from Sigma-Aldrich, Alfa Aesar, Spectrochem, and TCI and used without further purification. FT-IR spectra were recorded on a Bruker Alpha II system (ν max in cm^−1^) on KBr disks. ^1^H NMR and ^13^C NMR (400 MHz and 100 MHz respectively) spectra were recorded using a Bruker Avance II-400 spectrometer using CDCl_3_ as the solvent (chemical shifts in δ with TMS as internal standard). Powder XRD analyses were carried out using a Bruker D8 Advance and Rigaku Ultima IV XRD instrument. Transmission Electron Microscopy (TEM) analysis was carried out using a JEOL JSM 100CX system. Scanning electron microscopy (SEM) and Energy Dispersive X-ray (EDX) analysis were carried out using a JSM-6360 (JEOL) system. X-Ray Photoelectron Spectroscopy (XPS) was performed using a PHI 5000 VersaProbe III system. Thermogravimetric analysis (TGA) was carried out using a Perkin Elmer Precisely STA 6000 simultaneous thermal analyzer. Inductively coupled plasma optical emission spectroscopy (ICP-OES) was carried out using Thermo Scientific™ iCAP™ 7600 instrument. TLC Silica gel 60 F_254_ (Merck) was used for TLC analysis. Hexane refers to the fraction boiling between 60 and 80 °C.

### Synthesis of MIL-101(Cr)-NH_2_ (I),^61^ MIL-101(Cr)-SB (II), and MIL-101(Cr)-SB-Cu (III)

A solution of NaOH (0.40 g) in deionized H_2_O (30 mL) was taken in a round bottom flask. To that, 1.60 g of Cr(NO_3_)_3_^.^9H_2_O and 0.72 g of 2-aminobenzene-1,4-dicarboxylic acid (NH_2_-H_2_DBC) were added slowly under constant stirring at room temperature for 30 min. After that, the mixture was transferred into a 50 mL Teflon-lined stainless steel autoclave and kept at 150 °C in a muffle furnace for 12 h. Then, the obtained green powder was collected by filtration and washed thoroughly with H_2_O, DMF, and ethanol respectively. Eventually, the MIL-101(Cr)-NH_2_ was dried at 100 °C. This dried 0.40 g of MIL-101(Cr)-NH_2_ (**I**) was dispersed in 30 mL of ethanol. Then, pyridine-2-carboxaldehyde (0.57 mL) was added dropwise to the mixture and refluxed for 24 h with constant stirring. Finally, the MIL-101(Cr)-SB (**II**) was obtained by centrifugation, filtration and washed properly with ethanol, diethyl ether, and dried. Thereafter, 0.40 g of MIL-101(Cr)-SB (**II**) was dispersed in 30 mL of ethanol. To that mixture, Cu(OAc)_2_·H_2_O (0.25 g) was added and the solution was refluxed for 24 h with constant stirring. After that, the obtained solid materials were centrifuged, filtered, and washed with ethanol, and diethyl ether, and it was dried to get the resulting MIL-101(Cr)-SB-Cu (**III**) complex.

### General procedure for the synthesis of propargylamines

In a 25 mL round bottom flask, aryl aldehydes (**1**, 1 mmol), secondary amines (**2**, 1.2 mmol), and MIL-101(Cr)-SB-Cu (**III**) [15 mg] were taken and stirred at 100 °C under SFRC for 15 min. Then, aryl acetylenes (**3**, 1.5 mmol) were added dropwise to the reaction mixture and stirring was continued for 45 min. Then, 10 mL of ethyl acetate was added to the reaction mixture and the catalyst was separated by centrifugation followed by filtration. The organic solvent was evaporated under reduced pressure and the crude products were further purified by column chromatography (silica gel 100–200 mesh) using ethyl acetate/hexane (1:19) as eluent.

### General procedure for the synthesis of benzofurans

A mixture of substituted 2-hydroxybenzaldehydes (**1**, 1 mmol), secondary amines (**2**, 1.2 mmol), and MIL-101(Cr)-SB-Cu (**III**) [15 mg] was taken in a 25 mL round bottom flask and stirred at 100 °C under SFRC for 15 min. Then, aryl acetylenes (**3**, 1.5 mmol) were added dropwise into the reaction mixture and continued the stirring for another 45 min. Then, DMAP (0.5 mmol) was added to the reaction mixture, and the stirring was continued for 5 h. After that, ethyl acetate (10 mL) was added to the reaction mixture, and the catalyst was separated by centrifugation followed by filtration. The organic solvent was washed with H_2_O (2 × 5 mL), and brine (1 × 5 mL), and dried over anhydrous Na_2_SO_4._ The organic solvent was evaporated under reduced pressure and the crude products were further purified by column chromatography (silica gel 100–200 mesh) using ethyl acetate/hexane as eluent.

### Supplementary Information

Supplementary Information.

## Data Availability

The data used to support this study are included in the article and its supplementary information files.
