# Supplementary material for: A sustainable avenue for the synthesis of propargylamines and benzofurans using a Cu-functionalized MIL-101(Cr) as a reusable heterogeneous catalyst
Source: Sci Rep. 2023 Aug 9;13:12908. doi: 10.1038/s41598-023-40154-0 (PMC10412598; doi:10.1038/s41598-023-40154-0)
Supplement: Supplementary file 1 — Supplementary Information. [file 41598_2023_40154_MOESM1_ESM.pdf]

Supporting Information

A sustainable avenue for the synthesis of propargylamines and benzofurans using a Cu-functionalized MIL-101(Cr) as a reusable heterogeneous catalyst

Fillip Kumar Sarkar,[a] Lenida Kyndiah,[a] Sushmita Gajurel,[a] Rajib Sarkar,[a] Samaresh Jana,[b] and Amarta Kumar Pal[a]*

[a]*Department of Chemistry, Centre for Advanced Studies, North-Eastern Hill University, Shillong-793022, Meghalaya, India.*

[b]*Department of Chemistry, School of Applied Sciences, KIIT- Deemed to be University, Bhubaneswar-751024, Odisha, India.*

E-mail: amartya_pal22@yahoo.com, akpal@nehu.ac.in Tel: +91 364 2722606

**Table of content**	**Page No.**
1. Spectral data of the compounds (**4a-m and 5a-k**)	S2 – S10
2. References	S10
3. ^{1}H and ^{13}C NMR spectra of compounds (**4a-m and 5a-k**)	S11 – S34

1. Spectral data of the compounds (4a-m and 5a-k).

1. **4-(1,3-diphenylprop-2-yn-1-yl)morpholine (4a).**[1]

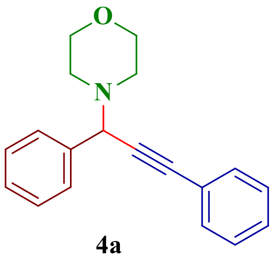

4a

Light yellow liquid. **^{1}H NMR** ($CDCl_3$, 400 MHz): δ = 7.64-7.61 (m, 2H), 7.53-7.49 (m, 2H), 7.39-7.28 (m, 6H), 4.79 (s, 1H), 3.77-3.68 (m, 4H), 2.66-2.60 (m, 4H). **^{13}C NMR** ($CDCl_3$, 100 MHz): δ = 137.8, 131.9, 128.7, 128.4, 128.38, 128.35, 127.9, 123.0, 88.5, 85.1, 67.2, 62.1, 49.9.

2. **4-(3-phenyl-1-(*p*-tolyl)prop-2-yn-1-yl)morpholine (4b).**[1]

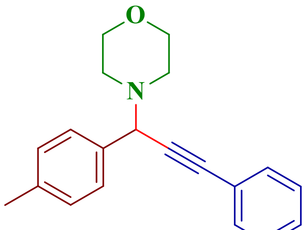

4b

Yellow gummy. **^{1}H NMR** ($CDCl_3$, 400 MHz): δ = 7.51-7.49 (m, 4H), 7.33-7.30 (m, 3H), 7.18 (d, *J* = 7.6 Hz, 2H), 4.75 (s, 1H), 3.73 (s, 4H), 2.63 (s, 4H), 2.35 (s, 3H). **^{13}C NMR** ($CDCl_3$, 100 MHz): δ = 137.5, 134.7, 131.8, 128.9, 128.5, 128.3, 128.2, 123.0, 88.2, 85.3, 67.1, 61.8, 49.9, 21.1.

3. **4-(1-(4-methoxyphenyl)-3-phenylprop-2-yn-1-yl)morpholine (4c).**[2]

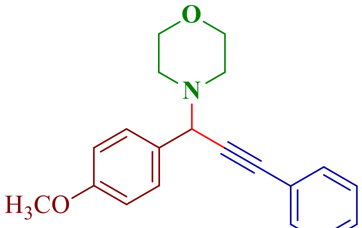

4c

White solid. **^{1}H NMR** ($CDCl_3$, 400 MHz): δ = 7.54-7.49 (m, 4H), 7.34-7.30 (m, 3H), 6.91-6.87 (m, 2H), 4.72 (s, 1H), 3.80 (s, 3H), 3.75-3.70 (m, 4H), 2.62-2.59 (m, 4H). **^{13}C NMR** ($CDCl_3$, 100 MHz): δ = 159.2, 131.8, 129.9, 129.8, 128.4, 128.3, 123.1, 113.6, 88.3, 85.4, 67.2, 61.5, 55.3, 49.9.

4. **2-(1-morpholino-3-phenylprop-2-yn-1-yl)phenol (4d).**[1]

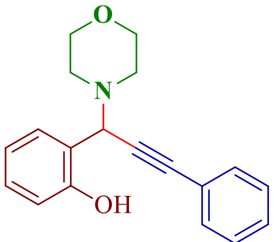

4d

White solid. **^{1}H NMR** ($CDCl_3$, 400 MHz): δ = 10.79 (brs, 1H), 7.56-7.52 (m, 3H), 7.37-7.34 (m, 3H), 7.25-7.21 (m, 1H), 6.89-6.85 (m, 2H), 5.08 (s, 1H), 3.79 (s, 4H), 2.78 (s, 4H). **^{13}C NMR** ($CDCl_3$, 100 MHz): δ = 157.1, 131.9, 129.8, 128.8, 128.5, 122.3, 120.6, 119.5, 116.6, 90.5, 81.6, 66.9, 60.8, 48.9.

5. **4-(1-(4-chlorophenyl)-3-phenylprop-2-yn-1-yl)morpholine (4e).**[1]

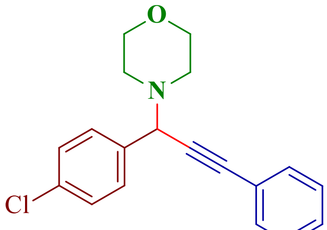

4e

Yellow gummy. **^{1}H NMR** ($CDCl_3$, 400 MHz): δ = 7.59-7.55 (m, 2H), 7.51-7.48 (m, 2H), 7.34-7.31 (m, 5H), 4.76 (s, 1H), 3.77-3.69 (m, 4H), 2.62 (t, J = 4.4 Hz, 4H). **^{13}C NMR** ($CDCl_3$, 100 MHz): δ = 136.4, 133.6, 131.9, 130.0, 128.5, 128.49, 128.47, 122.7, 89.0, 84.3, 67.1, 61.4, 49.8.

6. **4-(1-(4-fluorophenyl)-3-phenylprop-2-yn-1-yl)morpholine (4f).**[3]

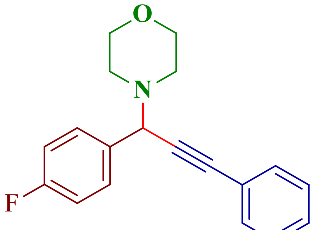

4f

Yellow gummy. **^{1}H NMR** ($CDCl_3$, 400 MHz): δ = 7.62-7.58 (m, 2H), 7.51-7.49 (m, 2H), 7.34-7.31 (m, 3H), 7.07-7.01 (m, 2H), 4.78 (s, 1H), 3.78-3.69 (m, 4H), 2.63 (t, J = 4.4 Hz, 4H). **^{13}C NMR** ($CDCl_3$, 100 MHz): δ = 163.7, 161.2, 133.48, 133.45, 131.9, 130.3, 130.2, 128.5, 128.4, 122.8, 115.2, 115.0, 88.93, 84.6, 67.1, 61.3, 49.8.

7. **4-(1-(4-bromophenyl)-3-phenylprop-2-yn-1-yl)morpholine (4g).**[3]

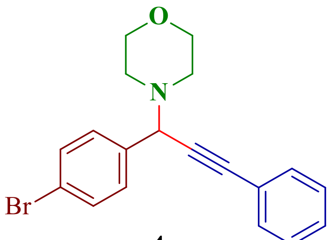

4g

White solid. **^{1}H NMR** ($CDCl_3$, 400 MHz): δ = 7.52-7.46 (m, 6H), 7.34-7.32 (m, 3H), 4.73 (s, 1H), 3.76-3.68 (m, 4H), 2.61 (t, *J* = 4.2 Hz, 4H). **^{13}C NMR** ($CDCl_3$, 100 MHz): δ = 137.1, 131.9, 131.4, 130.3, 128.5, 128.4, 122.7, 121.8, 89.0, 84.3, 67.2, 61.5, 49.8.

8. **4-(1-(3-chlorophenyl)-3-phenylprop-2-yn-1-yl)morpholine (4h)**.[4]

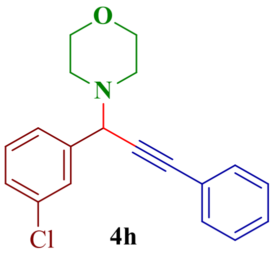

4h

Yellow gummy. **^{1}H NMR** ($CDCl_3$, 400 MHz): δ = 7.67 (s, 1H), 7.56-7.52 (m, 3H), 7.35-7.32 (m, 3H), 7.30-7.28 (m, 2H), 4.78 (s, 1H), 3.79-3.70 (m, 4H), 2.64 (t, *J* = 4.4 Hz, 4H). **^{13}C NMR** ($CDCl_3$, 100 MHz): δ = 140.1, 134.3, 131.9, 129.6, 128.7, 128.6, 128.5, 128.1, 126.8, 122.7, 89.2, 84.1, 67.1, 61.6, 49.9.

9. **4-(1-(3-bromophenyl)-3-phenylprop-2-yn-1-yl)morpholine (4i)**.[5]

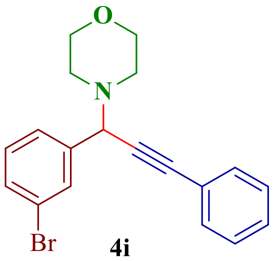

4i

Yellow gummy. **^{1}H NMR** ($CDCl_3$, 400 MHz): δ = 7.57 (s, 1H), 7.48-7.43 (m, 3H), 7.27-7.21 (m, 5H), 4.71 (s, 1H), 3.71-3.64 (m, 4H), 2.56 (s, 4H). **^{13}C NMR** ($CDCl_3$, 100 MHz): δ = 139.9, 134.3, 131.9, 129.6, 128.7, 128.6, 128.5, 128.1, 126.8, 122.7, 89.2, 84.0, 67.1, 61.6, 49.8.

10. **4-(1-(2-chlorophenyl)-3-phenylprop-2-yn-1-yl)morpholine (4j)**.[2]

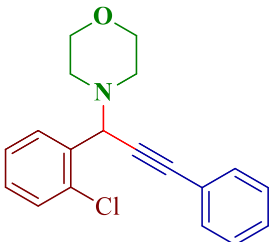

4j

Yellow gummy. **^{1}H NMR** ($CDCl_3$, 400 MHz): δ = 7.68 (d, *J* = 6.8 Hz, 1H), 7.53 (d, *J* = 8 Hz, 1H), 7.43-7.41 (m, 2H), 7.26-7.24 (m, 4H), 7.12 (t, *J* = 7.6 Hz, 1H), 4.99 (s, 1H), 3.62 (s, 4H), 2.60 (s, 4H). **^{13}C NMR** ($CDCl_3$, 100 MHz): δ = 137.1, 133.3, 131.8, 130.7, 129.4, 128.4, 128.3, 126.9, 125.2, 122.7, 88.6, 84.5, 67.1, 61.3, 49.7.

11. **4-(1-(3-bromophenyl)-3-phenylprop-2-yn-1-yl)morpholine (4k).**[4]

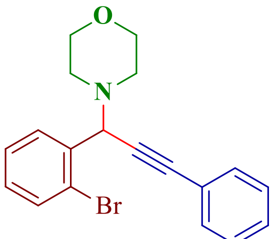

4k

Light yellow gummy. **^{1}H NMR** ($CDCl_3$, 400 MHz): δ = 7.76 (dd, *J* = 1.8, 7.8 Hz, 1H), 7.60 (dd, *J* = 1.4, 7.8 Hz, 1H), 7.51-7.48 (m, 2H), 7.34-7.29 (m, 4H), 7.19-7.14 (m, 1H), 5.07 (s, 1H), 3.75-3.64 (m, 4H), 2.71-2.62 (m, 4H). **^{13}C NMR** ($CDCl_3$, 100 MHz): δ = 137.2, 133.3, 131.9, 130.7, 129.4, 128.49, 128.45, 127.0, 125.3, 122.8, 88.7, 84.6, 67.2, 61.3, 49.7.

12. **4-(1-(furan-2-yl)-3-phenylprop-2-yn-1-yl)morpholine (4l)**[4]

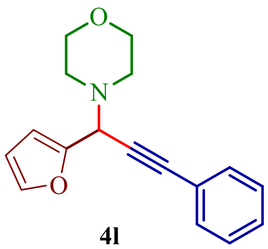

4l

Brown gummy. **^{1}H NMR** ($CDCl_3$, 400 MHz): δ = 7.43-7.42 (m, 2H), 7.37 (s, 1H), 7.27-7.26 (m, 3H), 6.47 (s, 1H), 6.30 (s, 1H), 4.85 (s, 1H), 3.72 (s, 4H), 2.62 (s, 4H). **^{13}C NMR** ($CDCl_3$, 100 MHz): δ = 150.5, 142.9, 131.8, 128.5, 128.3, 122.4, 110.1, 109.9, 87.1, 82.6, 66.8, 56.0, 49.5.

13. **(*E*)-4-(1,5-diphenylpent-1-en-4-yn-3-yl)morpholine (4m)**

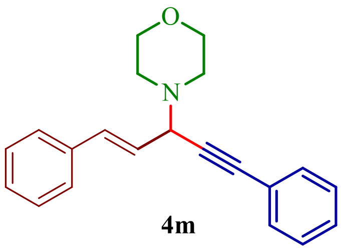

4m

Light yellow gummy. ^{1}**H NMR** ($CDCl_3$, 400 MHz): δ = 7.44 (s, 2H), 7.37-7.35 (m, 2H), 7.27-7.24 (m, 5H), 7.20-7.17 (m, 1H), 6.85 (d, *J* = 16.0 Hz, 1H), 6.27 (dd, *J* = 5, 15.8 Hz, 1H), 4.34 (s, 1H), 3.72 (s, 4H), 2.74-2.62 (m, 4H). ^{13}**C NMR** ($CDCl_3$, 100 MHz): δ = 136.6, 133.7, 131.8, 128.6, 128.4, 128.3, 127.9, 126.7, 126.2, 122.7, 88.7, 84.0, 67.0, 60.0, 49.9.

14. **4-(2-benzylbenzofuran-3-yl)morpholine (5a)**.[1]

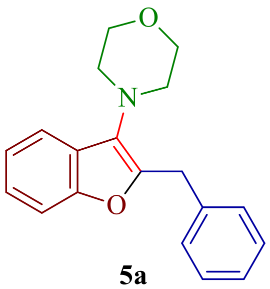

5a

Light yellow solid. ^{1}**H NMR** ($CDCl_3$, 400 MHz): δ = 7.67-7.65 (m, 1H), 7.38-7.36 (m, 1H), 7.30-7.24 (m, 4H), 7.22-7.14 (m, 3H), 4.16 (s, 2H), 3.85 (t, *J* = 4.6 Hz, 4H), 3.18 (t, *J* = 4.4 Hz, 4H). ^{13}**C NMR** ($CDCl_3$, 100 MHz): δ = 153.5, 150.2, 138.1, 128.7, 128.57, 128.55, 126.4, 126.0, 123.4, 122.1, 119.9, 111.7, 67.7, 52.6, 32.2.

15. **4-(2-benzyl-6-methoxybenzofuran-3-yl)morpholine (5b)**.

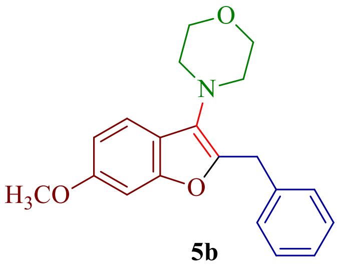

5b

White solid. ^{1}**H NMR** ($CDCl_3$, 400 MHz): δ = 7.52 (d, *J* = 8.4 Hz, 1H), 7.31-7.18 (m, 5H), 6.92 (d, *J* = 2.4 Hz, 1H), 6.82 (dd, *J* = 2.2, 8.6 Hz, 1H), 4.13 (s, 2H), 3.85 (t, *J* = 4.6 Hz, 4H), 3.80 (s, 3H), 3.16 (t, *J* = 4.6 Hz, 4H). ^{13}**C NMR** ($CDCl_3$, 100 MHz): δ = 157.4, 154.5, 148.9, 138.5, 128.7, 128.6, 128.5, 126.5, 120.1, 119.4, 110.9, 96.5, 67.7, 55.7, 52.7, 32.3.

16. **4-(2-benzyl-7-methoxybenzofuran-3-yl)morpholine (5c)**.[6]

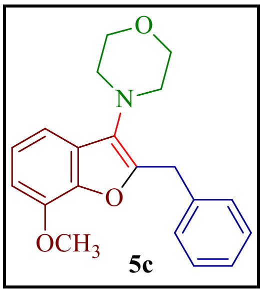

White solid. **^{1}H NMR** ($CDCl_3$, 400 MHz): δ = 7.26-7.24 (m, 5H), 7.21-7.15 (m, 1H), 7.11 (t, *J* = 7.8 Hz, 1H), 6.74 (dd, *J* = 0.8, 8.0 Hz, 1H), 4.17 (s, 2H), 3.96 (s, 3H), 3.82 (t, *J* = 4.6 Hz, 4H), 3.13 (t, *J* = 4.6 Hz, 4H). **^{13}C NMR** ($CDCl_3$, 100 MHz): δ = 150.5, 145.5, 142.7, 138.3, 129.1, 128.6, 128.5, 127.9, 126.4, 122.8, 112.4, 105.8, 67.7, 56.0, 52.5, 32.2.

17. **4-(2-benzyl-5-bromobenzofuran-3-yl)morpholine (5d).**[6]

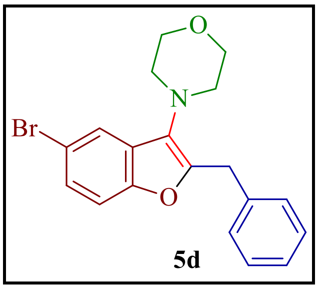

Off white solid. **^{1}H NMR** ($CDCl_3$, 400 MHz): δ = 7.77 (s, 1H), 7.31-7.27 (m, 3H), 7.24-7.19 (m, 4H), 4.14 (s, 2H), 3.84 (t, *J* = 4.6 Hz, 4H), 3.13 (t, *J* = 4.6 Hz, 4H). **^{13}C NMR** ($CDCl_3$, 100 MHz): δ = 152.3, 151.8, 137.8, 128.7, 128.6, 128.3, 128.1, 126.7, 126.4, 122.5, 115.3, 113.2, 67.6, 52.5, 32.4.

18. **4-(2-benzyl-5-chlorobenzofuran-3-yl)morpholine (5e).**[6]

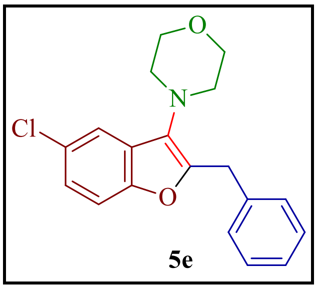

Orange solid. **^{1}H NMR** ($CDCl_3$, 400 MHz): δ = 7.62 (d, *J* = 2 Hz, 1H), 7.29-7.21 (m, 6H), 7.16 (dd, *J* = 2.2, 8.6 Hz, 1H), 4.14 (s, 2H), 3.84 (t, *J* = 4.6 Hz, 4H), 3.13 (t, *J* = 4.6 Hz, 4H). **^{13}C NMR** ($CDCl_3$, 100 MHz): δ = 151.98, 151.92, 137.8, 128.7, 128.6, 128.5, 127.7, 127.4, 126.7, 123.7, 119.5, 112.7, 67.7, 52.5, 32.4.

19. **4-(2-benzyl-5-nitrobenzofuran-3-yl)morpholine (5f).**[1]

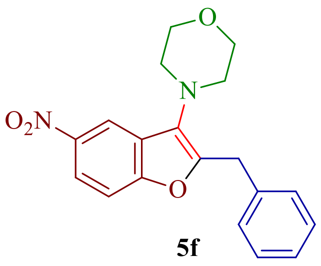

5f

Yellow solid. ^{1}**H NMR** ($CDCl_3$, 400 MHz): δ = 8.54 (d, *J* = 2.4 Hz, 1H), 8.14 (dd, *J* = 2.4, 9.2 Hz, 1H), 7.43 (d, *J* = 9.2 Hz, 1H), 7.33-7.29 (m, 2H), 7.26-7.21 (m, 3H), 4.18 (s, 2H), 3.87 (t, *J* = 4.6 Hz, 4H), 3.18 (t, *J* = 4.6 Hz, 4H). ^{13}**C NMR** ($CDCl_3$, 100 MHz): δ = 156.3, 153.5, 143.5, 137.2, 129.5, 128.8, 128.6, 126.9, 126.5, 119.6, 116.3, 112.0, 67.6, 52.5, 32.6.

20. **4-(2-benzyl-5,7-dichlorobenzofuran-3-yl)morpholine (5g)**.

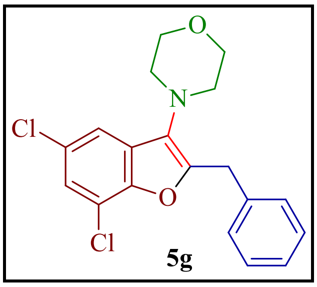

5g

Off white solid. ^{1}**H NMR** ($CDCl_3$, 400 MHz): δ = 7.51-7.50 (m, 1H), 7.32-7.20 (m, 6H), 4.17 (s, 2H), 3.82 (t, *J* = 4.4 Hz, 4H), 3.09 (t, *J* = 4.6 Hz, 4H). ^{13}**C NMR** ($CDCl_3$, 100 MHz): δ = 153.0, 147.8, 137.4, 128.9, 128.7, 128.67, 128.63, 128.0, 126.8, 123.7, 118.1, 117.7, 67.6, 52.3, 32.5.

21. **4-(2-(4-methylbenzyl)benzofuran-3-yl)morpholine (5h).**[1]

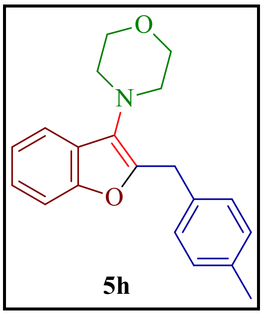

5h

Yellow gummy. ^{1}**H NMR** ($CDCl_3$, 400 MHz): δ = 7.76-7.74 (m, 1H), 7.47-7.45 (m, 1H), 7.30-7.25 (m, 4H), 7.20-7.18 (m, 2H), 4.22 (s, 2H), 3.94 (t, *J* = 4.6 Hz, 4H), 3.27 (t, *J* = 4.4 Hz, 4H), 2.39 (s, 3H). ^{13}**C NMR** ($CDCl_3$, 100 MHz): δ = 153.6, 150.6, 136.1, 135.3, 129.4, 128.8, 128.6, 126.3, 123.6, 122.2, 120.0, 111.9, 67.8, 52.8, 32.0, 21.2.

22. **4-(6-methoxy-2-(4-methylbenzyl)benzofuran-3-yl)morpholine (5i)**.

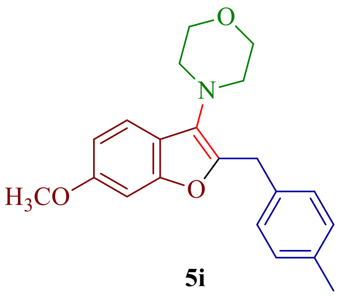

5i

Yellow gummy. ^{1}**H NMR** ($CDCl_3$, 400 MHz): δ = 7.44 (d, *J* = 8.8 Hz, 1H), 7.09 (d, *J* = 8 Hz, 2H), 7.02 (d, *J* = 8 Hz, 2H), 6.85 (d, *J* = 2.4 Hz, 1H), 6.75 (dd, *J* = 2.4, 8.4 Hz, 1H), 4.06 (s, 2H), 3.80 (s, 4H), 3.73 (s, 3H), 3.11 (s, 4H), 2.23 (s, 3H). ^{13}**C NMR** ($CDCl_3$, 100 MHz): δ = 157.3, 154.5, 149.0, 135.9, 135.3, 129.2, 128.3, 119.9, 110.9, 96.5, 67.6, 55.7, 52.7, 31.8, 21.0.

23. **4-(7-methoxy-2-(4-methylbenzyl)benzofuran-3-yl)morpholine (5j)**.

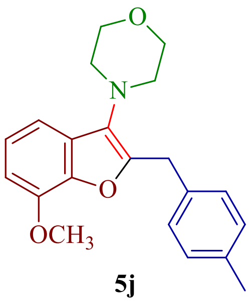

5j

White solid. ^{1}**H NMR** ($CDCl_3$, 400 MHz): δ = 7.25-7.23 (m, 1H), 7.16 (d, *J* = 8 Hz, 2H), 7.11-7.05 (m, 3H), 6.73 (d, *J* = 8.4 Hz, 1H), 4.13 (s, 2H), 3.95 (s, 3H), 3.83 (t, *J* = 4.6 Hz, 4H), 3.14 (t, *J* = 4.6 Hz, 4H), 2.29 (s, 3H). ^{13}**C NMR** ($CDCl_3$, 100 MHz): δ = 150.7, 145.5, 142.7, 135.9, 135.2, 129.2, 129.0, 128.4, 127.9, 122.8, 112.4, 105.8, 67.7, 56.0, 52.5, 31.9, 21.1.

24. **4-(2-(4-methylbenzyl)-5-nitrobenzofuran-3-yl)morpholine (5k)**.[1]

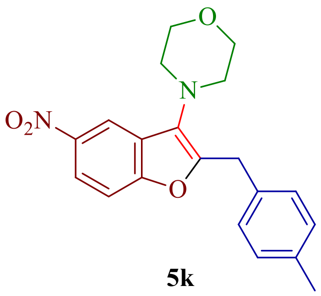

5k

Light yellow solid. ^{1}**H NMR** ($CDCl_3$, 400 MHz): δ = 8.53 (d, *J* = 2.4 Hz, 1H), 8.14 (dd, *J* = 2.4, 9.2 Hz, 1H), 7.42 (d, *J* = 9.2 Hz, 1H), 7.15-7.10 (m, 4H), 4.14 (s, 2H), 3.88 (t, *J* = 4.6 Hz,

4H), 3.19 (t, *J* = 4.6 Hz, 4H), 2.31 (s, 3H). **^{13}C NMR** ($CDCl_3$, 100 MHz): δ = 156.3, 153.8, 143.5, 136.3, 134.1, 129.5, 129.3, 128.5, 126.6, 119.6, 116.3, 112.0, 67.6, 52.5, 32.2, 21.1.

2. Reference

(1) Z. Xu, J. Xu and Y.Li, *Appl Organomet Chem.*, 2021, **35**, e6349.

(2) S. Ostovar, A. Rezvani, R. Luque, and C. Carrillo-Carrión. *Mol. Catal.*, 2020, **493**, 111042.

(3) A. Mittal, S. Kumari, Parmanand, D. Yadav, and S. K. Sharma, *Appl Organomet Chem.*, 2020, **34**, e5362.

(4) P. Kaur, B. Kumar, V. Kumar, and R. Kumar, *Tetrahedron Lett.*, 2018, **59**, 1986-1991.

(5) M. Milen, G. Györke, A. Dancsó, B. Volk, *Tetrahedron Lett.*, 2020, **61**, 151544.

(6) B. Abtahi and H. Tavakol, *Appl Organomet Chem.*, 2021, **35**, e6433.

3. ^{1}H and ^{13}C NMR spectra of compounds (4a-m and 5a-k).

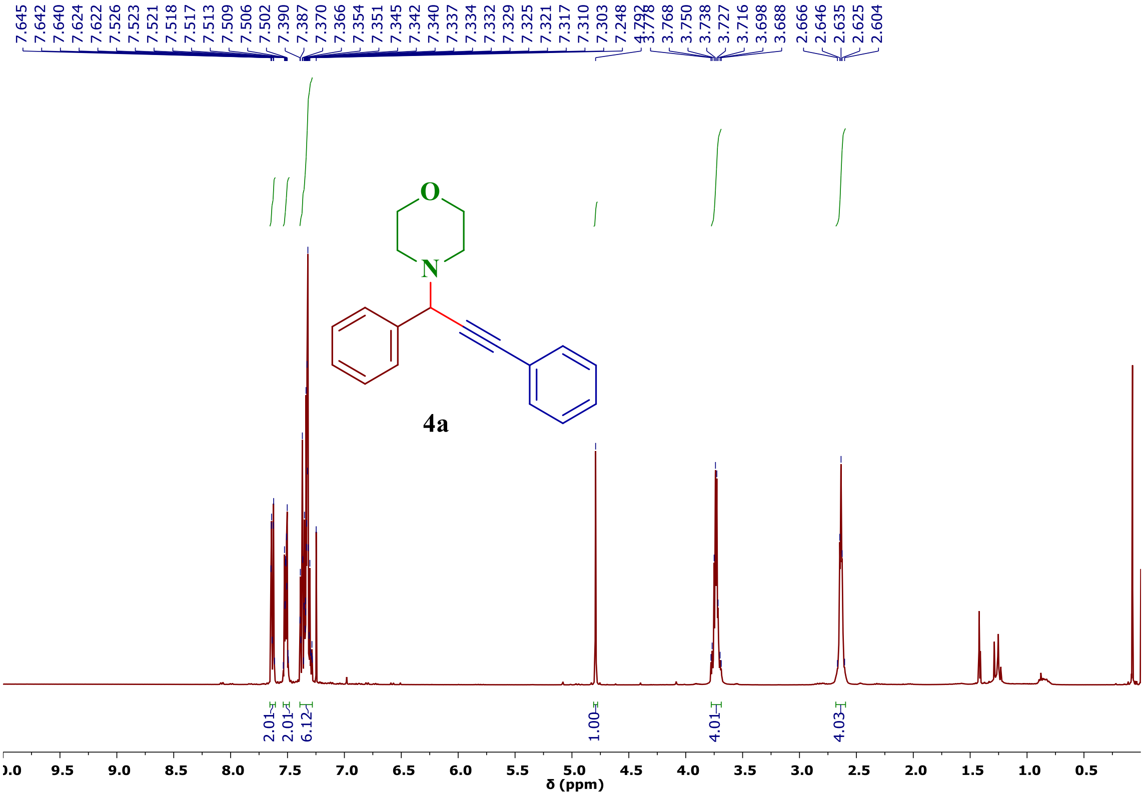

Figure S1. ^{1}H NMR Spectrum of **4a** in $CDCl_3$.

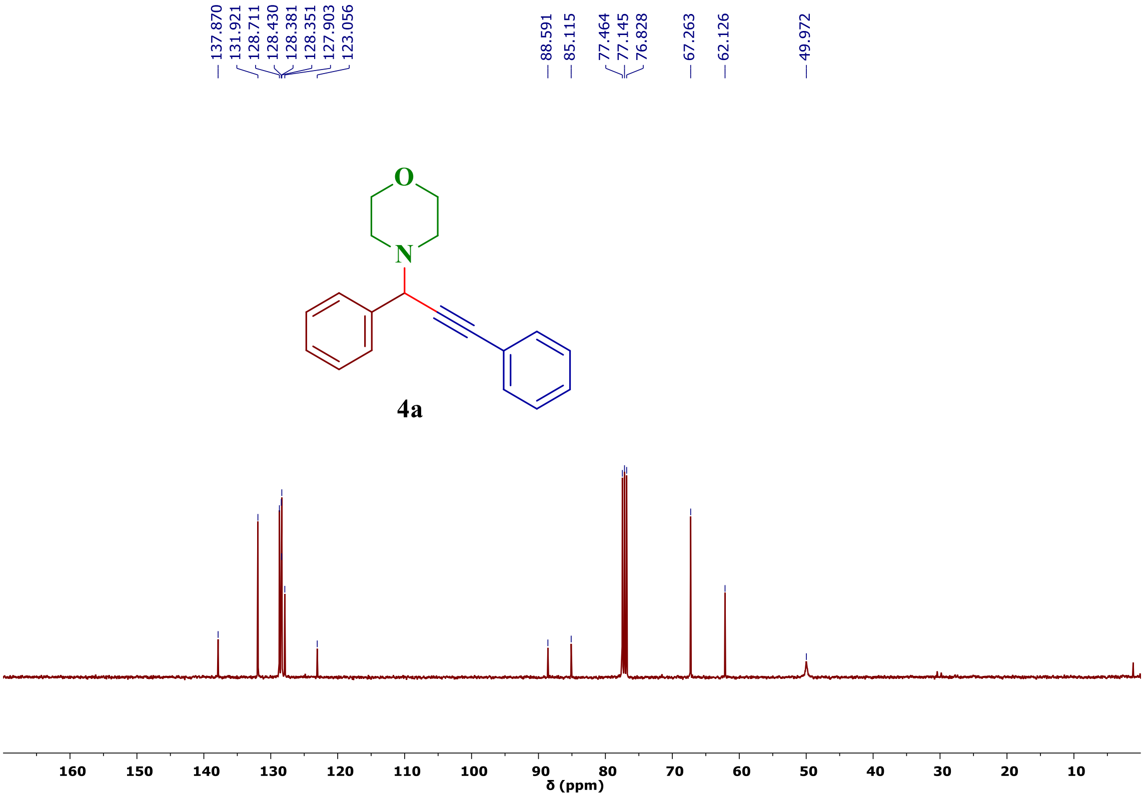

Figure S2. ^{13}C NMR Spectrum of **4a** in $CDCl_3$.

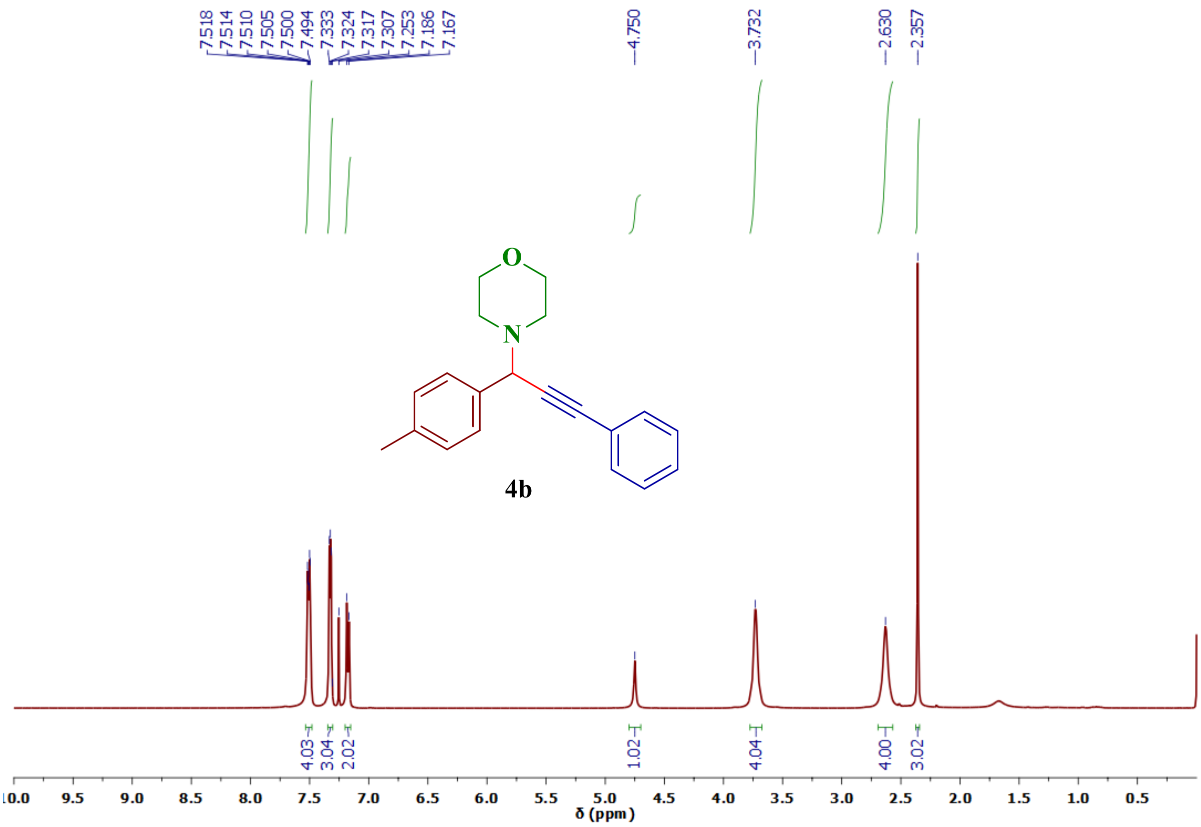

Figure S3. ^{1}H NMR Spectrum of **4b** in $CDCl_3$.

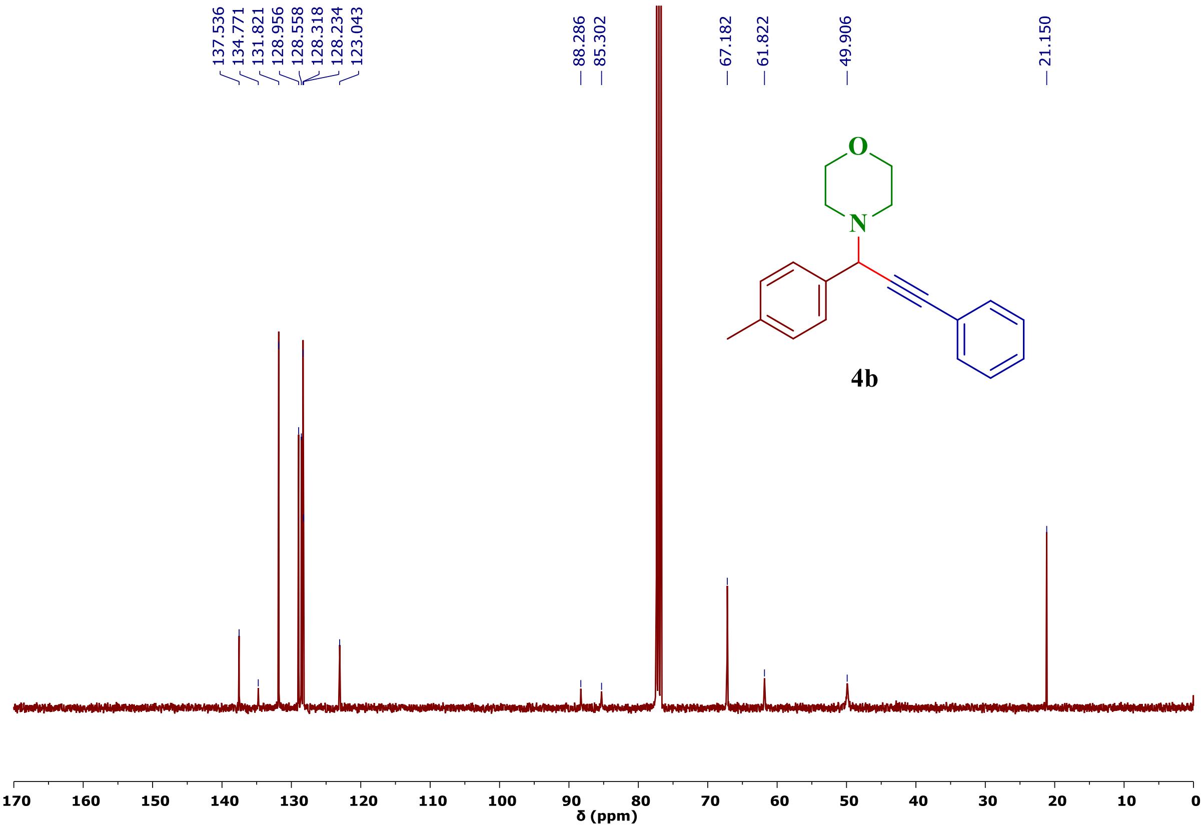

Figure S4. ^{13}C NMR Spectrum of **4b** in $CDCl_3$.

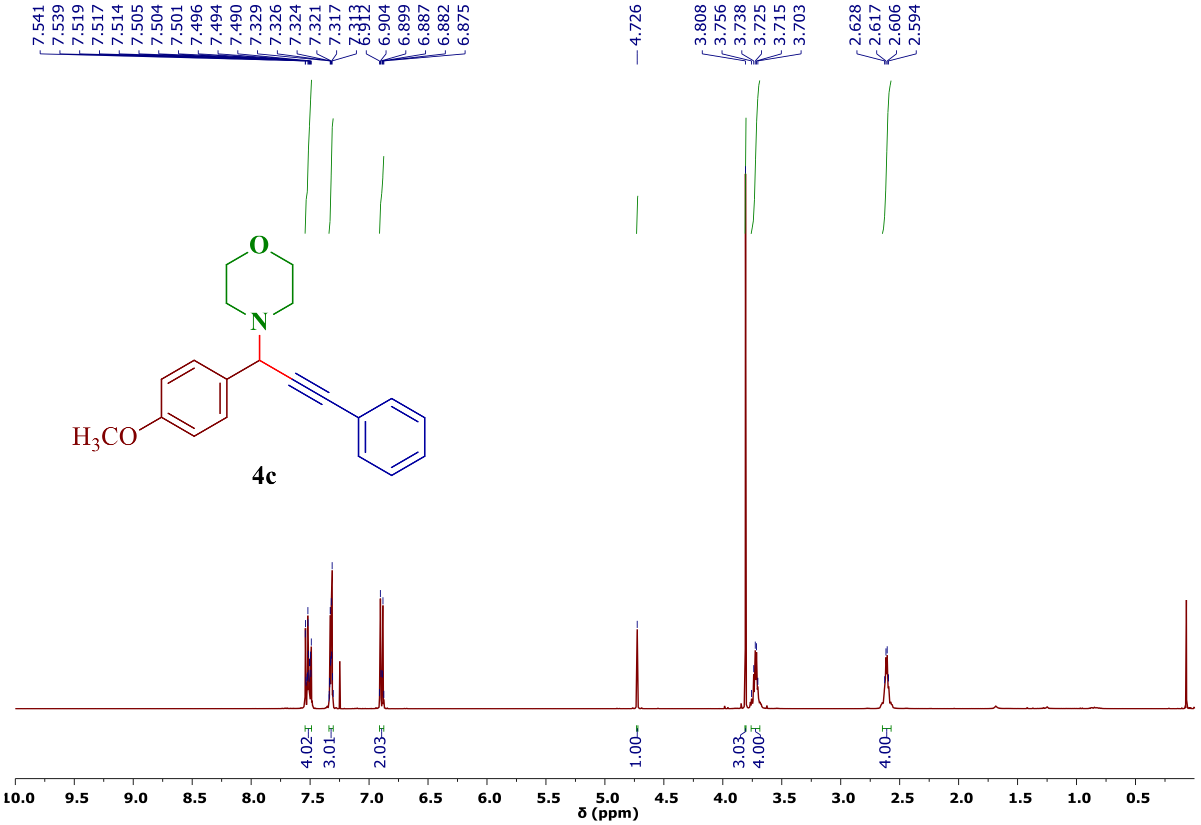

Figure S5. ^{1}H NMR Spectrum of **4c** in $CDCl_3$.

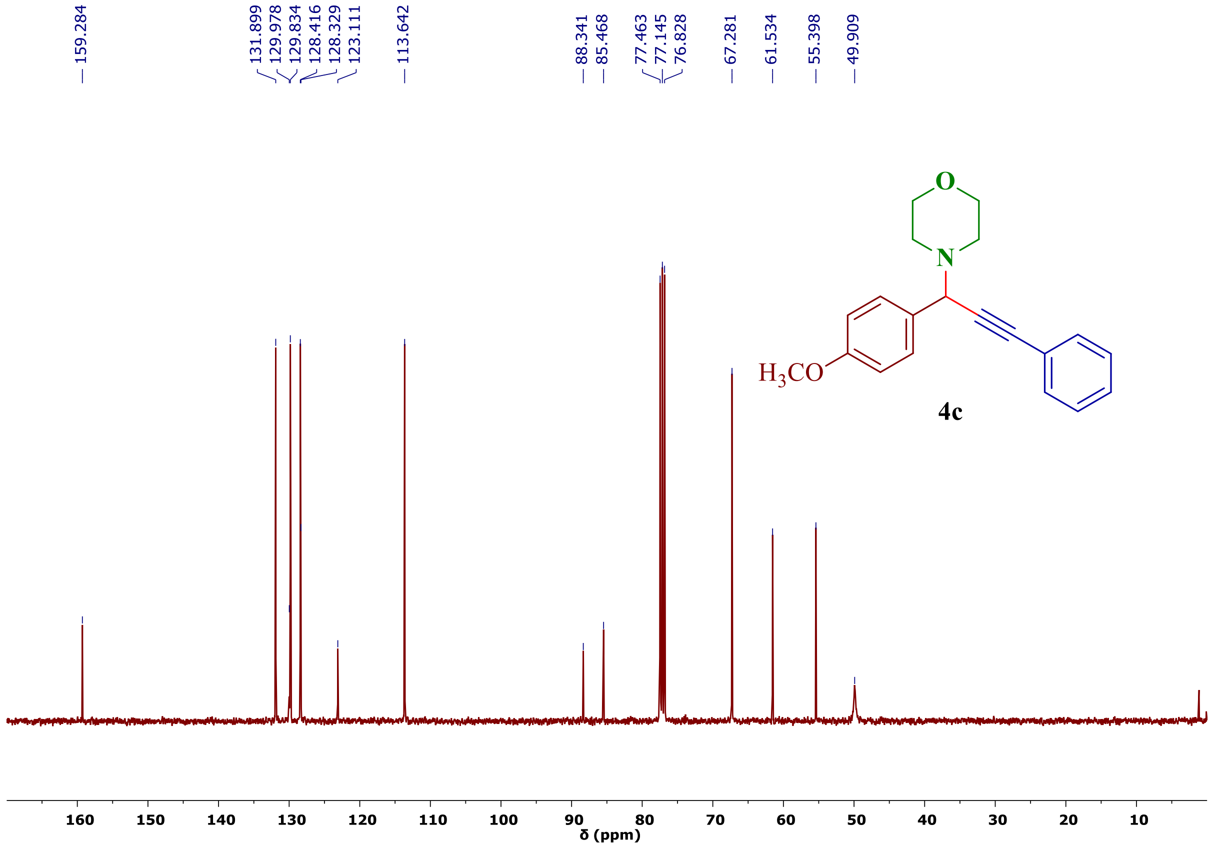

Figure S6. ^{13}C NMR Spectrum of **4c** in $CDCl_3$.

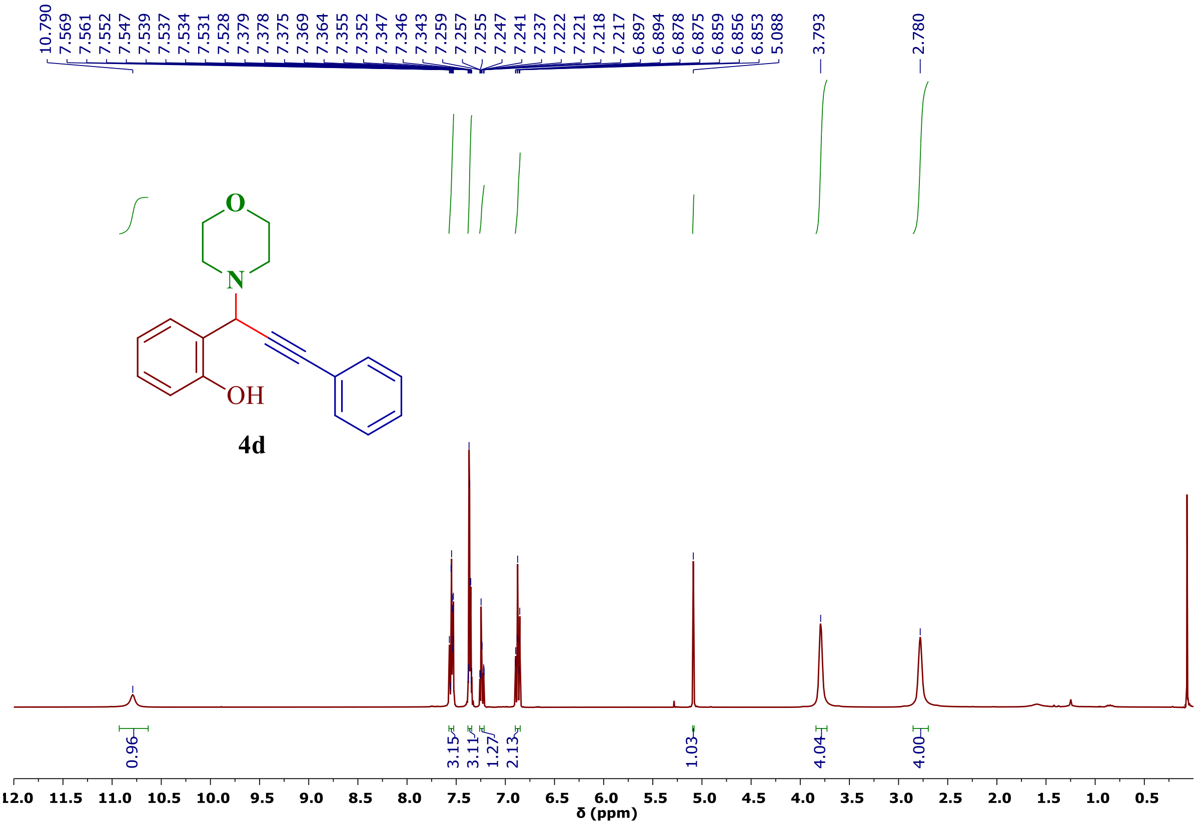

Figure S7. ^{1}H NMR Spectrum of **4d** in $CDCl_3$.

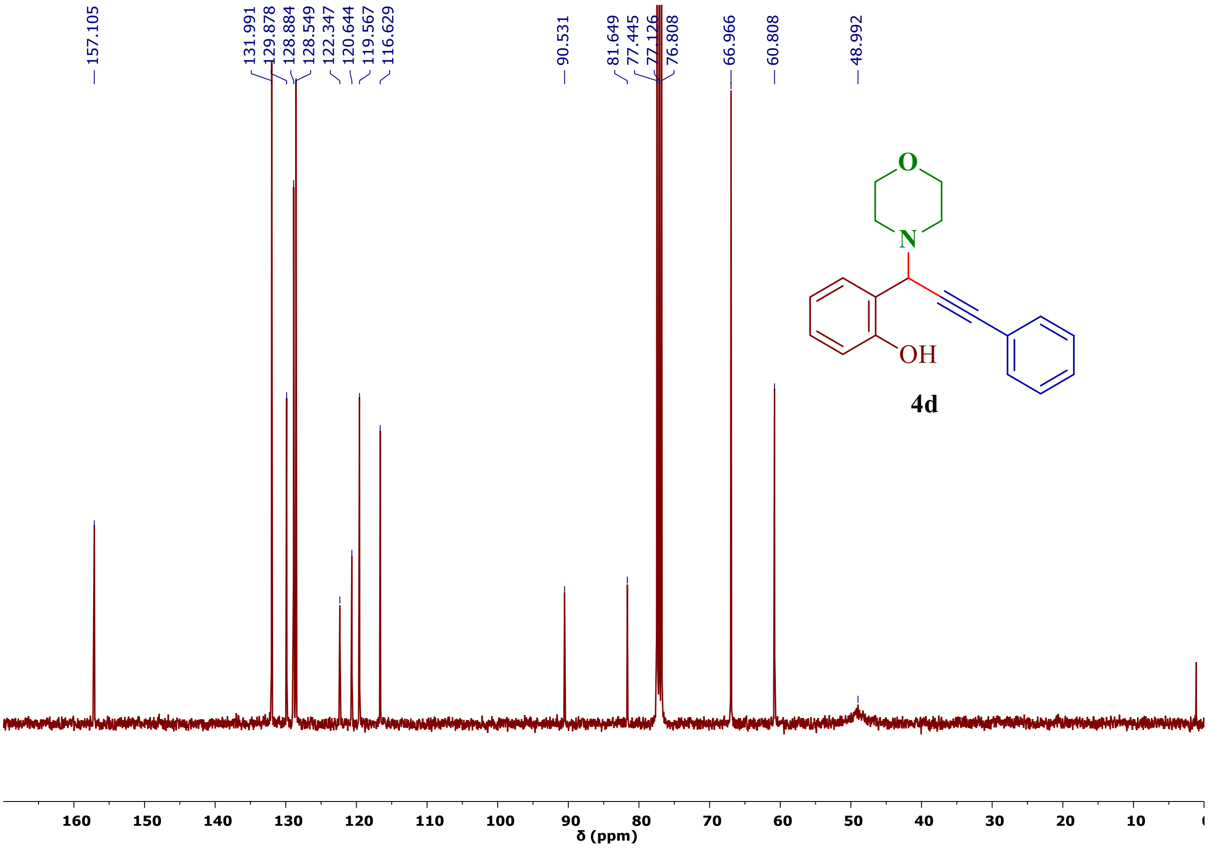

Figure S8. ^{13}C NMR Spectrum of **4d** in $CDCl_3$.

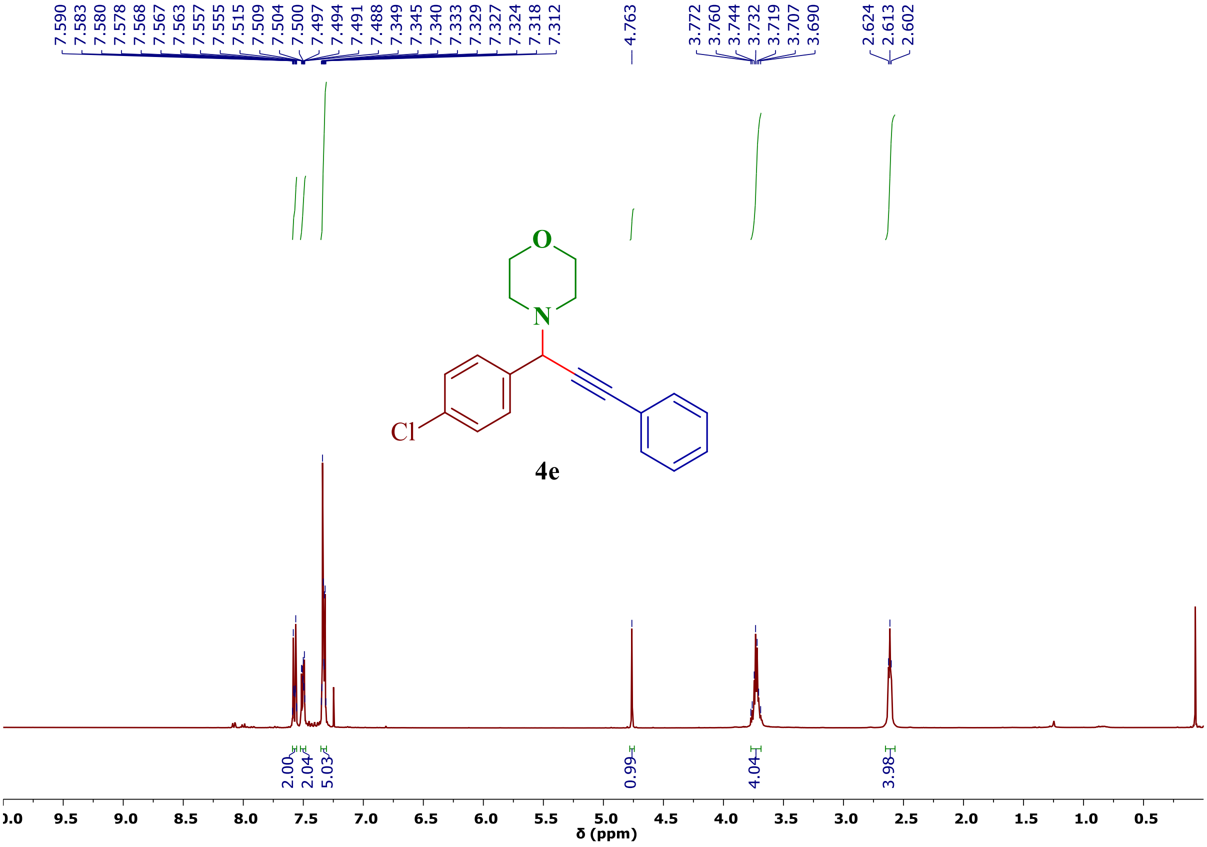

Figure S9. ^{1}H NMR Spectrum of **4e** in $CDCl_3$.

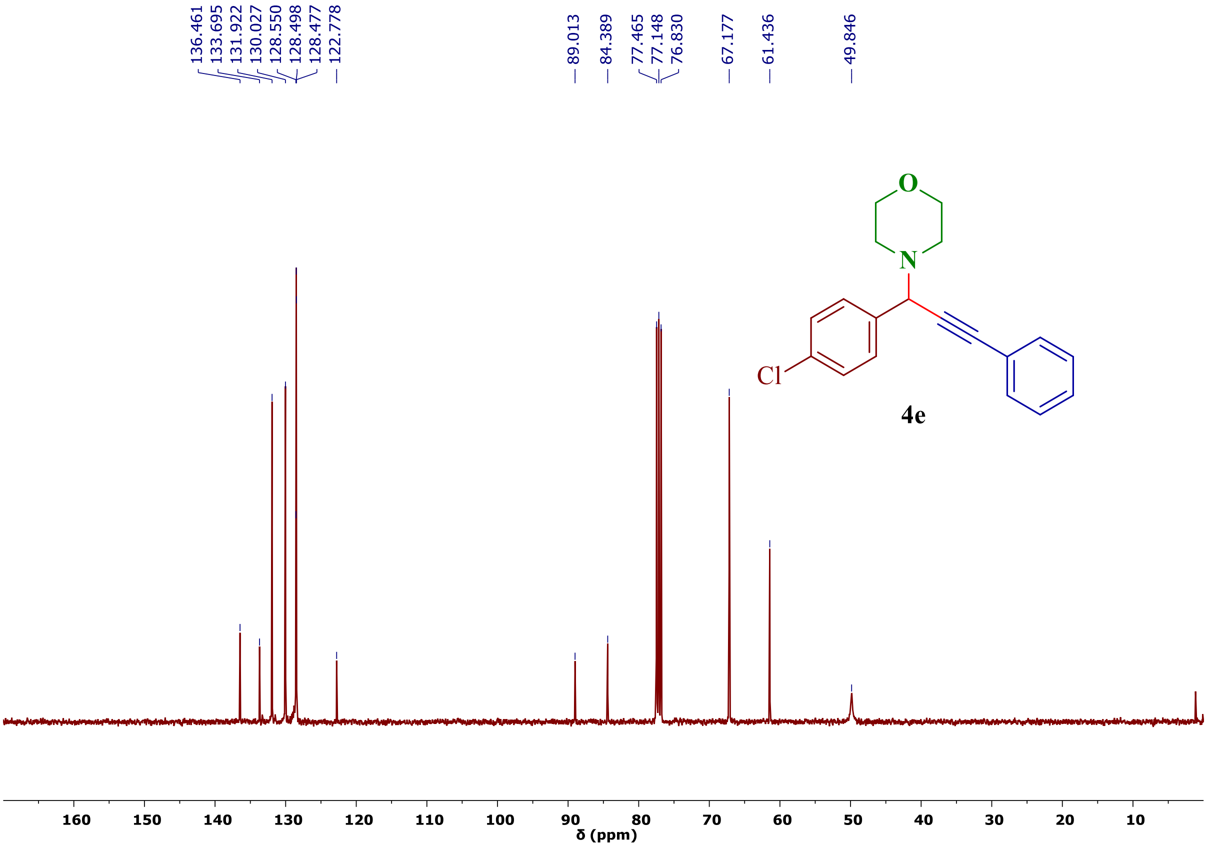

Figure S10. ^{13}C NMR Spectrum of **4e** in $CDCl_3$.

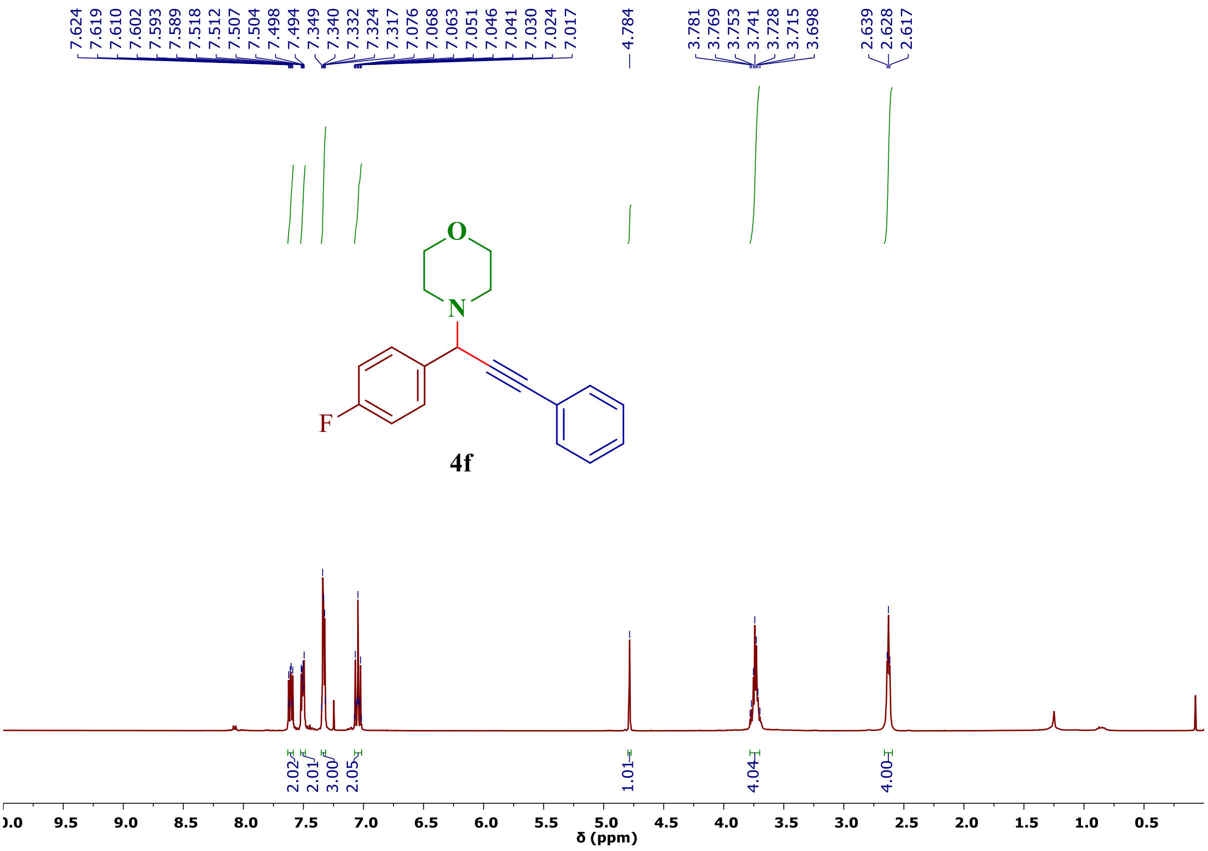

Figure S11. ^{1}H NMR Spectrum of **4f** in $CDCl_3$.

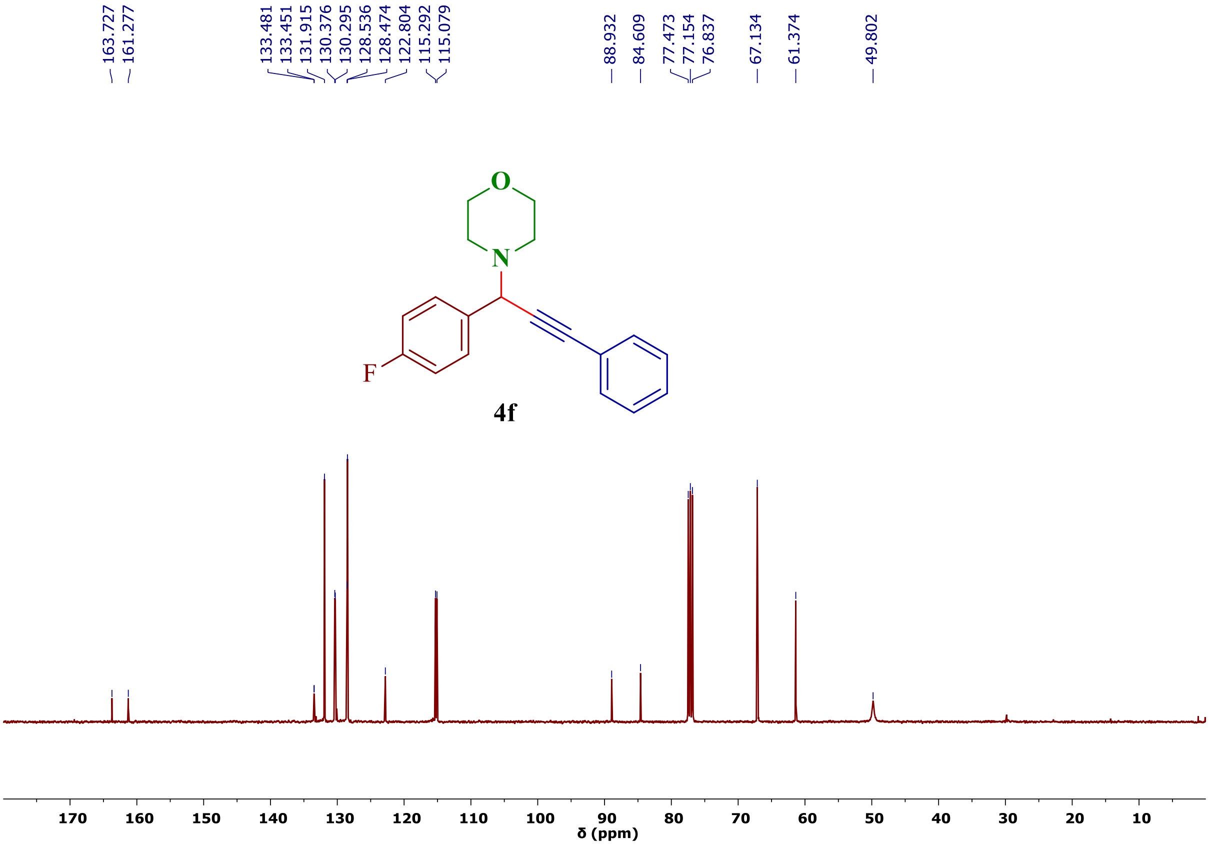

Figure S12. ^{13}C NMR Spectrum of **4f** in $CDCl_3$.

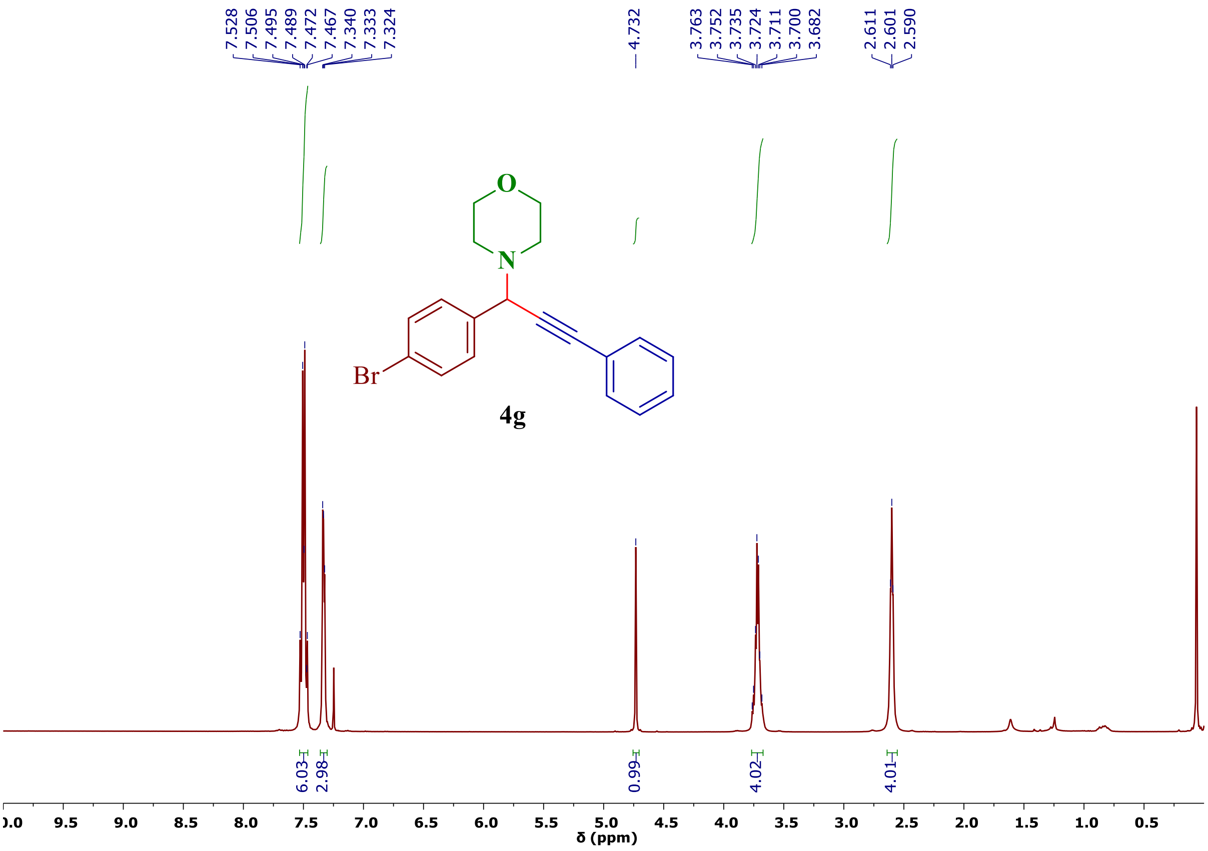

Figure S13. ^{1}H NMR Spectrum of **4g** in $CDCl_3$.

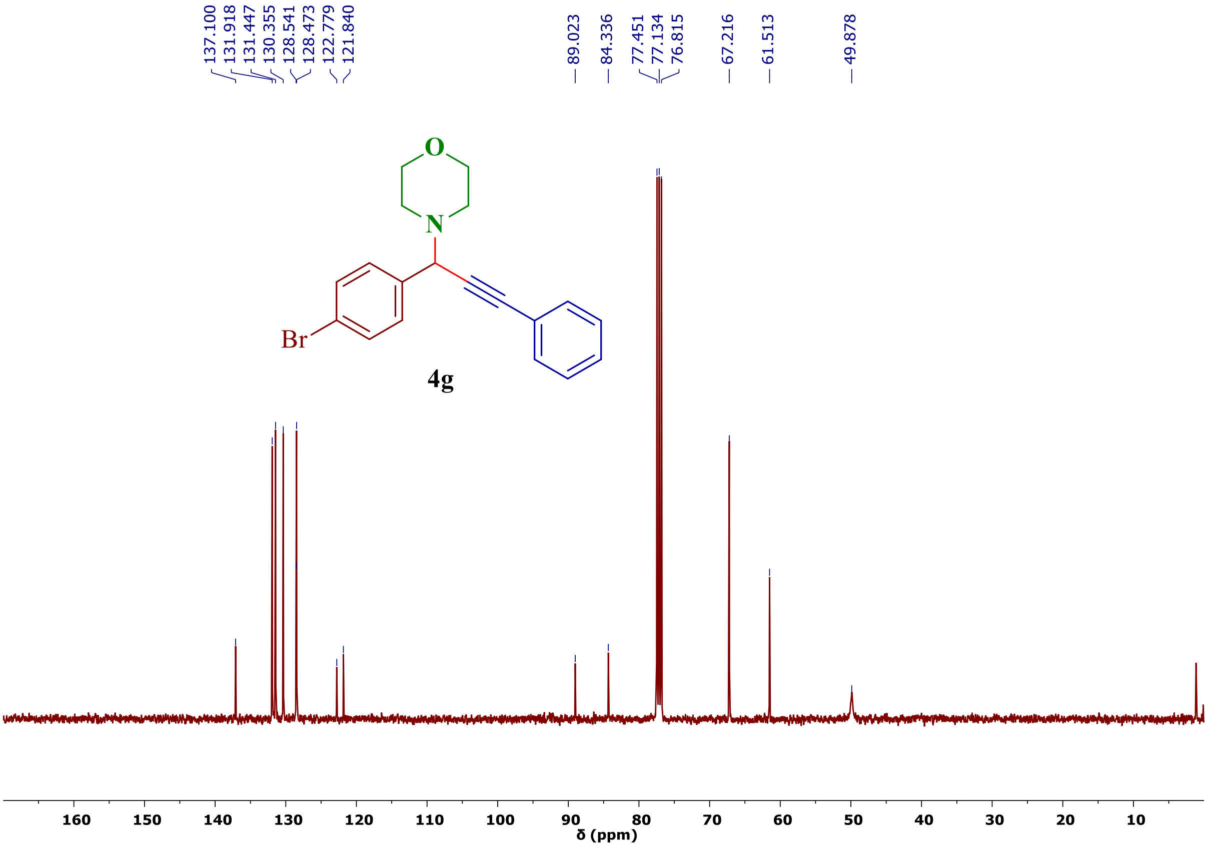

Figure S14. ^{13}C NMR Spectrum of **4g** in $CDCl_3$.

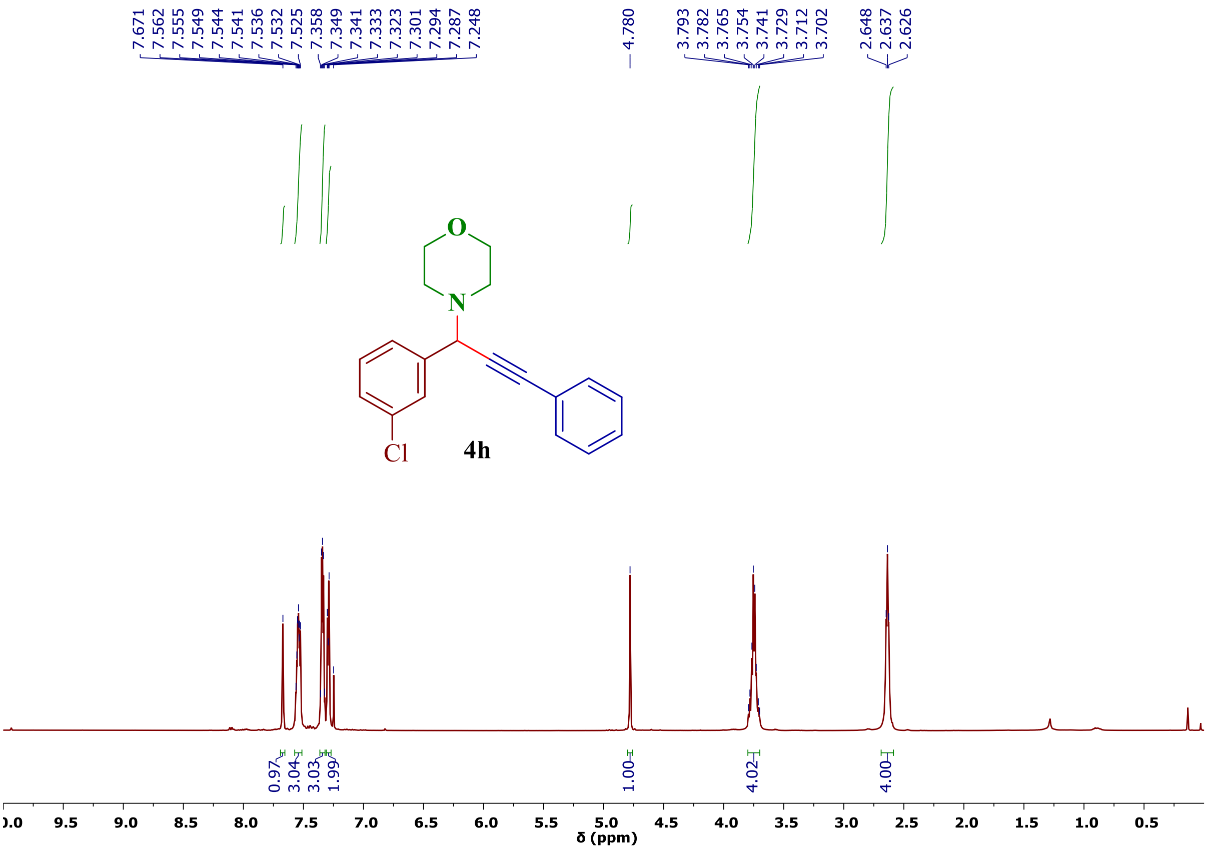

Figure S15. ^{1}H NMR Spectrum of **4h** in $CDCl_3$.

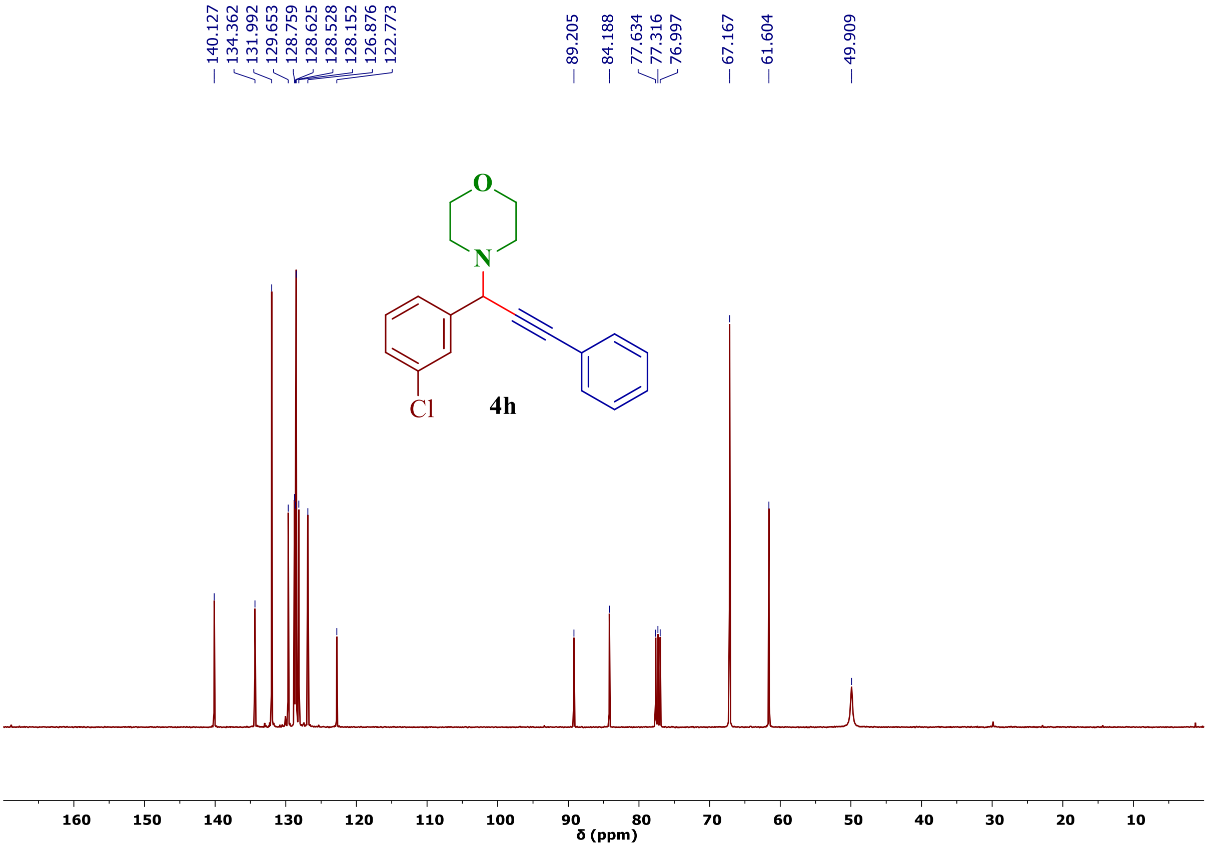

Figure S16. ^{13}C NMR Spectrum of **4h** in $CDCl_3$.

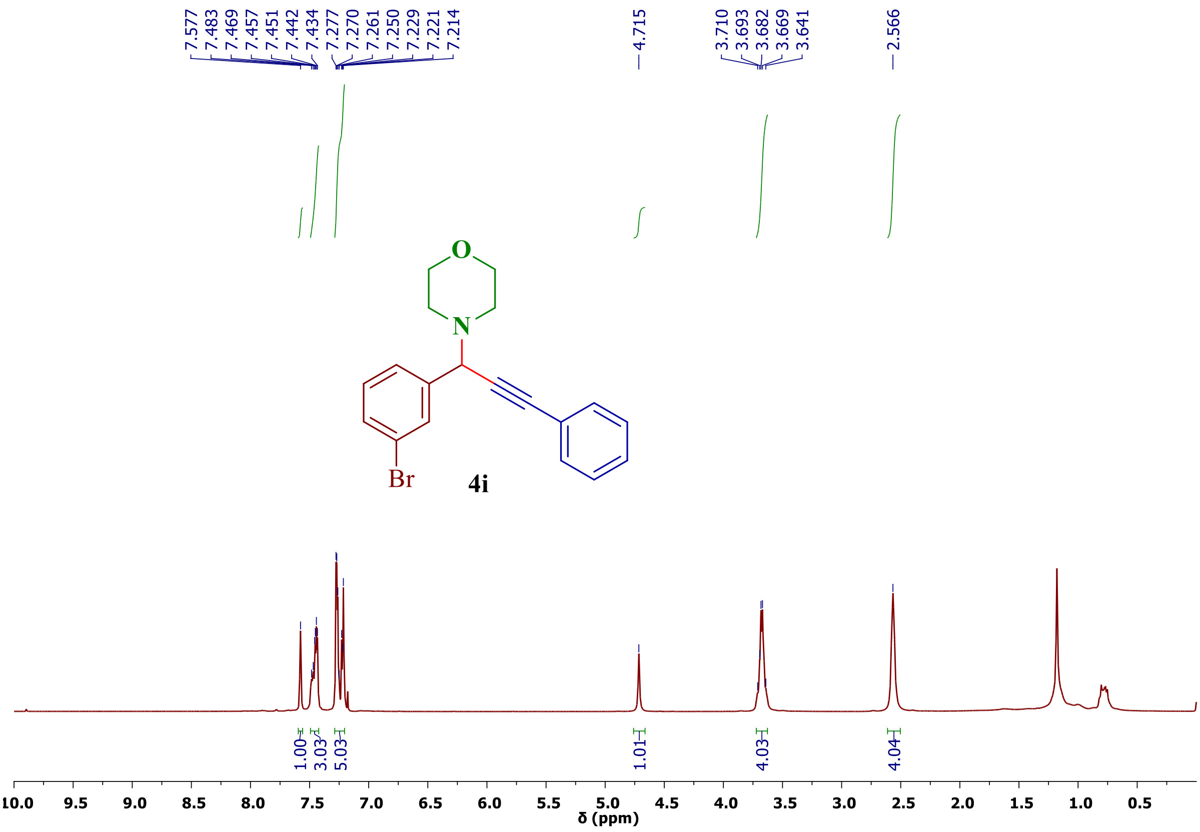

Figure S17. ^{1}H NMR Spectrum of **4i** in $CDCl_3$.

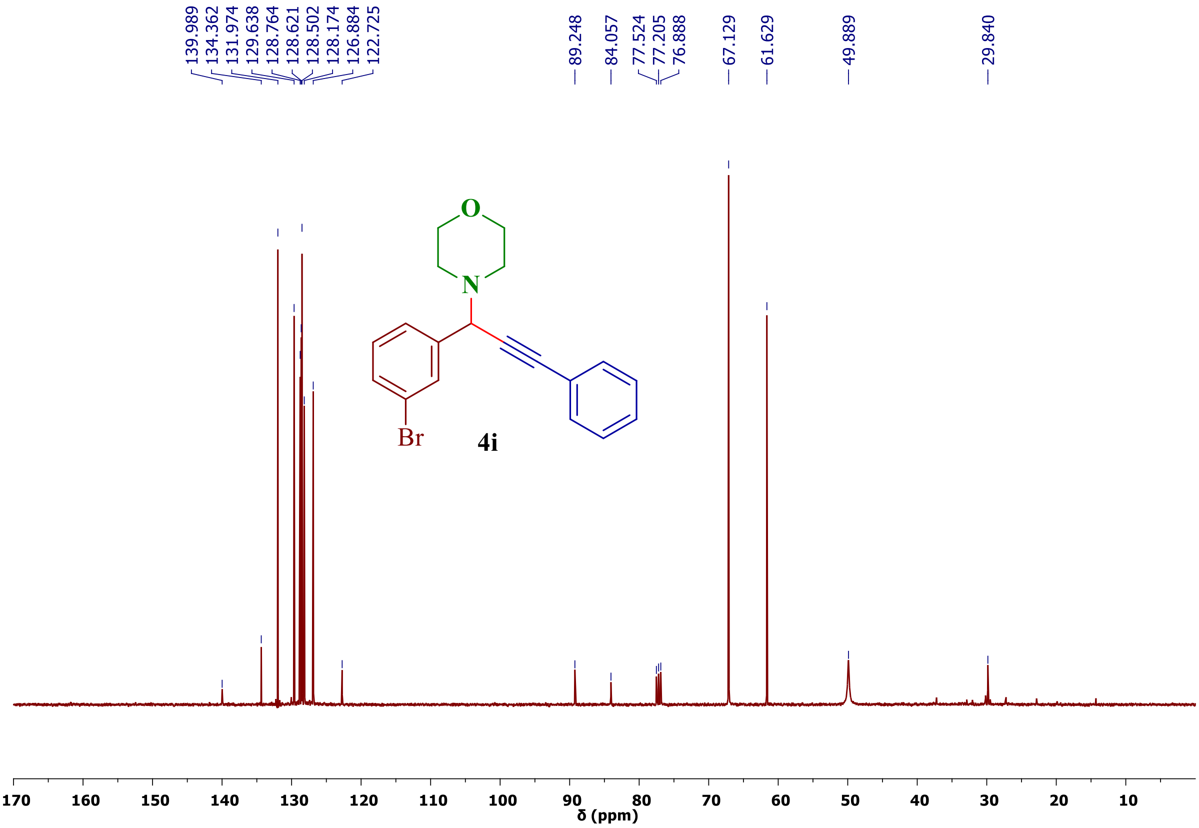

Figure S18. ^{13}C NMR Spectrum of **4i** in $CDCl_3$.

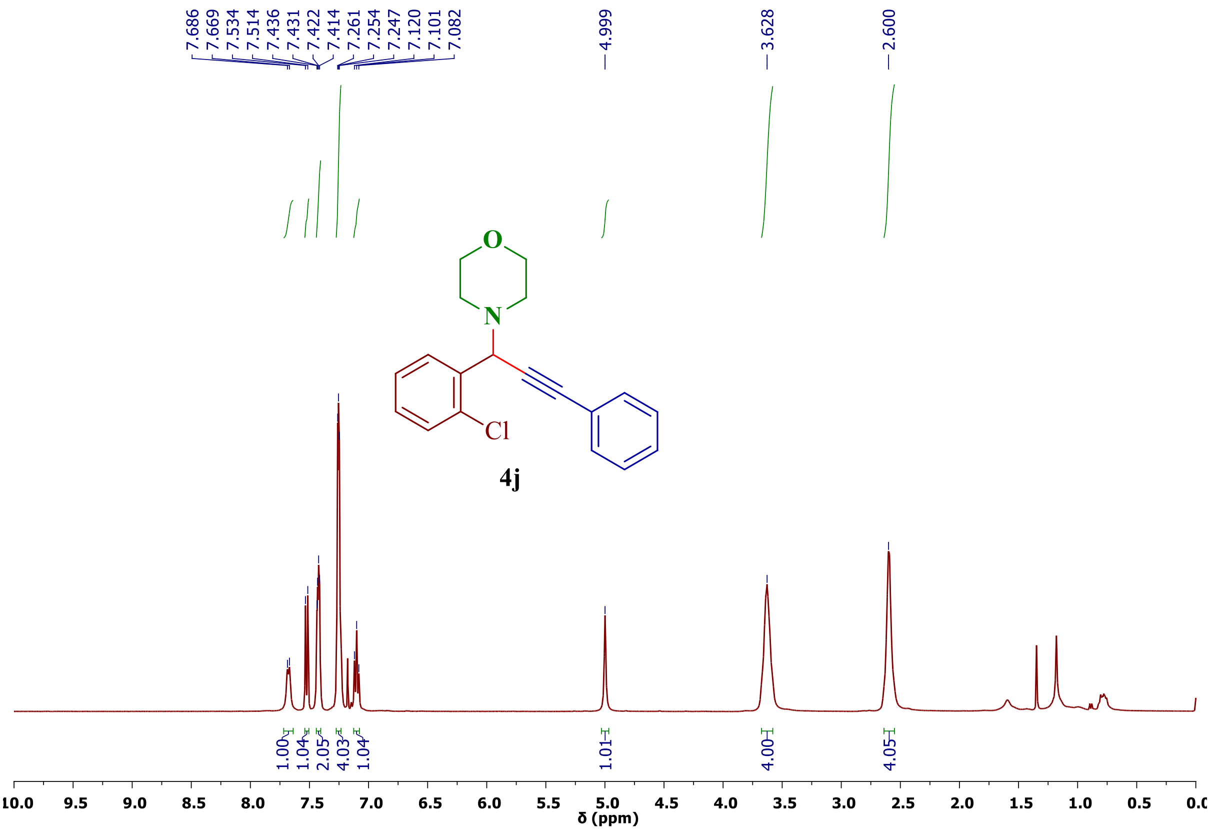

Figure S19. ^{1}H NMR Spectrum of **4j** in $CDCl_3$.

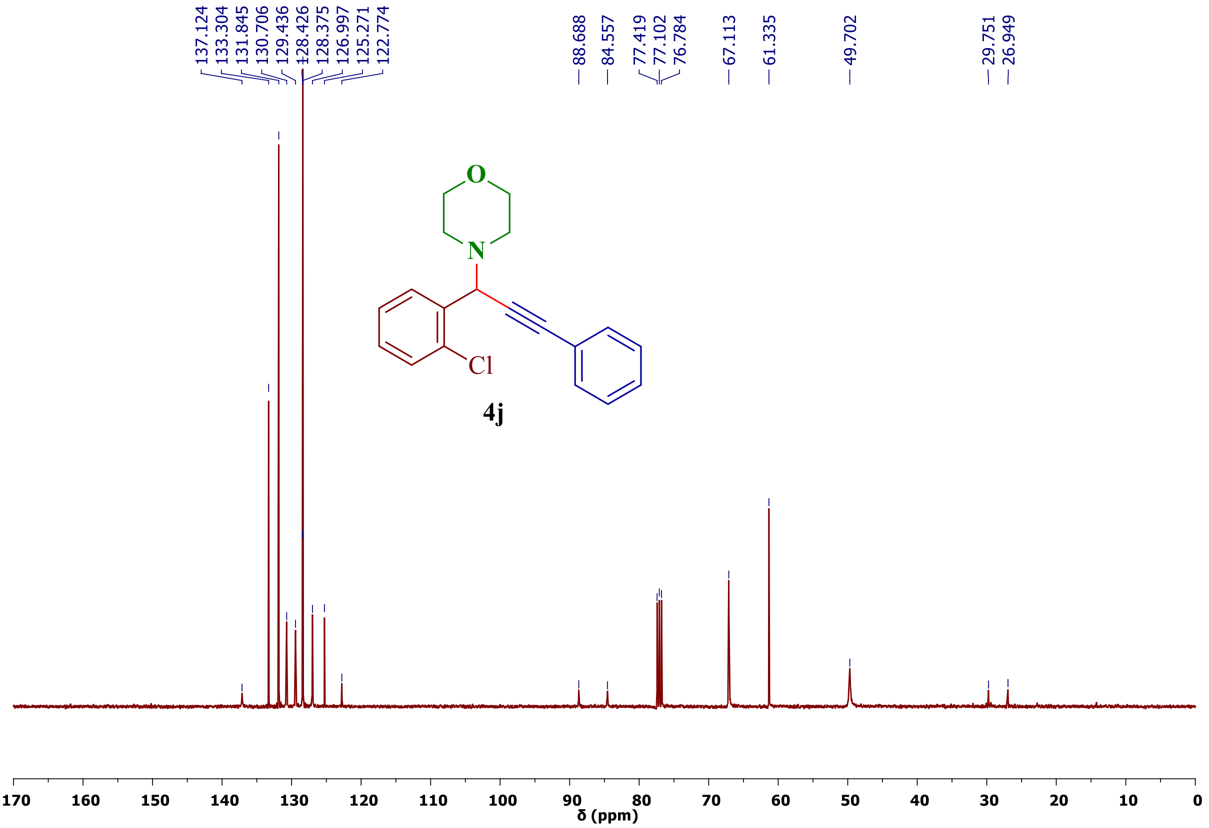

Figure S20. ^{13}C NMR Spectrum of **4j** in $CDCl_3$.

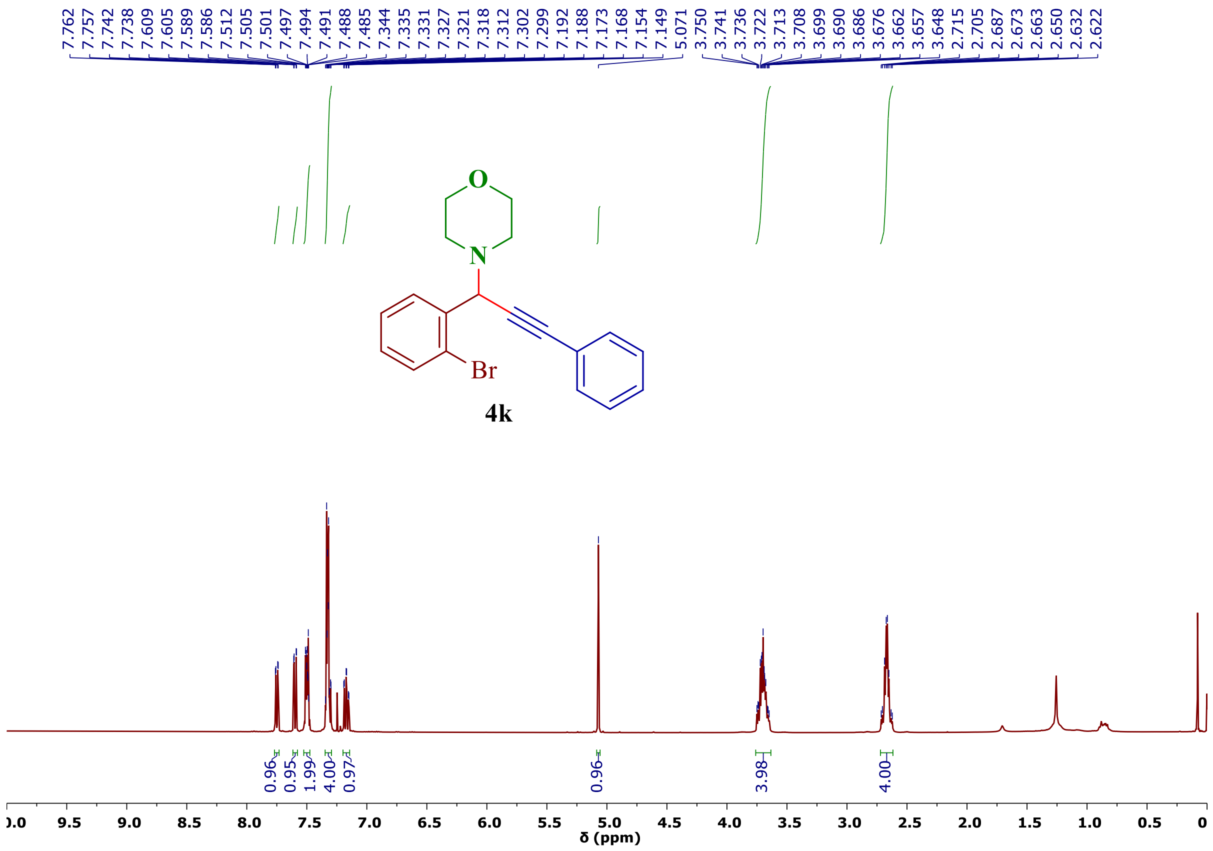

Figure S21. ^{1}H NMR Spectrum of **4k** in $CDCl_3$.

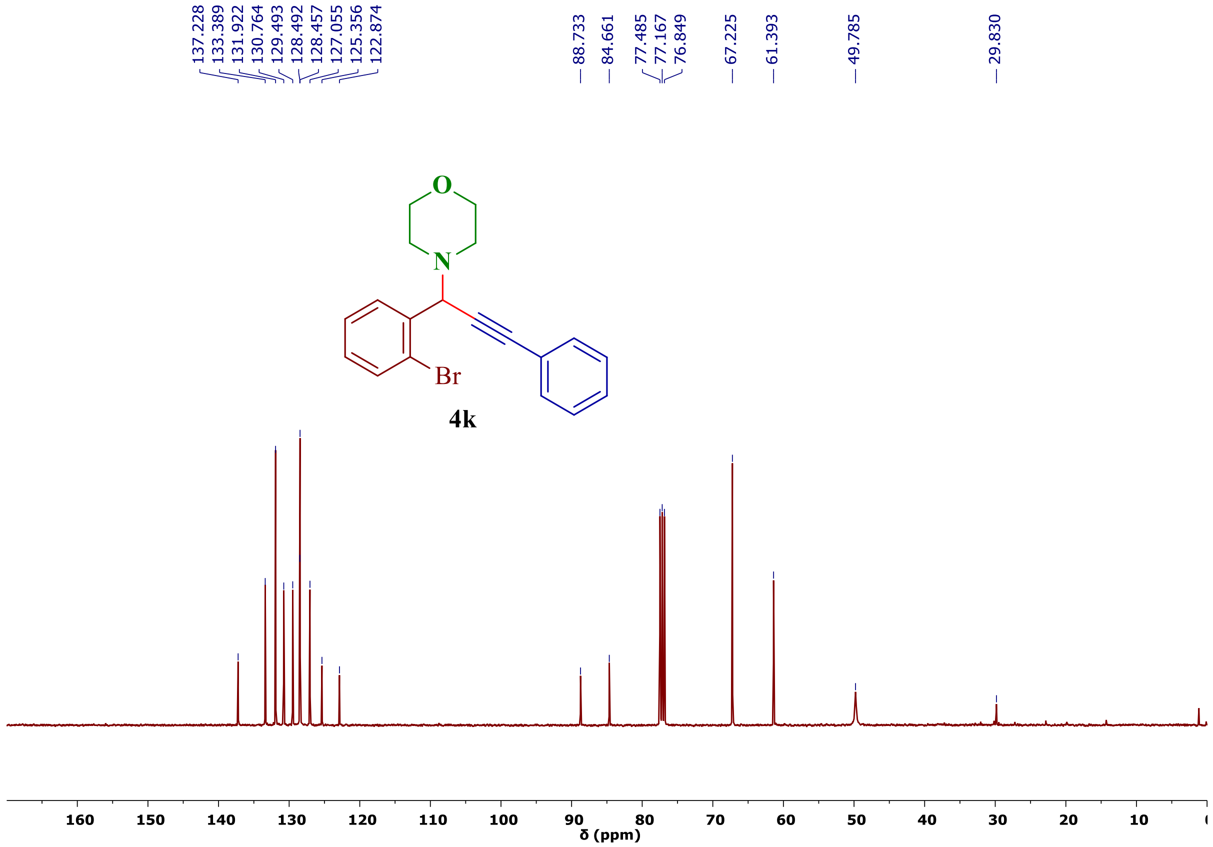

Figure S22. ^{13}C NMR Spectrum of **4k** in $CDCl_3$.

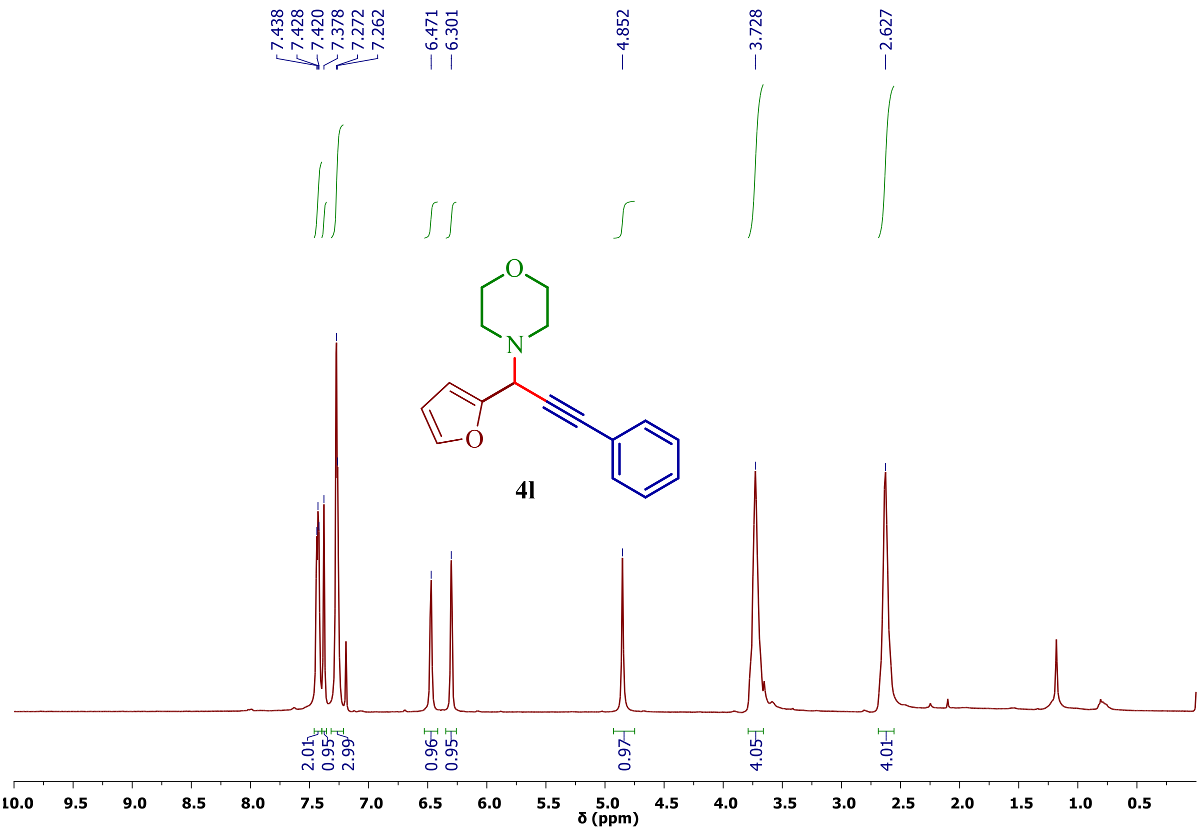

Figure S23. ^{1}H NMR Spectrum of **4l** in $CDCl_3$.

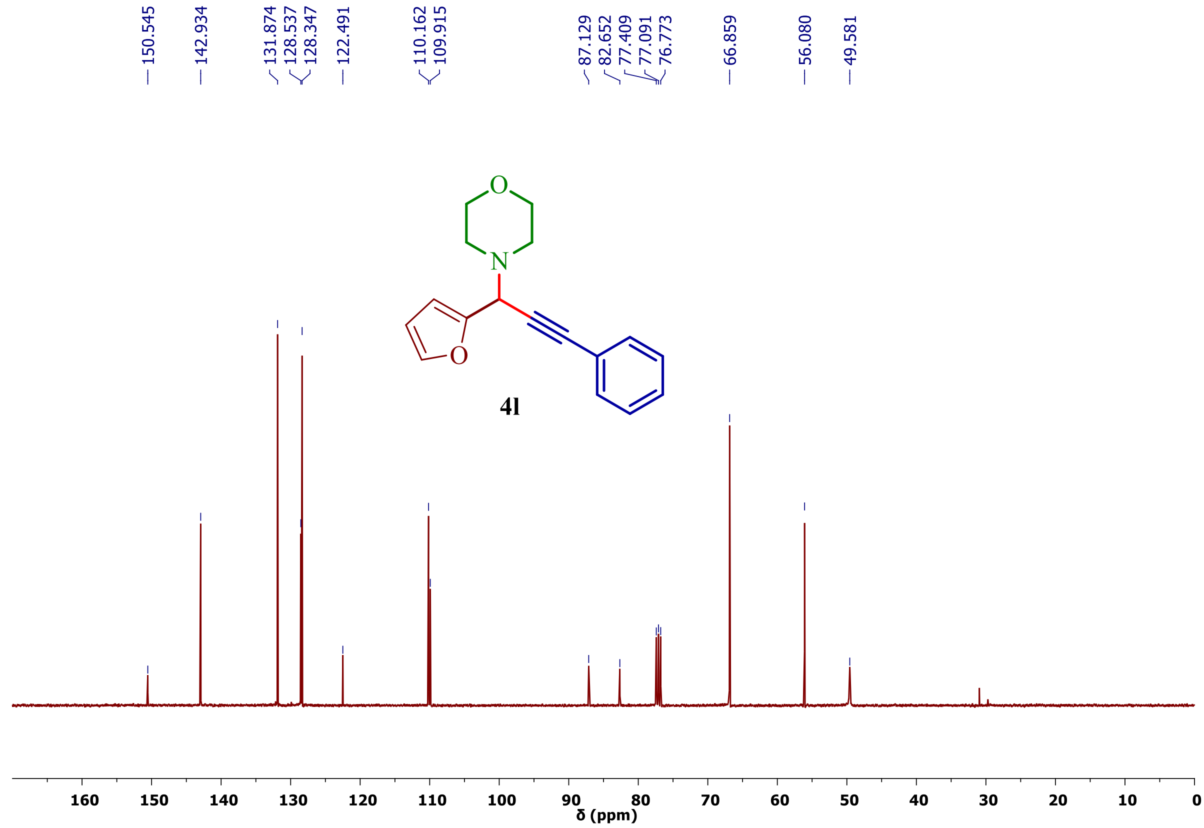

Figure S24. ^{13}C NMR Spectrum of **4l** in $CDCl_3$.

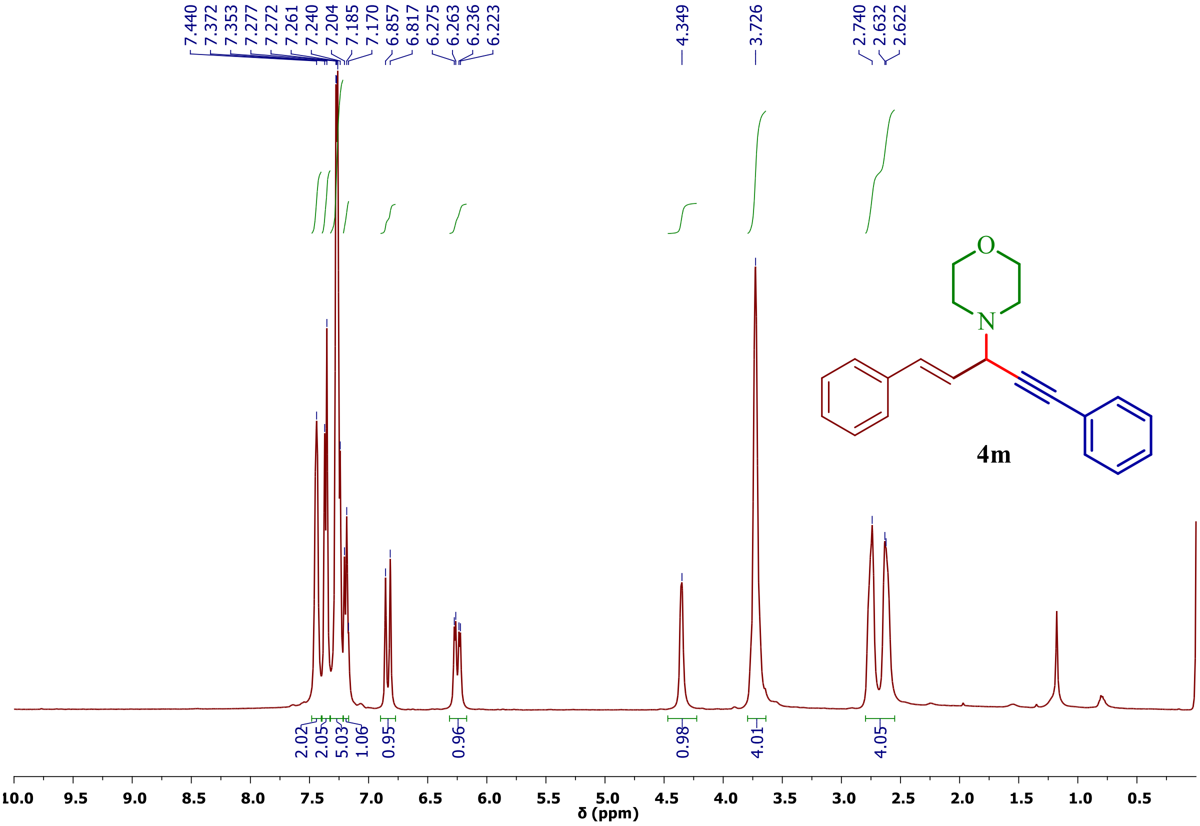

Figure S25. ^{1}H NMR Spectrum of **4m** in $CDCl_3$.

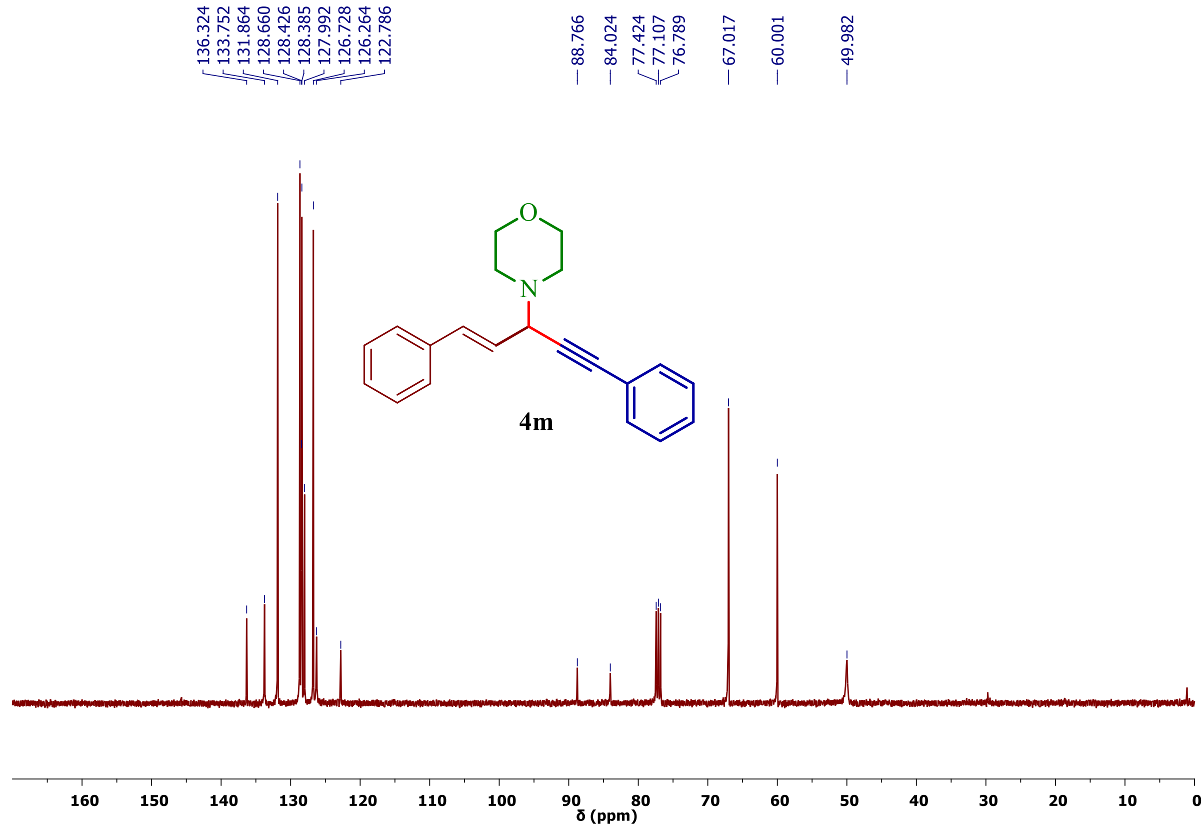

Figure S26. ^{13}C NMR Spectrum of **4m** in $CDCl_3$.

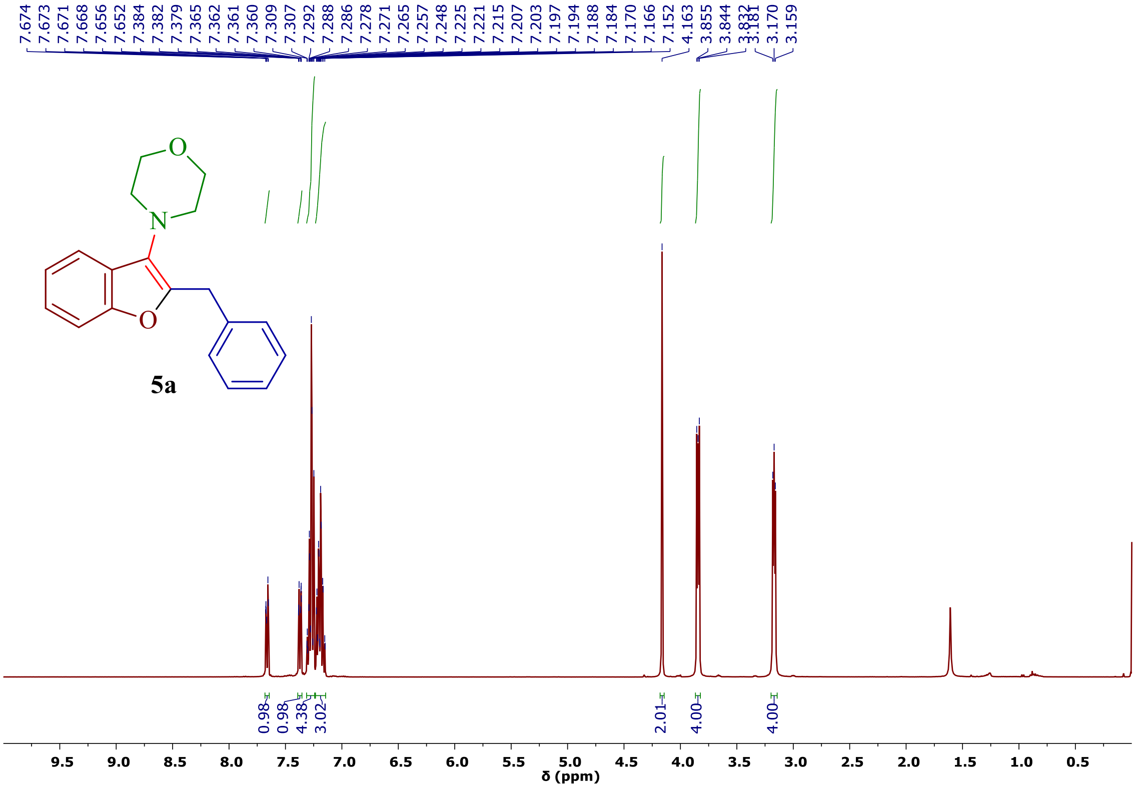

Figure S27. ^{1}H NMR Spectrum of **5a** in $CDCl_3$.

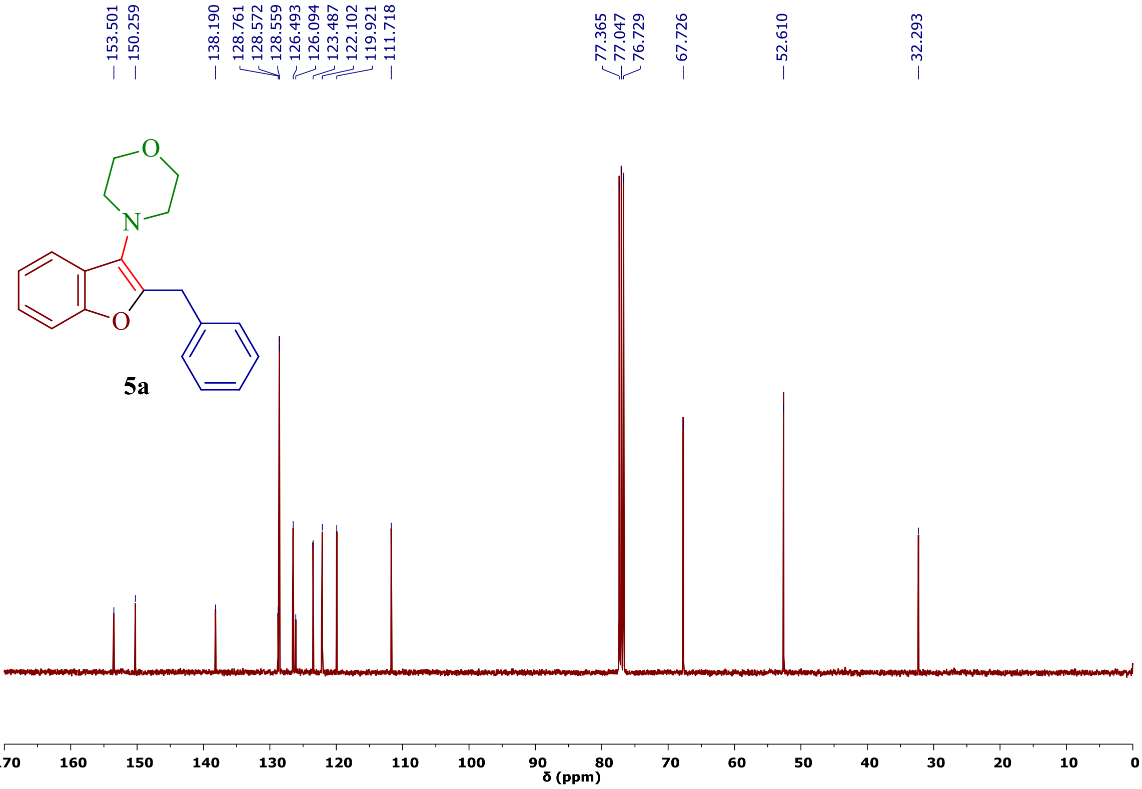

Figure S28. ^{13}C NMR Spectrum of **5a** in $CDCl_3$.

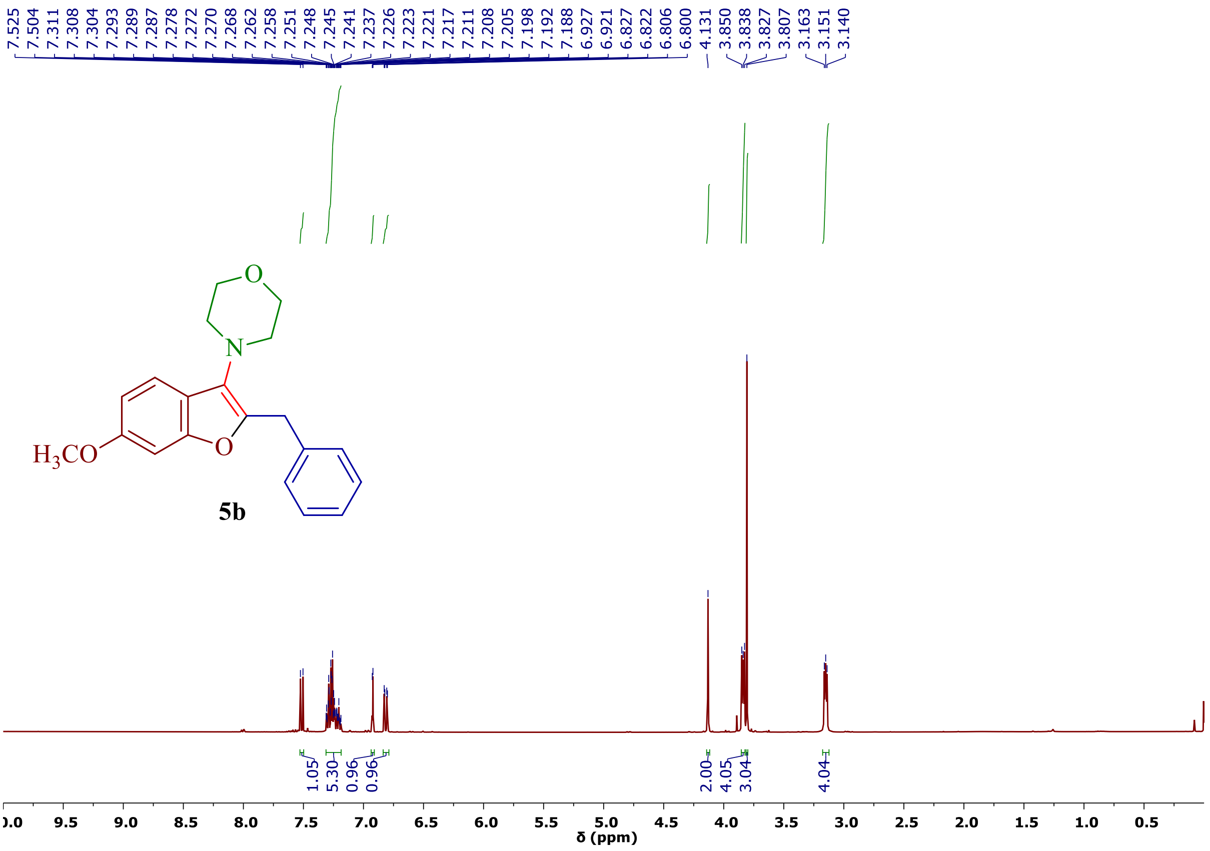

Figure S29. ^{1}H NMR Spectrum of **5b** in $CDCl_3$.

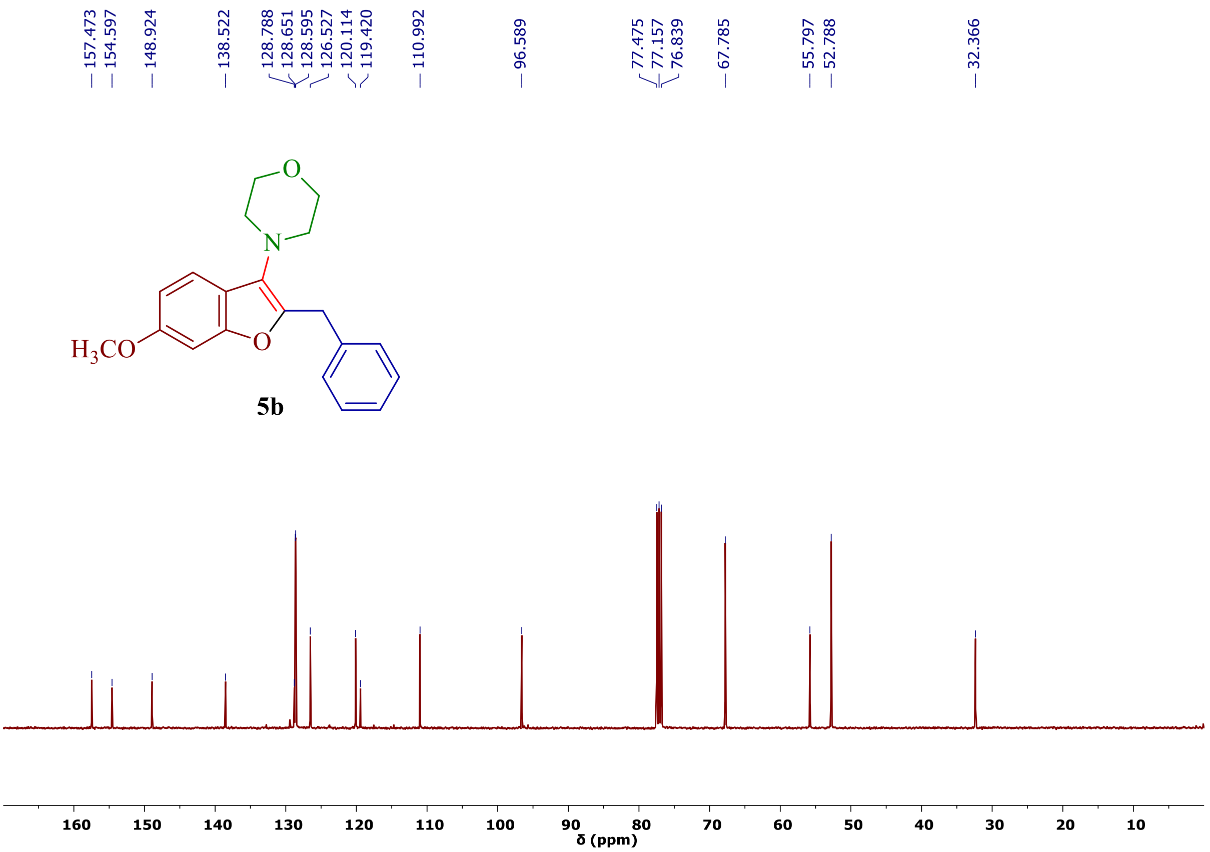

Figure S30. ^{13}C NMR Spectrum of **5b** in $CDCl_3$.

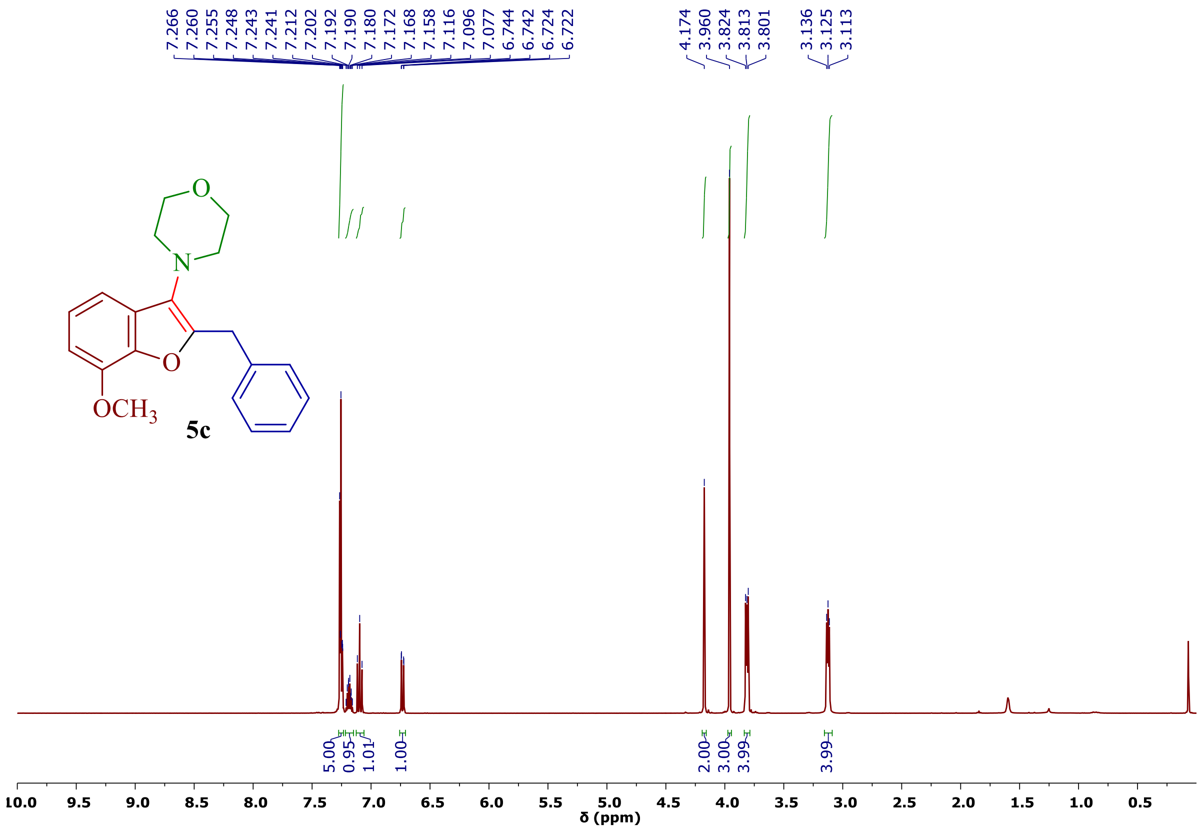

Figure S31. ^{1}H NMR Spectrum of **5c** in $CDCl_3$.

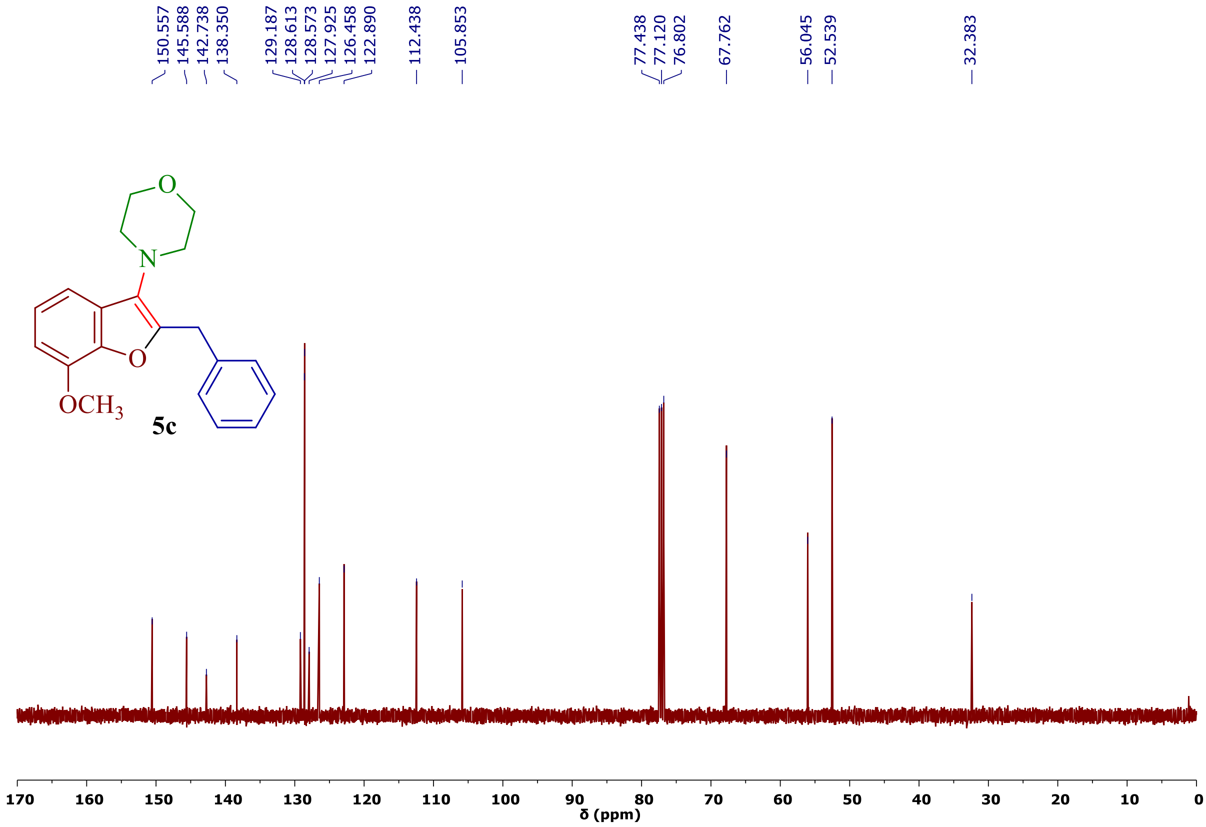

Figure S32. ^{13}C NMR Spectrum of **5c** in $CDCl_3$.

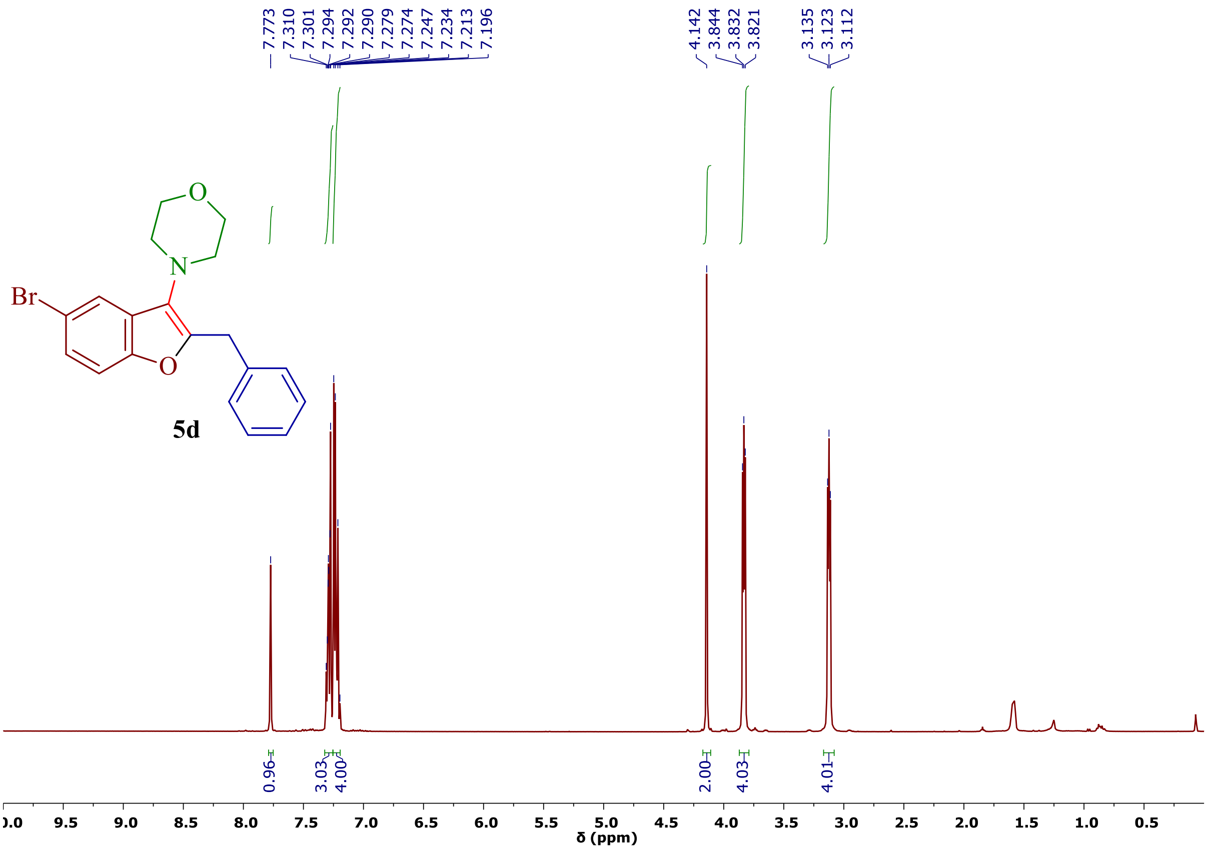

Figure S33. ^{1}H NMR Spectrum of **5d** in $CDCl_3$.

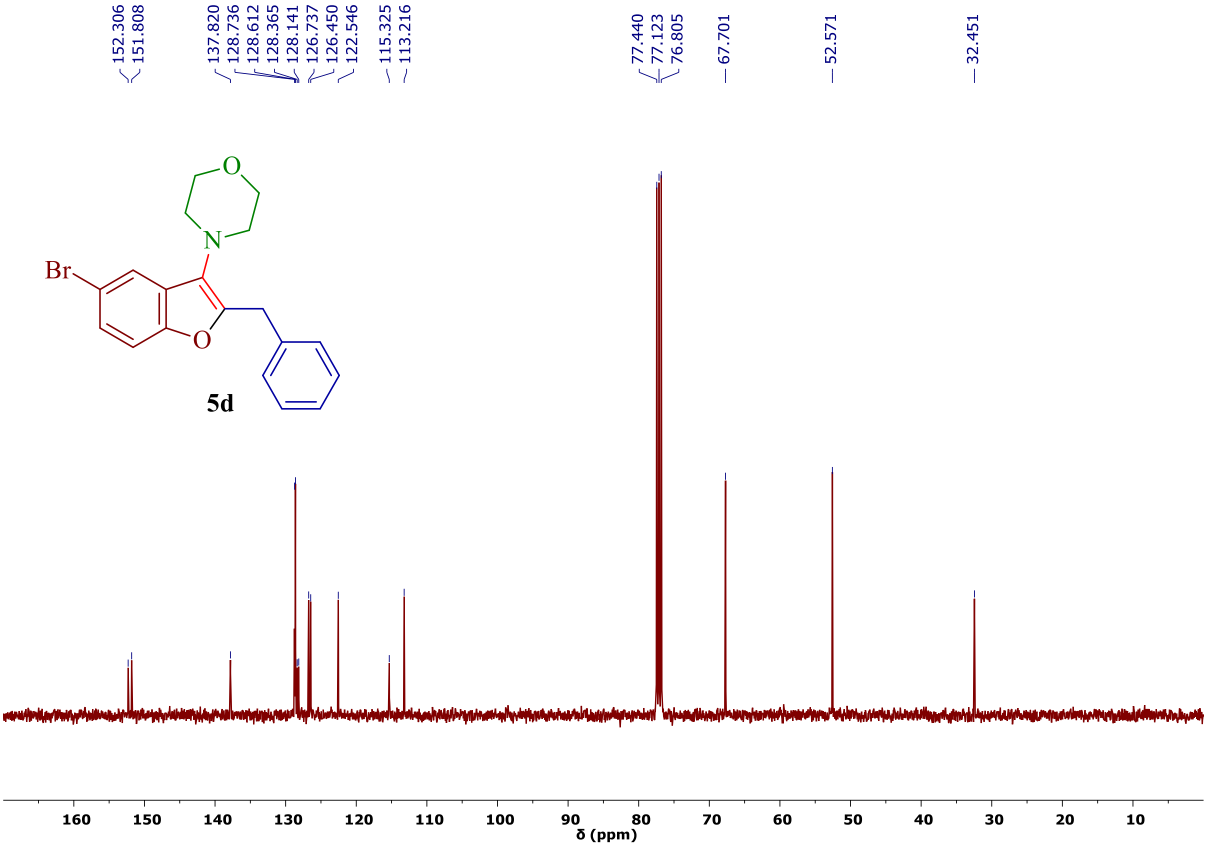

Figure S34. ^{13}C NMR Spectrum of **5d** in $CDCl_3$.

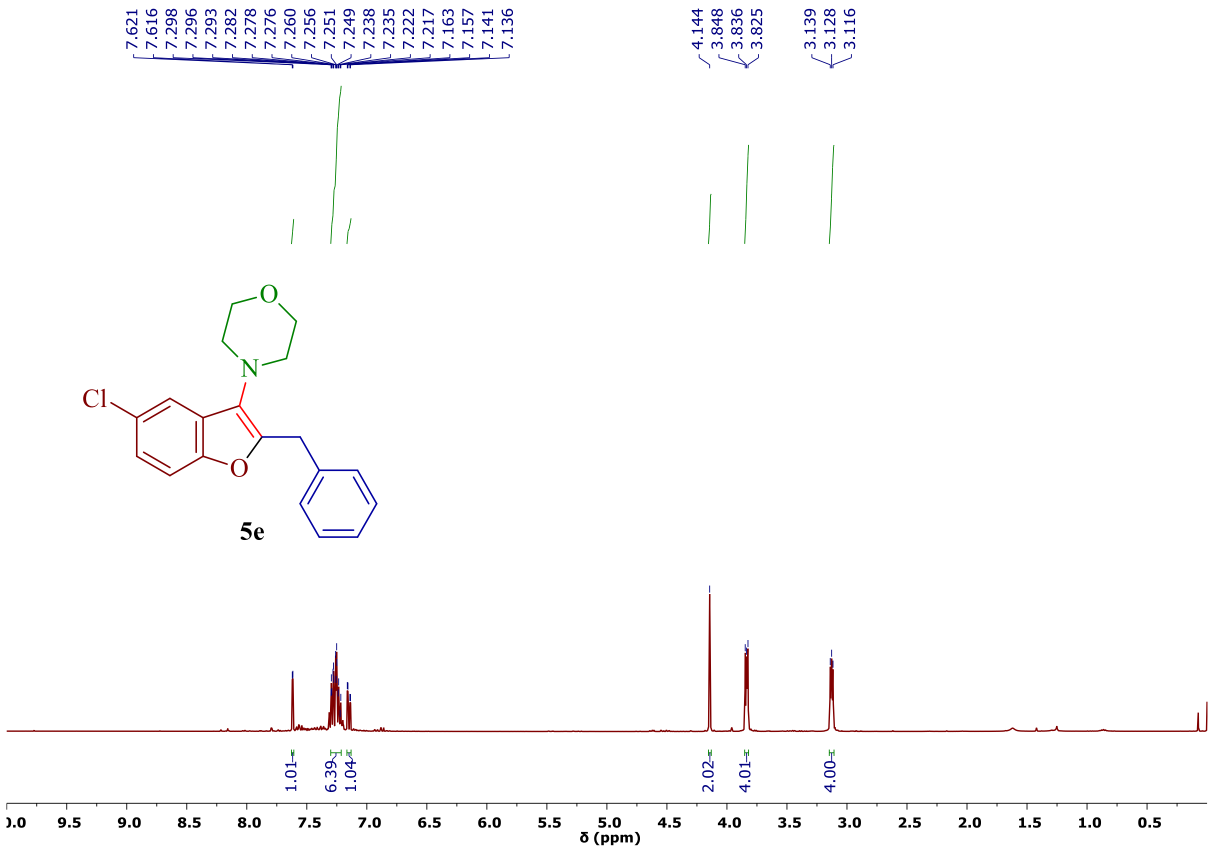

Figure S35. ^{1}H NMR Spectrum of **5e** in $CDCl_3$.

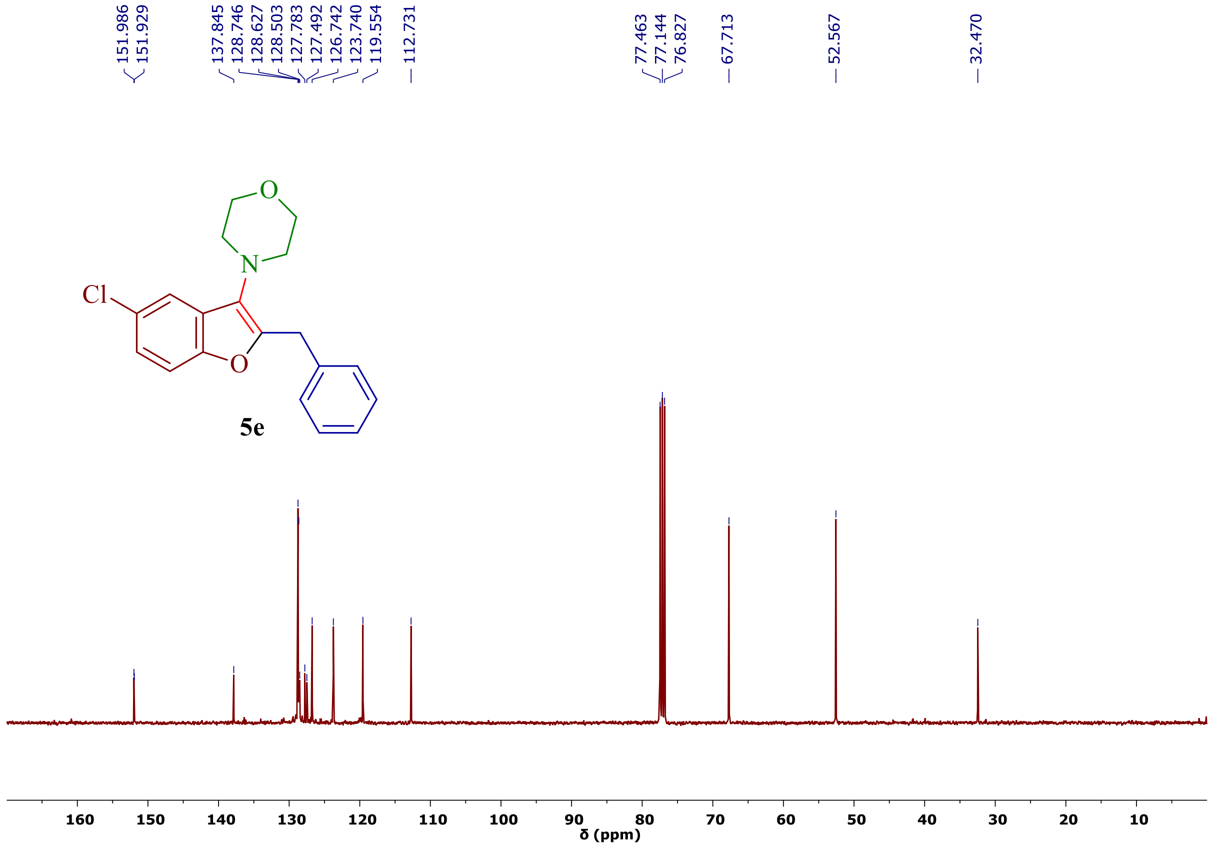

Figure S36. ^{13}C NMR Spectrum of **5e** in $CDCl_3$.

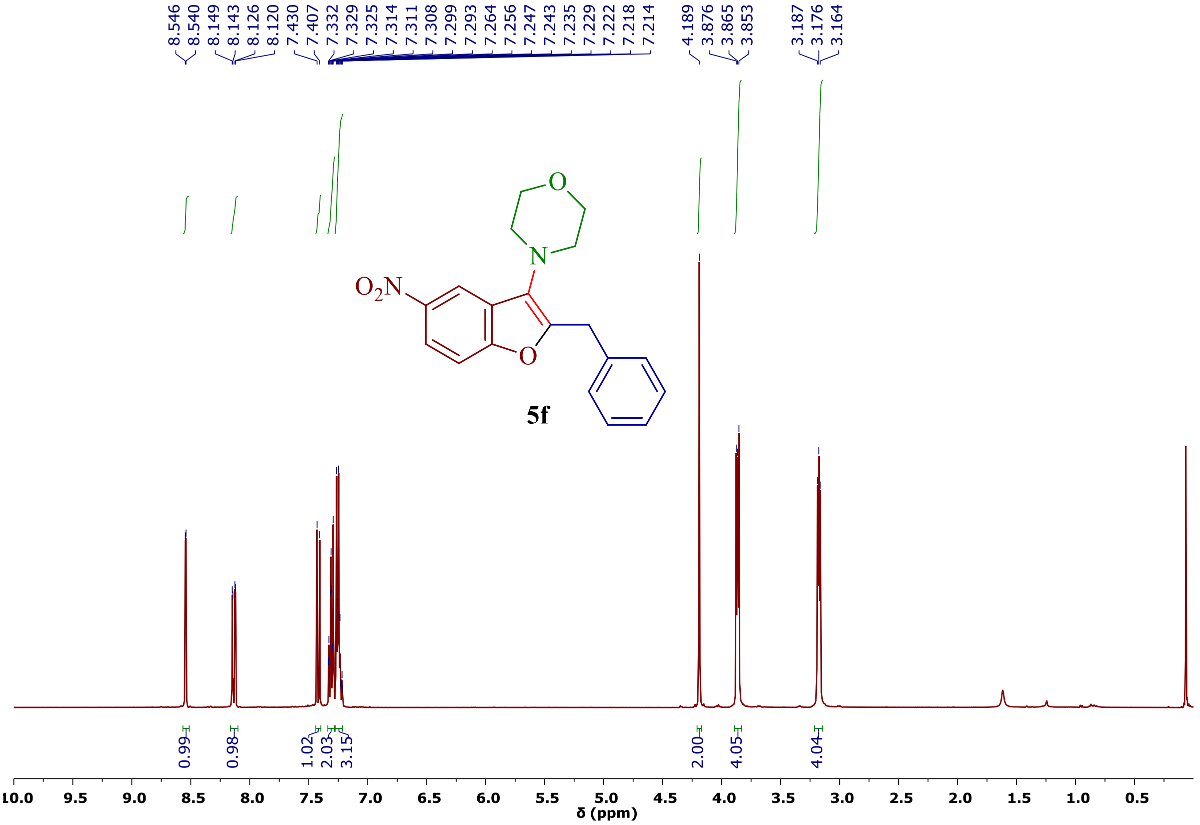

Figure S37. ^{1}H NMR Spectrum of **5f** in $CDCl_3$.

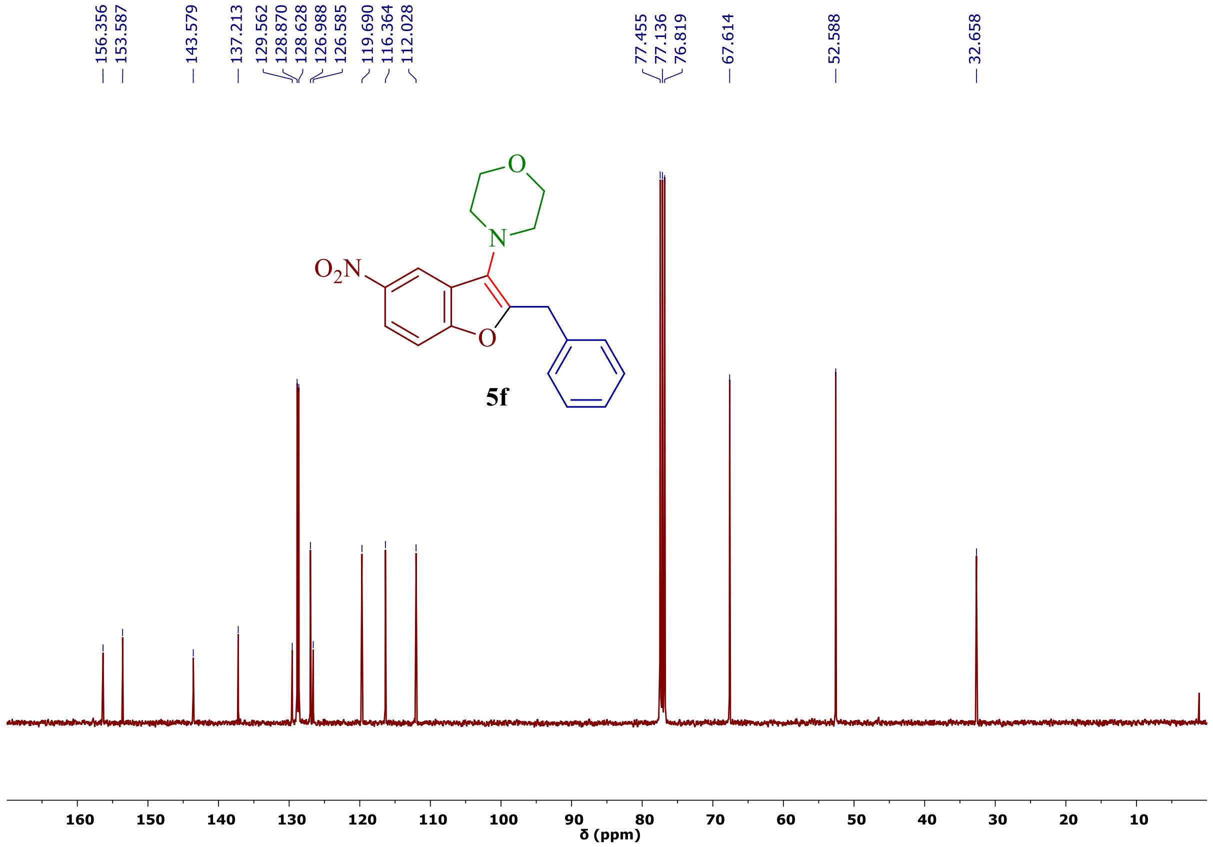

Figure S38. ^{13}C NMR Spectrum of **5f** in $CDCl_3$.

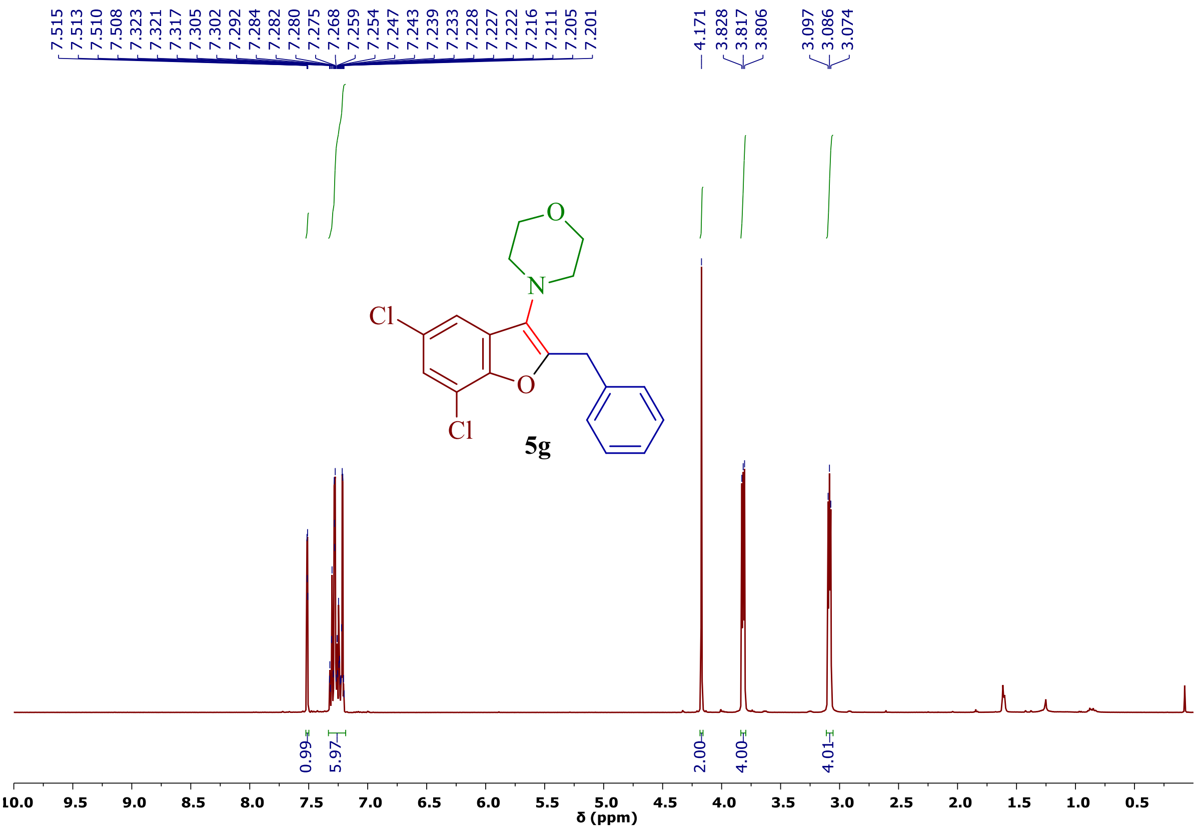

Figure S39. ^{1}H NMR Spectrum of **5g** in $CDCl_3$.

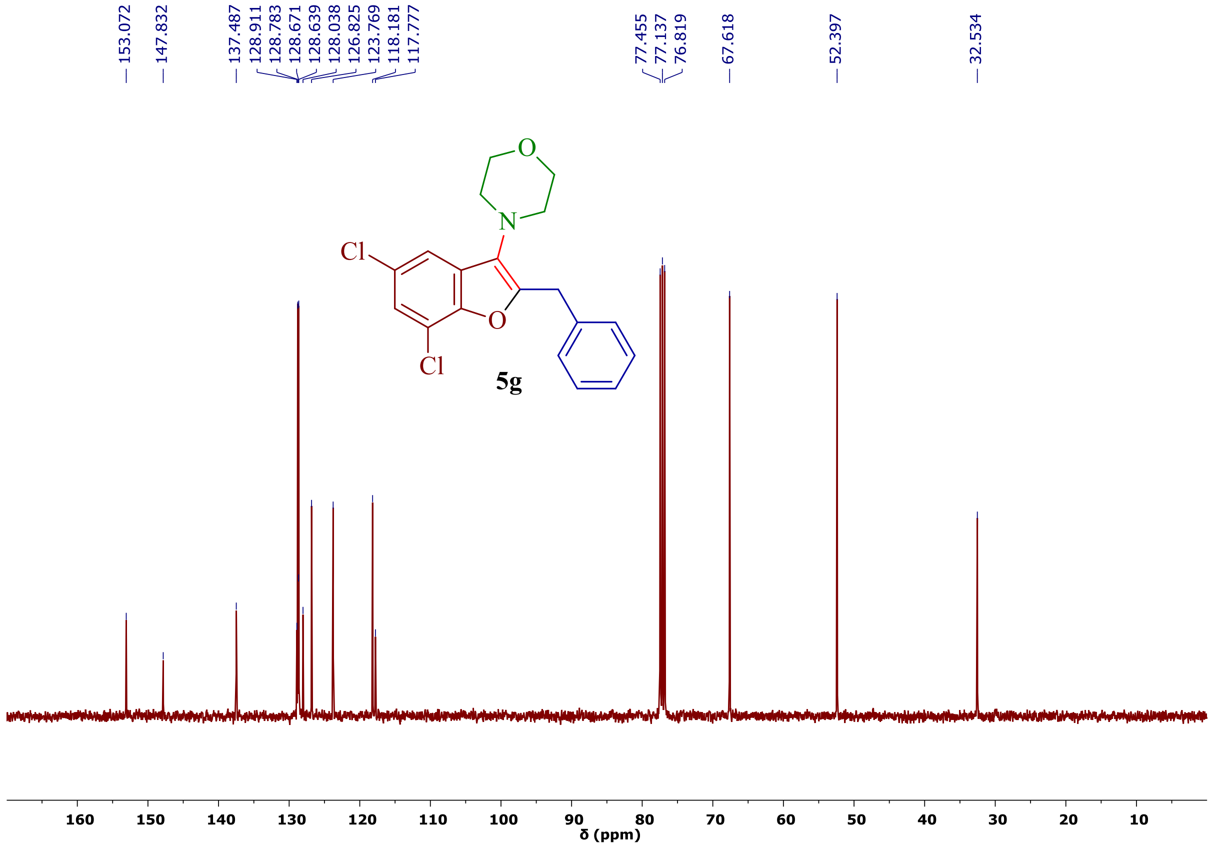

Figure S40. ^{13}C NMR Spectrum of **5g** in $CDCl_3$.

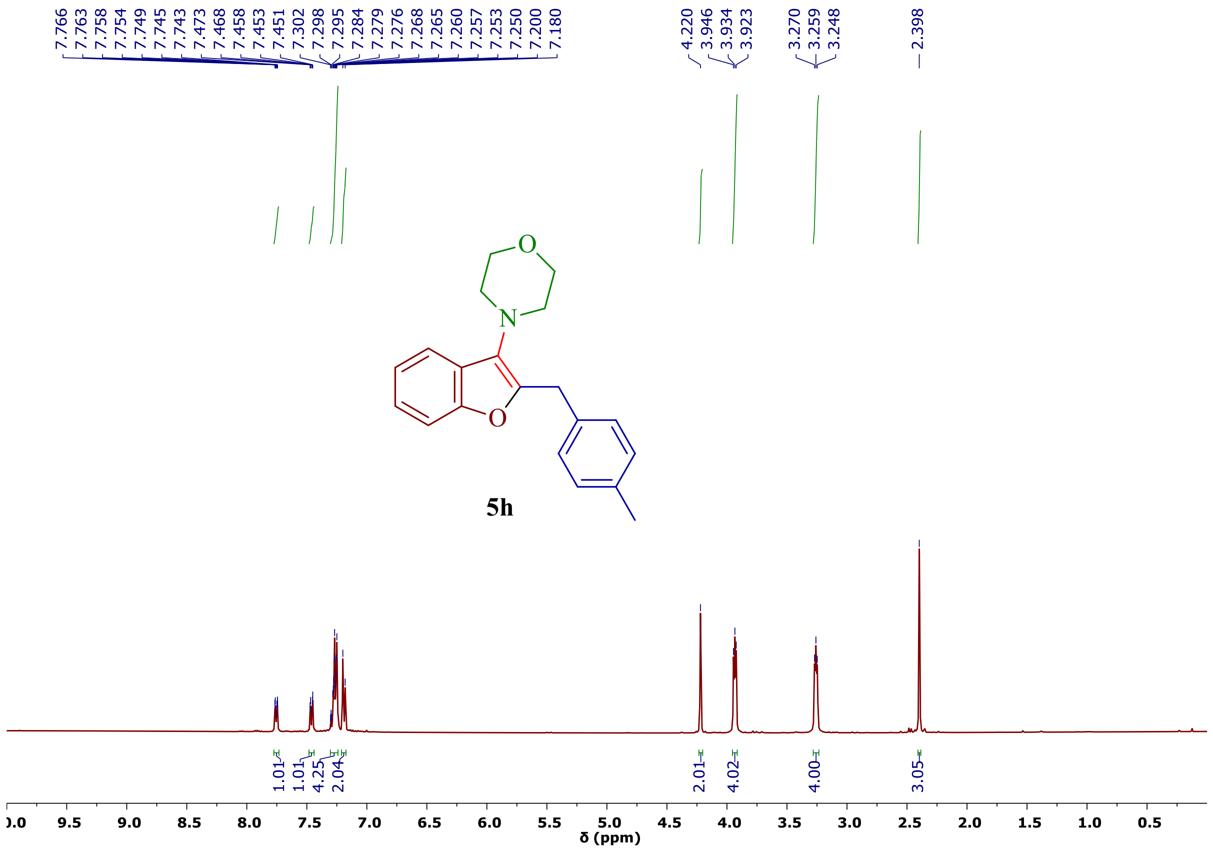

Figure S41. ^{1}H NMR Spectrum of **5h** in $CDCl_3$.

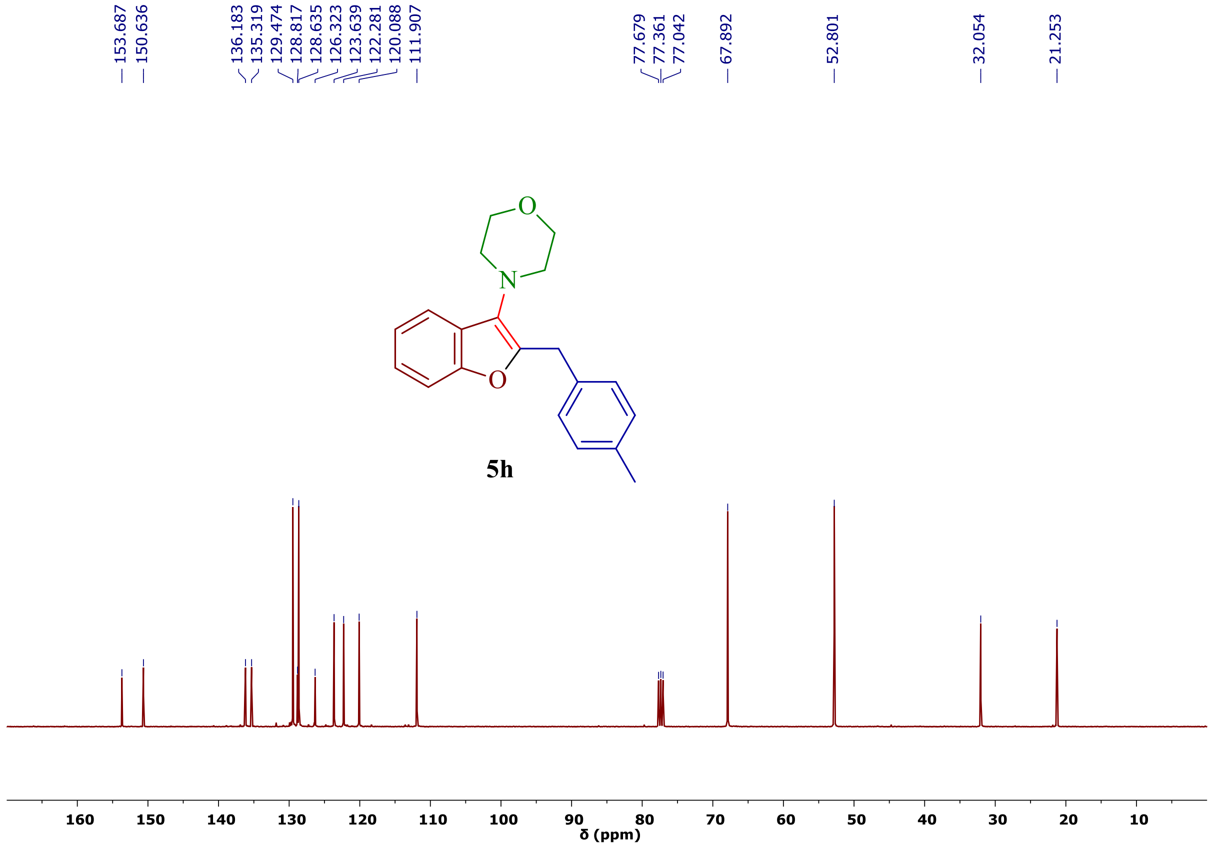

Figure S42. ^{13}C NMR Spectrum of **5h** in $CDCl_3$.

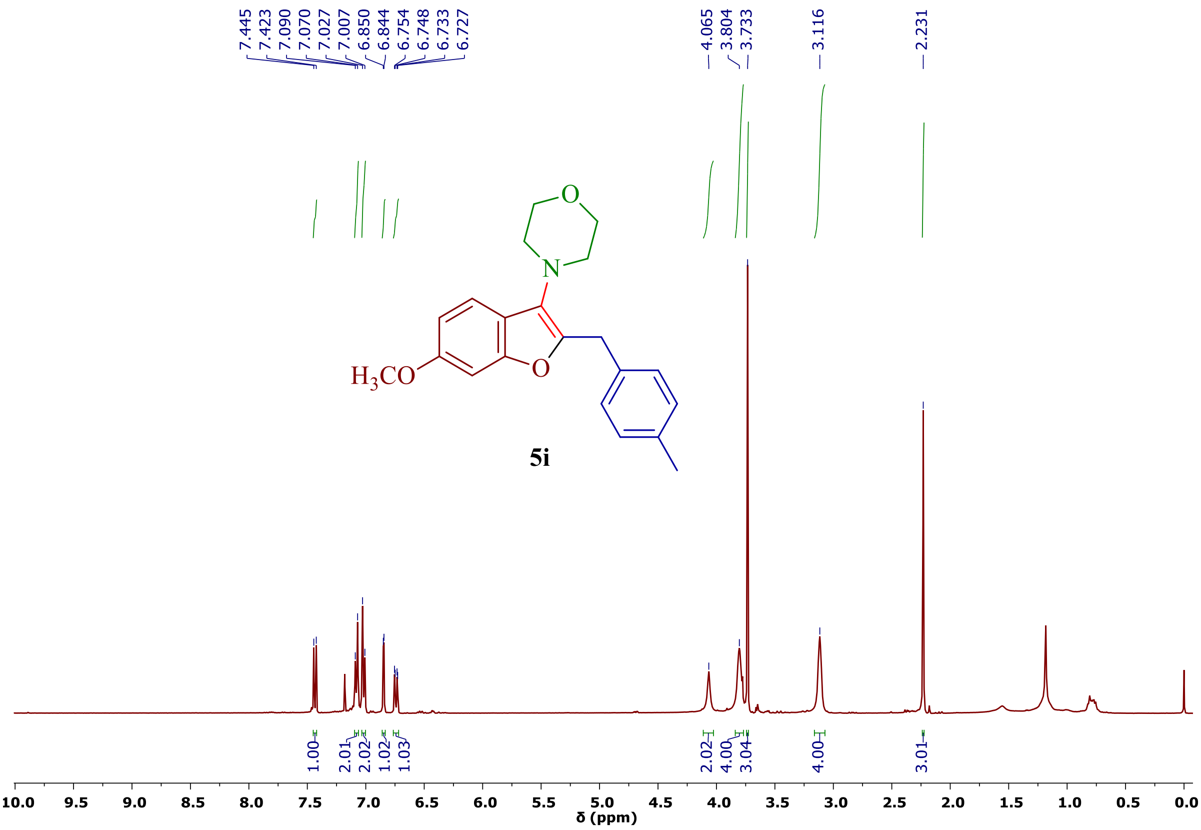

Figure S43. ^{1}H NMR Spectrum of **5i** in $CDCl_3$.

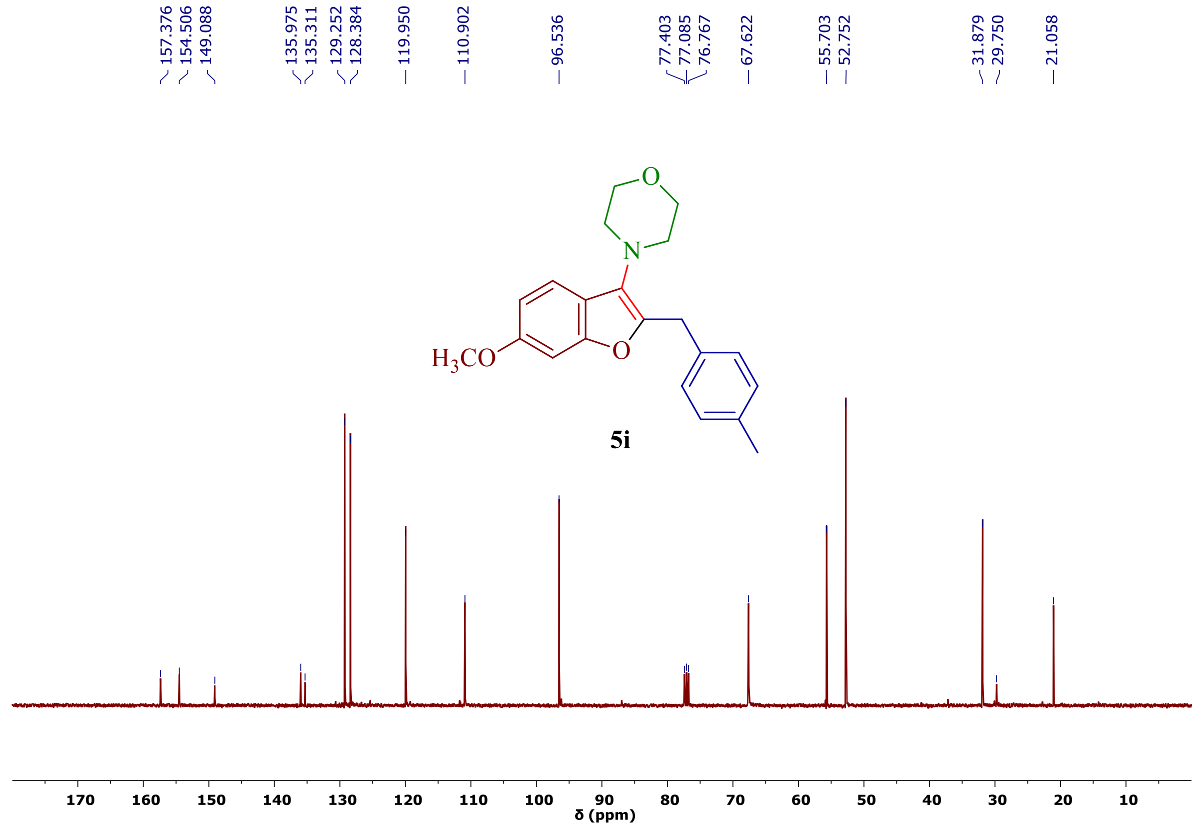

Figure S44. ^{13}C NMR Spectrum of **5i** in $CDCl_3$.

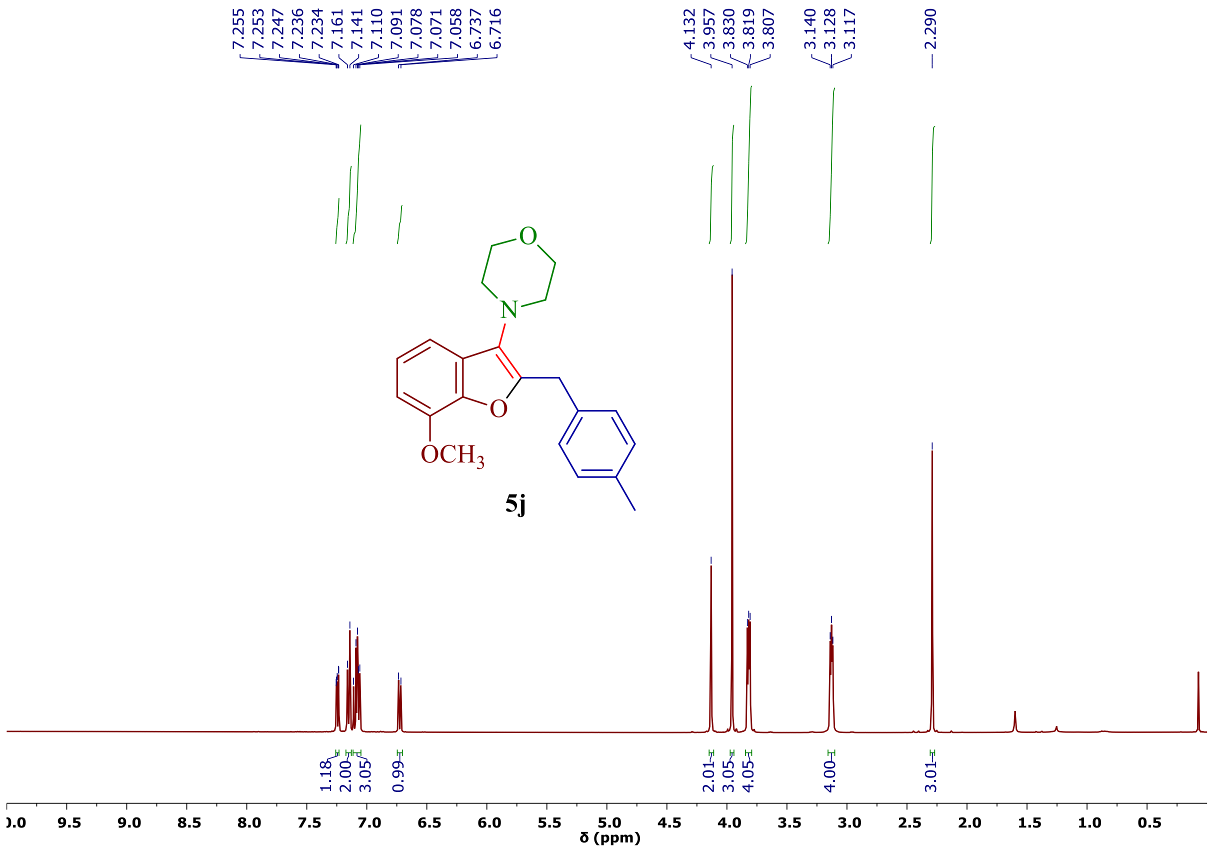

Figure S45. ^{1}H NMR Spectrum of **5j** in $CDCl_3$.

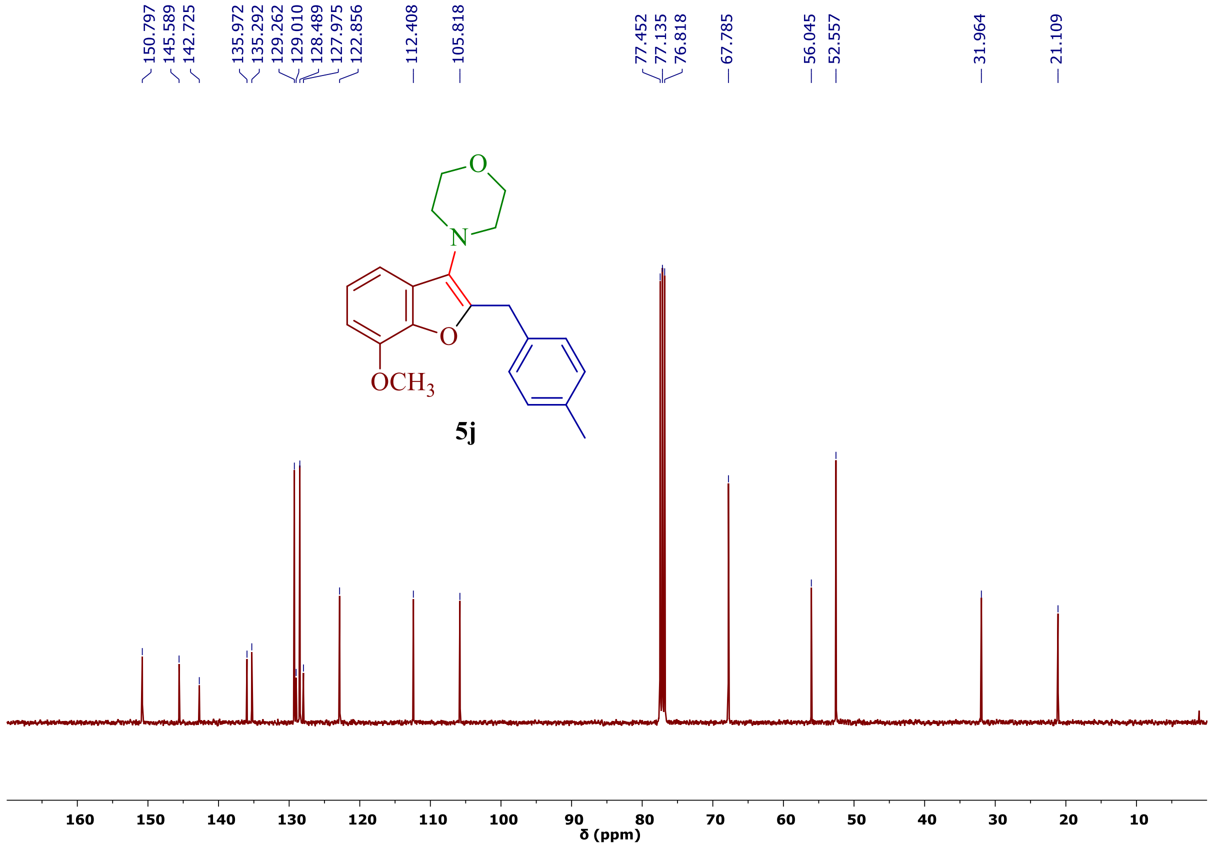

Figure S46. ^{13}C NMR Spectrum of **5j** in $CDCl_3$.

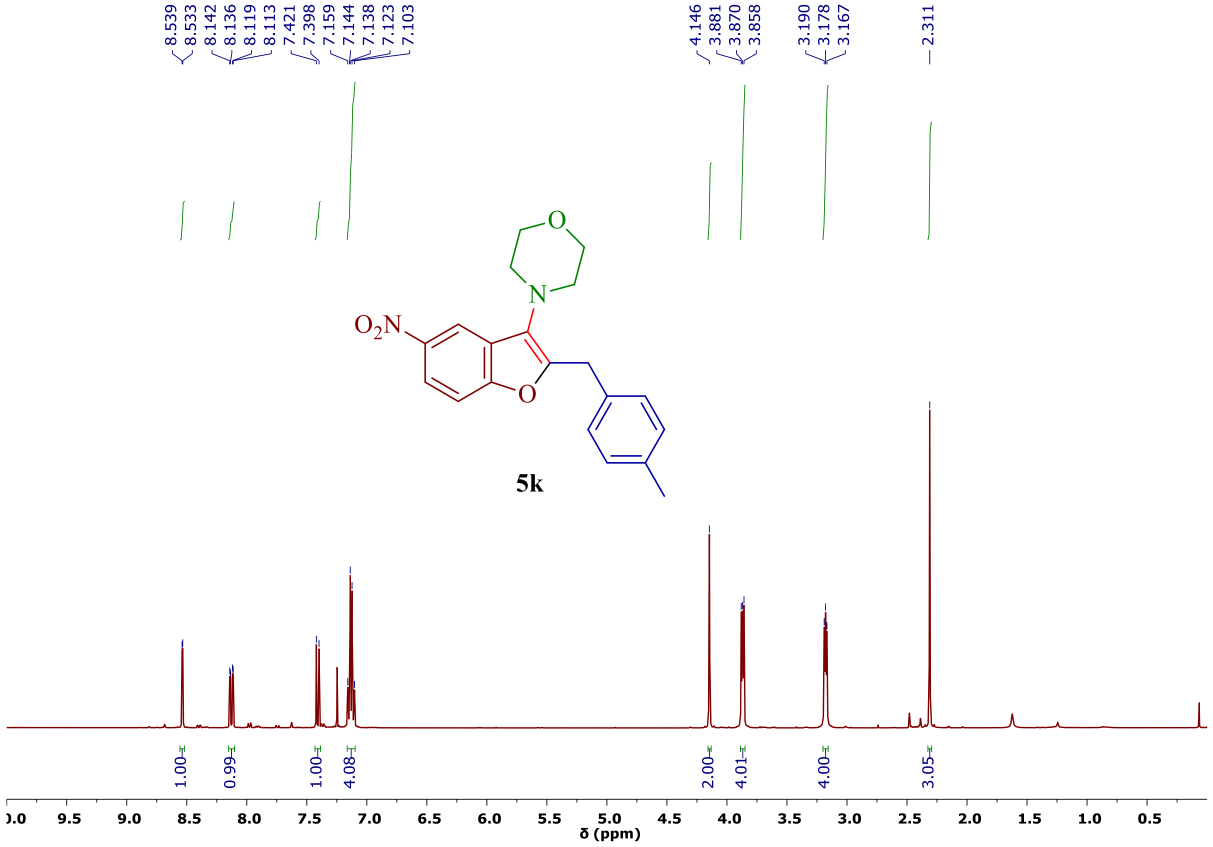

Figure S47. ^{1}H NMR Spectrum of **5k** in $CDCl_3$.

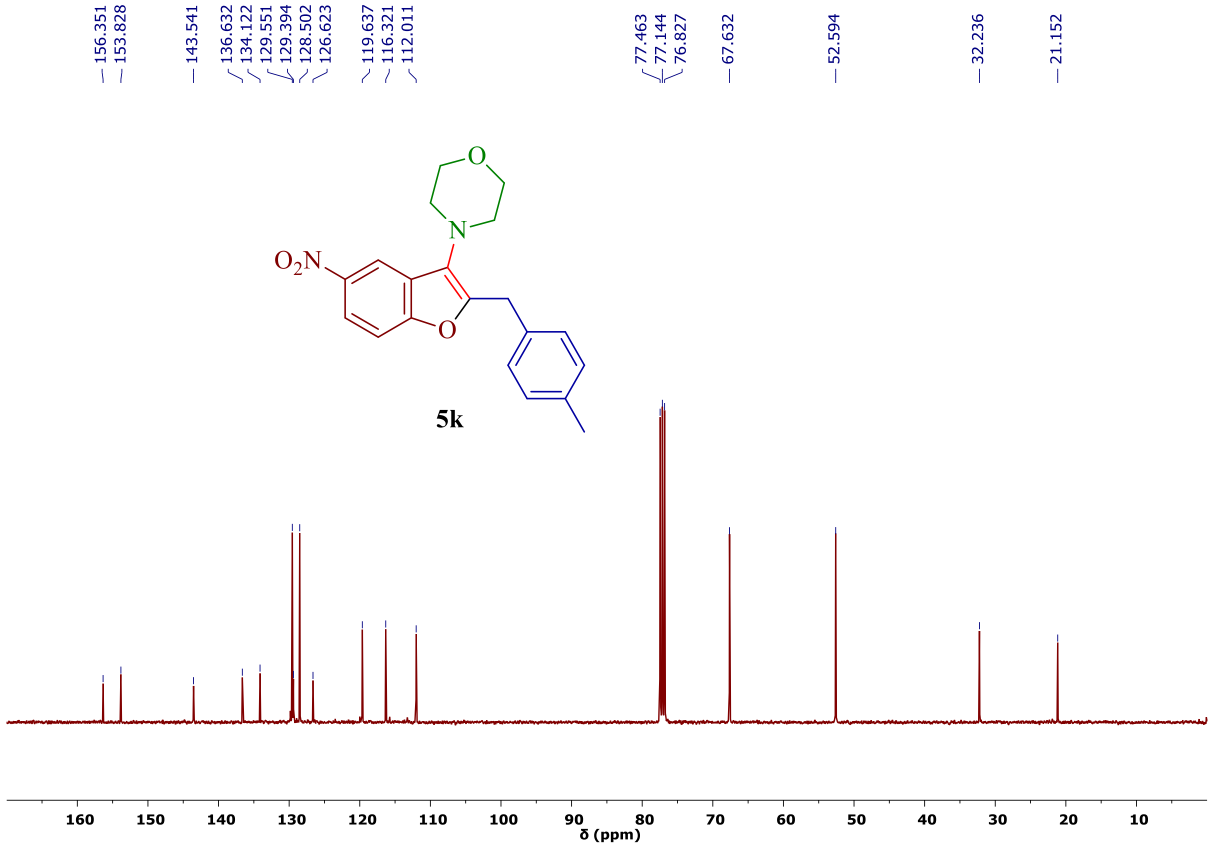

Figure S48. ^{13}C NMR Spectrum of **5k** in $CDCl_3$.
